# AlBr_3_-Promoted stereoselective *anti-*hydroarylation of the acetylene bond in 3-arylpropynenitriles by electron-rich arenes: synthesis of 3,3-diarylpropenenitriles

**DOI:** 10.3762/bjoc.17.180

**Published:** 2021-11-01

**Authors:** Yelizaveta Gorbunova, Dmitry S Ryabukhin, Aleksander V Vasilyev

**Affiliations:** 1 Institute of Chemistry, Saint Petersburg State University, Universitetskaya nab., 7/9, Saint Petersburg, 199034, Russia; 2 All-Russia Research Institute for Food Additives – Branch of V.M. Gorbatov Federal Research Center for Food Systems of RAS, Liteyniy pr., 55, Saint Petersburg, 191014, Russia; 3 Department of Chemistry, Saint Petersburg State Forest Technical University, Institutsky per., 5, Saint Petersburg, 194021, Russia

**Keywords:** aluminum bromide, hydroarylation, indenones, propenenitriles, propynenitriles

## Abstract

Reactions of 3-arylpropynenitriles (ArC≡CCN) with electron-rich arenes (Ar′H, benzene and its polymethylated derivatives) under the action of aluminum bromide (AlBr_3_, 6 equiv) at room temperature for 0.5–2 h result in the stereoselective formation of 3,3-diarylpropenenitriles (Ar(Ar′)C=CHCN) in yields of 20–64%, as products of mainly *anti-*hydroarylation of the acetylene bond. The obtained 3,3-diarylpropenenitriles in triflic acid CF_3_SO_3_H (TfOH) at room temperature for 1 h are cyclized into 3-arylindenones in yields of 55–70%.

## Introduction

Conjugated acetylene nitriles (propynenitriles, R–C≡C–C≡N) are versatile building blocks in organic synthesis for the preparation of a plethora of functionalized compounds and heterocycles. The presence of conjugated acetylene and nitrile bonds in these compounds leads to an enhancement of reactivity of both functional groups. Thus, propynenitriles take part in electrophilic [1–2] and nucleophilic [3–6] addition reactions onto the acetylene bond leading to various substituted nitriles. Reactions onto both acetylene and nitrile groups are widely used for the construction of various heterocyclic systems [7–15].

Recently, we have shown that reactions of 3-arylpropenenitriles (cinnamonitriles, ArCH=CHCN) with arenes (Ar′H) under the superelectrophilic activation by the Brønsted superacid CF_3_SO_3_H (TfOH, triflic acid) or the strong Lewis acid AlBr_3_ result in the formation of 3,3-diarylpropanenitriles (Ar(Ar′)CHCH_2_CN) through the regioselective hydroarylation of the carbon–carbon double bond. In TfOH, the reactions proceed further to 3-arylindanones, as products of the intramolecular aromatic acylation of the 3,3-diarylpropanenitriles by the electrophilically activated nitrile group [16–17]. Based on this study and our work on electrophilic transformations of alkynes [18–20], we investigated reactions of 3-arylpropynenitriles under electrophilic activation conditions (see [21] for the chemistry of superelectrophilic species). The goal of this work was to study the reactions of 3-arylpropynenitriles with arenes under the action of the strong Lewis acids, aluminum halogenides AlX_3_ (X = Cl, Br) and the Brønsted superacid TfOH (CF_3_SO_3_H).

## Results and Discussion

**Scheme 1:**
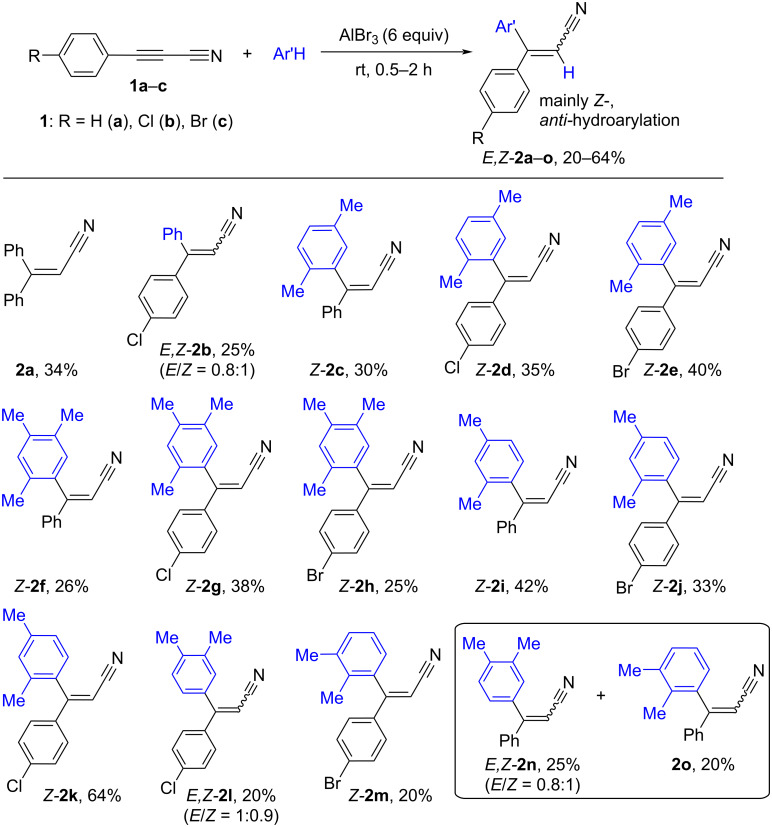
AlBr_3_-promoted hydroarylation of the acetylene bond of 3-arylpropynenitriles **1a–c** by arenes with the formation of 3,3-diarylpropenenitriles *E-,Z-***2a–o**.

It was found that acetylene nitriles **1a–c** reacted with arenes in the presence of excess AlBr_3_ (6 equiv) at room temperature for 0.5–2 h to afford *E,Z-*3,3-diarylpropenenitriles **2a–o** as products of the regioselective hydroarylation of the acetylene bond (Scheme 1). These reactions proceeded only with an excess of AlBr_3_ (6 equiv). Thus, running the reaction of nitrile **1a** with benzene under the action of less amount AlBr_3_ (1–4 equiv) resulted in an incomplete conversion of the starting compound **1a** and a low yield of the target product **2a** (<10%).

The yields of the compounds **2** were moderate (20–64%) that may be caused by possible transformations of the nitrile group in the electrophilic medium leading to oligomeric material [22]. The reaction proceeded rather regioselectively giving mainly *Z*-isomers of nitriles **2**, as products of an *anti*-addition of hydrogen and the aryl group to the carbon–carbon triple bond. Only in three cases, mixtures of *E,Z-*isomers (**2b**,**l**,**n)** in a ratio of ≈1:1 were obtained. The *E,Z-*configuration of compounds **2a–o** was determined by NOESY correlations between the vinyl proton and the aromatic protons or methyl groups in neighboring aryl substituents (see Supporting Information File 1). However, the configuration of nitrile **2o** was unclear. Benzene, *o-*, *m-*, *p*-xylenes, and 1,2,4-trimethylbenzene (mesitylene) were included in the hydroarylation of nitriles **1**. Reactions of nitriles **1a–c** with *o-*xylene led to the formation of regioisomers derived from the electrophilic substitution at different positions of this arene. Thus, nitrile **1a** gave two types of regioisomers **2n** and **2o**. After reactions of nitriles **1b**,**c** with *o-*xylene, compounds **2l** and **2m** correspondingly were obtained; other regioisomers were not isolated in amounts that were high enough for their identification.

This transformation was also tested with another strong Lewis acid, aluminum chloride (AlCl_3_), for the reaction of nitrile **1a** with benzene. However, in this case, mainly oligomeric material was obtained and the yield of the target reaction product **2a** was lower (20%) than in the same reaction with AlBr_3_. This result revealed the less effectiveness of AlCl_3_ for the hydroarylation of nitriles **1**.

**Scheme 2:**
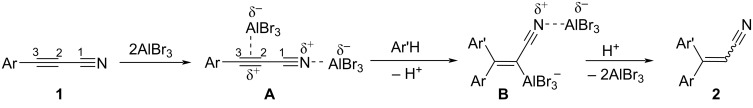
Plausible mechanism for reaction of acetylene nitriles **1** with arenes leading to nitriles **2**.

One may propose the following reaction mechanism (Scheme 2). The coordination of AlBr_3_ to both the nitrile and acetylene bonds of the starting compound **1** furnishes the highly electrophilic species **A** bearing a positive charge on the acetylenic carbon atom C3. The subsequent reaction of species **A** with the arene molecule via electrophilic aromatic substitution results in the formation of species **B**. The most probably, this stage proceeds stereoselectively due to spatial factors, with the incoming arene Ar′H attacking the acetylene bond in an *anti*-position to the bulky AlBr_3_, that determines the final predominant formation of the mainly *anti*-hydroarylation products of the starting nitriles **1**. At the last step of the reaction, a proton substitutes AlBr_3_, and final hydrolysis of the reaction mixture gives rise to nitriles **2**.

It should be noted that this AlBr_3_-promoted hydroarylation of acetylene nitriles **1** (Scheme 1) is a novel transition-metal (Pd, Pt, Rh, etc.)-free stereoselective way for the synthesis of compounds **2**. These compounds can be alternatively obtained by a Pd-catalyzed Heck reaction of 3-arylpropenenitriles with iodoarenes [23] or by a Cu-catalyzed hydroarylation of 3-arylpropynenitriles with arylboronic acid [24–25]. There is one example of use of dicyanoacetylene in a similar AlCl_3_-catalyzed hydroarylation of an acetylene bond in the synthesis of cyclophanes [26].

**Scheme 3:**
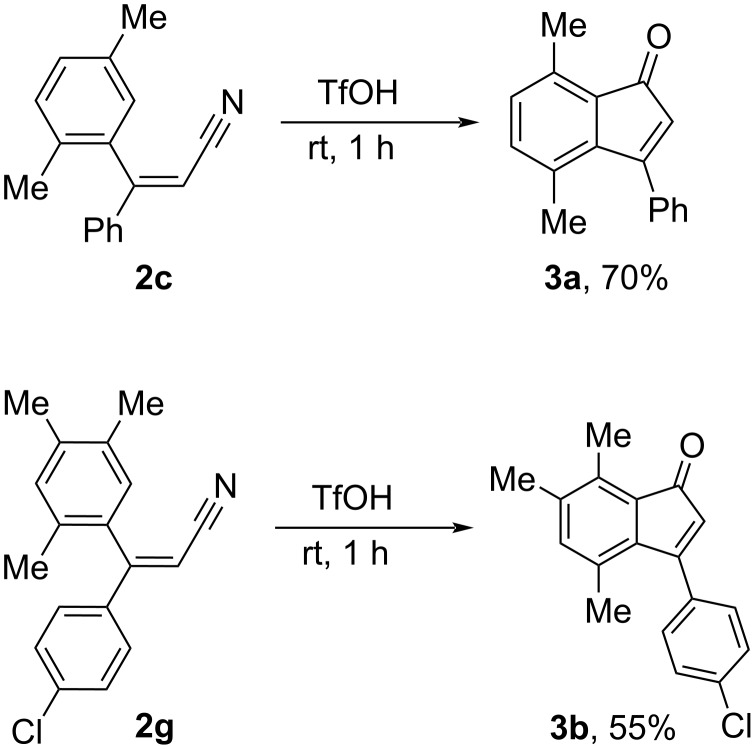
Cyclization of nitriles **2c**,**g** into indanones **3a**,**b** in TfOH.

Additionally, the cyclization of two selected nitriles **2c**,**g** into 3-arylindanones **3a**,**b** correspondingly was carried out in triflic acid TfOH (CF_3_SO_3_H) at room temperature for 1 h (Scheme 3). The intramolecular aromatic substitution by the electrophilically activated nitrile group took place in the more electron-rich methylated aryl ring. A similar cyclization of 3,3-diarylpropanenitriles into 3-arylindanones in TfOH was described by us previously [17]. It should be specially emphasized that the synthesis of indenones is an important goal in organic chemistry since this structural motif is associated with interesting chemical and biological properties [27–32].

**Scheme 4:**
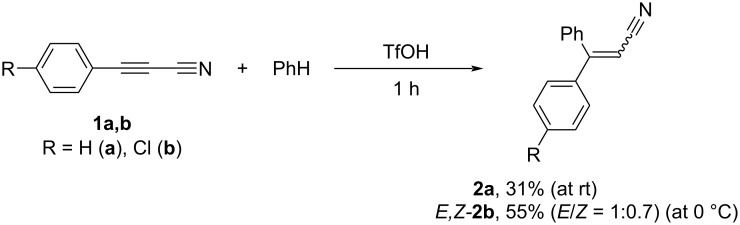
Hydrophenylation of nitriles **1a**,**b** by benzene in TfOH leading to nitriles **2a**,**b**.

We also conducted reactions of nitriles **1a–c** with arenes in TfOH at room temperature, which, however, led to complex mixtures of reaction products. Only in the cases of the reaction of nitriles **1a**,**b** with benzene in TfOH, the hydrophenylation products, i.e., nitriles **2a**,**b**, were obtained (Scheme 4; compare with synthesis of **2a**,**b** in Scheme 1).

In general, the comparison of reactions of 3-arylpropenenitriles (cinnamonitriles; ArCH=CHCN) (see our work [17]) and 3-arylpropynenitriles **1** (this work) under electrophilic activation reveals unambiguously that the electrophilic intermediates generated from acetylene nitriles **1** possess higher reactivity. Thus, the hydroarylation of 3-arylpropynenitriles **1** promoted by AlBr_3_ proceeds at room temperature (Scheme 1), whereas the same reaction of 3-arylpropenenitriles needs elevated temperature up to 80 °C [17]. Reactions of acetylene nitriles **1** with arenes in TfOH have complex character, contrary to 3-arylpropenenitriles, which react smoothly with arenes in TfOH at room temperature [17].

## Conclusion

We have developed a novel transition-metal (Pd, Pt, Rh, etc.)-free procedure for the regio- and stereoselective hydroarylation of the carbon–carbon triple bond in 3-arylpropynenitriles by arenes under electrophilic activation by aluminum bromide AlBr_3_. The obtained 3,3-diarylpropenenitriles were cyclized into 3-arylindenones in triflic acid CF_3_SO_3_H (TfOH).

## Supporting Information

File 1 Experimental procedures, compound characterization, and ^1^H and ^13^C NMR spectra of compounds.
