# Supplementary material for: AlBr3-Promoted stereoselective anti-hydroarylation of the acetylene bond in 3-arylpropynenitriles by electron-rich arenes: synthesis of 3,3-diarylpropenenitriles
Source: Beilstein J Org Chem. 2021 Nov 1;17:2663–7. doi: 10.3762/bjoc.17.180 (PMC8576820; doi:10.3762/bjoc.17.180)
Supplement: File 1 — Experimental procedures, compound characterization, and 1H and 13C NMR spectra of compounds. [file Beilstein_J_Org_Chem-17-2663-s001.pdf]

BEILSTEIN JOURNAL OF ORGANIC CHEMISTRY

Supporting Information

for

$AlBr_3$-Promoted stereoselective *anti*-hydroarylation of the acetylene bond in 3-arylpropynenitriles by electron-rich arenes: synthesis of 3,3-diarylpropenenitriles

Yelizaveta Gorbunova, Dmitry S. Ryabukhin and Aleksander V. Vasilyev

Beilstein J. Org. Chem. **2021,** *17,* 2663–2667. doi:10.3762/bjoc.17.180

Experimental procedures, compound characterization, and ^{1}H and ^{13}C NMR spectra of compounds

License and Terms: This is a supporting information file under the terms of the Creative Commons Attribution License (https://creativecommons.org/licenses/by/4.0). Please note that the reuse, redistribution and reproduction in particular requires that the author(s) and source are credited and that individual graphics may be subject to special legal provisions.
The license is subject to the *Beilstein Journal of Organic Chemistry* terms and conditions: (https://www.beilstein-journals.org/bjoc/terms)

Table of contents

1. General remarks S1
2. Synthesis and characterization of starting compounds, 3-arylpropynenitriles **1a**–**c** S1
3. Synthesis and characterization of *E*,*Z*-3,3-diarylpropenenitriles **2a**–**o** and
3-arylindenones **3a**, **b** S2
4. References S9
5. ^{1}H and ^{13}C NMR spectra, DEPT-135, COSY, NOESY spectra
of compounds **1a**–**c**, **2a**–**o**, **3a**, **b** S11

1. General remarks. NMR spectra of solutions of compounds in $CDCl_3$ were recorded on a Bruker AVANCE III 400 spectrometer at 25 °C (at 400 and 100 MHz for ^{1}H and ^{13}C NMR spectra, respectively). The residual proton solvent peak $CDCl_3$ (δ 7.26 ppm) for ^{1}H NMR spectra and the carbon signal of $CDCl_3$ (δ 77.0 ppm) for ^{13}C NMR spectra were used as references. HRMS was carried out using a Bruker maXis HRMS-ESI-QTOF instruments. Chromato-mass-spectrometry was performed with a Shimadzu QP-2010 Ultra with a SPB-1 SULFUR capillary column (30 m × 0.32 mm), thickness of the stationary phase 1.25 μm. The preparative reactions were monitored by thin-layer chromatography carried out on silica gel plates (Alugram SIL G/UV-254), using UV light for detection. Preparative TLC was performed on silica gel Chemapol L 5/40 with petroleum ether eluation. IR spectra were registered for solutions of compounds in $CHCl_3$ or for KBr disks on a Bruker IR spectrometer.

2. Synthesis and characterization of starting compounds 3-arylpropynenitriles 1a–c.

Synthesis of 3-arylpropynenitriles **1a–c** was carried out from the corresponding 3-arylpropynoic acids or their potassium salts, which were converted into acyl chlorides and then to amides; dehydration of the latter gave the target compounds (Scheme S1).

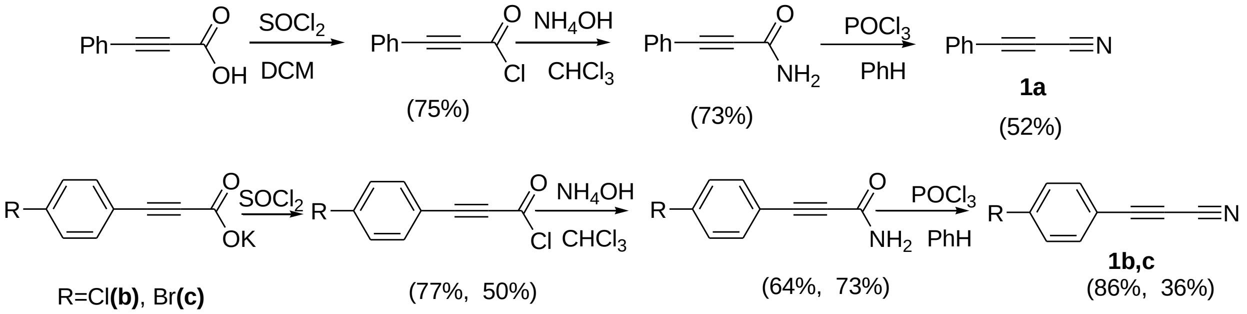

Scheme S1. Synthesis of 3-arylpropynenitriles **1a–c**.

General procedure for dehydration of 3-arylpropynamides. Synthesis of nitriles 1a–c. Phosphorus oxychloride ($POCl_3$, 4 mL) was added to a solution of 3-arylpropynamide (6 mol) in 60 mL of benzene. The reaction mixture was refluxed for 4 h. Then, it was diluted with 100 mL of dichloromethane, 100 mL of an aqueous saturated aqueous solution of $NaHCO_3$ were added, and this heterogeneous mixture was magnetically stirred at room temperature for 3–5 hours. The organic layer was separated, washed with water (100 mL), and dried with Na_2SO_4. The solvent was removed under reduced pressure. The nitriles **1b**, **c** were isolated by flash chromatography on silica gel, using a mixture of hexane/ethyl acetate 10:1 (v/v). Nitrile **1a** was used without additional purification.

3-Phenylpropynenitrile 1a [1], yield of 52%, orange oil:

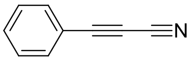

^{1}H NMR (400 MHz, $CDCl_3$): δ 7.39-7.43 (m, Har, 2H,), 7.52-7.56 (m, Har, 1H), 7.60-7.62 (m, Har, 2H);

^{13}C NMR (100 MHz, $CDCl_3$): δ 63.2(C), 83.1(C), 105.6(C≡N), 117.7 (Car), 129.0(CarH), 132.0(CarH), 133.6(CarH).

3-(4-Chlorophenyl)propynenitrile 1b[1], yield of 86%, yellow solid, m.p. 88-91°C:

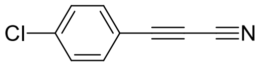

^{1}H NMR (400 MHz, $CDCl_3$): δ 7.39-7.41(m, Har, 2H,), 7.53-7.56 (m, Har, 2H);

^{13}C NMR (100 MHz, $CDCl_3$): δ 64.1(C), 81.8(C), 105.4(C≡N), 116.1(Car), 129.5(CarH), 134.8(CarH), 138.7(Car).

3-(4-Bromophenyl)propynenitrile 1c [1], yield of 36%, orange oil:

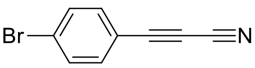

^{1}H NMR (400 MHz, $CDCl_3$): δ 7.42-7.44 (m, Har 2H), 7.51-7.53 (m, Har. 2H,);

^{13}C NMR (100 MHz, $CDCl_3$): δ 64.0(C), 81.8(C), 105.2(C≡N), 116.2(Car), 126.9(Car), 132.2(CarH), 134.6(CarH).

3. Synthesis and characterization of *E*,*Z*-3,3-diarylpropenenitriles 2a–o and 3-arylindenones 3a, b.

General procedure for the reaction of 3-arylpropynenitriles 1 with arenes under the action of $AlBr_3$. Synthesis of 3,3-diarylpropenenitriles 2. 3-Arylpropynenitrile **1** (0.64 mmol) was added to a mixture of $AlBr_3$ (1.07 g, 4 mmol, 6 equiv) with arene (2.5 mL). The reaction mixture was magnetically stirred at room temperature for 2 h. Then, the reaction was quenched with water (100 mL). The reaction products were extracted into EtOAc (3 × 100 mL), the combined extracts were washed with water (2 × 60 mL), saturated aqueous solution of $NaHCO_3$ (100 mL), water again (2 × 60 mL), and dried with Na_2SO_4. After evaporation of the solvent under reduced pressure, the obtained residue was subjected to thin-layer chromatography on silica gel using mixture of petroleum ether/ethyl acetate 10:1 (v/v) or a mixture of petroleum ether/dichloromethane/diethyl ether 20:5:1 (v/v/v). Yields of the products were determined after chromatographic isolation.

General procedure for the reaction of 3-arylpropynenitrile 1 with benzene in TfOH. Synthesis of 3,3-diarylpropenenitriles 2. 3-Arylpropynenitrile **1** (0.78 mmol) was added to a mixture of benzene (1 mL) in TfOH (2 mL). The reaction mixture was magnetically stirred at 0 °C or at room temperature for 1 h. Then, the reaction was quenched with water (100 mL). The reaction products were extracted into EtOAc (3 × 100 mL), the combined extracts were washed with water (2 × 60 mL), saturated aqueous solution of $NaHCO_3$ (100 mL), water again (2 × 60 mL), and dried with Na_2SO_4. After evaporation of the solvent under reduced pressure, the obtained residue was subjected to thin-layer chromatography on silica gel using a mixture of petroleum ether/ethyl acetate 10:1 (v/vol). Yields of the products were determined after chromatographic isolation.

General procedure for the cyclization of 3,3-diarylpropenenitriles 3 in TfOH. Synthesis of 3-arylindenones 3a, b. Solution of 3,3-diarylpropenenitrile **2** (0.4 mmol) in TfOH (2 mL) was magnetically stirred at room temperature for 1 h. Then the reaction was quenched with water (100 mL) and the reaction products were extracted into EtOAc (3 × 100 mL), the combined extracts were washed with water (2 × 60 mL), saturated aqueous solution of $NaHCO_3$ (100 mL), water again (2 × 60 mL), and dried with Na_2SO_4. After evaporation of the solvent under reduced pressure, the obtained residue was subjected to thin-layer chromatography on silica gel using a mixture of petroleum ether/ethyl acetate 10:1 (v/v). Yields of the products were determined after chromatographic isolation.

3,3-Diphenylprop-2-enenitrile 2a [2], obtained from nitrile **1a** and benzene with $AlBr_3$ in yield of 34%, or from nitrile **1a** and benzene in TfOH in yield of 31%, slightly yellow oil:

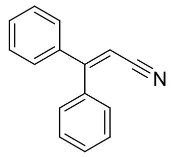

^{1}H NMR (400 MHz, $CDCl_3$): δ 5.74 (s, 1H, -CH=), 7.29-7.31 (m, 2H, Har), 7.38 (t, 2H, Har, *J* = *8.0* Hz), 7.43-7.47 (m, 6H, Har);

^{13}C NMR (100 MHz, $CDCl_3$): δ 95.0(-CH=), 118.0(C≡N), 128.6(CarH), 128.7(CarH), 128.8(CarH), 129.7(CarH), 130.1(CarH), 130.5(CarH), 137.2(Car), 139.0(Car), 163.3 (C);

IR (KBr), ν, cm^{-1}: 2213 (C≡N);

HRMS, *m/z*: calcd. $C_{15}H_{11}N^+$ $[M+Na]^+$ 228.0784, found 228.0789.

(*E*-) and (*Z*)-3-(4-Chlorophenyl)-3-(phenyl)- prop-2-enenitrile 2b [2] obtained from nitrile **1b** and benzene with $AlBr_3$ in yield of 25%, or from nitrile **1b** and benzene in TfOH in yield of 55%, slightly yellow oil:

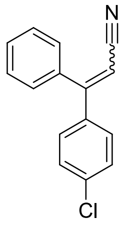

^{1}H NMR (400 MHz, $CDCl_3$): mixture of (E-) and (Z)-isomers, δ 5.72 (s, 1H, -CH=), 5.74 (s, 1H, -CH=), 7.24 (d, 2H, Har, *J* =*8.8* Hz), 7.28 (d, 2H, Har, *J* = *7.2* Hz), 7.34-7.47 (m, 14H, Har);

^{13}C NMR (100 MHz, $CDCl_3$): mixture of (E-) and (Z)-isomers, δ 95.4 (2-CH=), 117.7(2 C≡N), 128.5(CarH), 128.8(CarH), 128.9(CarH), 129.0(CarH), 129.1(CarH), 129.6(CarH), 129.9(CarH), 130.4(CarH), 130.8(CarH), 131.1(CarH), 135.6(Car), 136.4(Car), 136.7(Car), 136.8(Car), 137.5(Car), 138.6(Car), 162.0 (2C);

HRMS, *m/z*: calcd. $C_{15}H_{10}ClNNa^+$ $[M+Na]^+$ 262.0394, found 262.0394.

(*Z*)-3-(2,5-Dimethylphenyl)-3-phenylprop-2-enenitrile 2c obtained from nitrile **1a** and 1,4-dimethylbenzene with $AlBr_3$ in yield of 30%, slightly yellow oil:

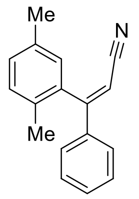

^{1}H NMR (400 MHz, $CDCl_3$): δ 2.06 (s, 3H, CH_3), 2.36 (s, 3H, CH_3), 5.96 (s, 1H, -CH=), 7.04 (s, 1H, Har), 7.17 (s, 2H, Har), 7.29 (d, 2H, Har, *J* = *6.8* Hz), 7.36 (t, 2H, Har, *J* = *6.8* Hz), 7.39-7.43 (m, 1H, Har);

^{13}C NMR (100 MHz, $CDCl_3$): δ 19.2(CH_3), 21.0(CH_3), 96.2(-CH=), 117.2(C≡N), 127.3(CarH), 129.0(CarH), 129.7(CarH), 130.2(CarH), 130.6(CarH), 130.7(CarH), 132.7(Car), 135.7(Car), 136.6(Car), 137.6(Car), 136.8(Car), 163.3(C);

IR (KBr), ν, cm^{-1}: 2215 (C≡N);

HRMS, *m/z*: calcd. $C_{17}H_{15}NNa^+$ $[M+Na]^+$ 256.1097, found 256.1102

(*Z*)-3-(2,5-Dimethylphenyl)-3-(4-chlorophenyl)prop-2-enenitrile 2d obtained from nitrile **1b** and 1,4-dimethylbenzene with $AlBr_3$ in yield of 35%, slightly yellow oil:

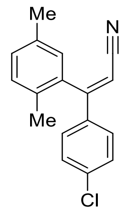

^{1}H NMR (400 MHz, $CDCl_3$): δ 2.04 (s, 3H, CH_3), 2.36 (s, 3H, CH_3), 5.94 (s, 1H, -CH=), 7.01 (s, 1H, Har), 7.17 (s, 2H, Har), 7.21 (d, 2H, Har, *J* = *8.6* Hz), 7.32 (d, 2H, Har, *J* = *8.6* Hz);

^{13}C NMR (100 MHz, $CDCl_3$): δ: 19.2(CH_3), 21.0(CH_3), 96.6(-CH=), 117.2(C≡N), 128.5(CarH), 129.3(CarH), 129.7(CarH), 130.4(CarH), 130.9(CarH), 132.7(Car), 135.9(Car), 136.1(Car), 136.2(Car), 136.8(Car), 162.0(C);

IR (KBr), ν, cm^{-1}: 2216 (C≡N);

HRMS, *m/z*: calcd. $C_{17}H_{14}ClNNa^+$ $[M+Na]^+$ 290.0707, found 290.0704.

(*Z*)-3-(4-Bromophenyl)-3-(2,5-dimethylphenyl)prop-2-enenitrile 2e obtained from nitrile **1c** and 1,4-dimethylbenzene with $AlBr_3$ in yield of 40%, slightly yellow oil:

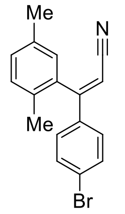

^{1}H NMR (400 MHz, $CDCl_3$): δ 2.04 (s, 3H, CH_3), 2.35 (s, 3H, CH_3), 5.94 (s, 1H, -CH=), 7.00 (s, 1H, Har), 7.14 (d, 2H, Har, *J* = *8.6* Hz), 7.16 (s, 2H, Har), 7.48 (d, 2H, Har, *J* = *8.6* Hz);

^{13}C NMR (100 MHz, $CDCl_3$): δ 19.2(CH_3), 21.0(CH_3), 96.7(-CH=), 117.2(C≡N), 125.2(Car), 128.8(CarH), 129.7(CarH), 130.5(CarH), 130.9(CarH), 132.3(CarH), 132.7(Car), 135.9(Car), 136.1(Car), 136.5(Car), 162.1(C);

IR (KBr), ν, cm^{-1}: 2216 (C≡N);

HRMS, *m/z*: calcd. $C_{17}H_{14}BrNNa^+$ $[M+Na]^+$ 334.0202, found 334.0192.

(*Z*)-3-(2,4,5-Trimethylphenyl)-3-phenylprop-2-enenitrile 2f obtained from nitrile **1a** and 1,2,4-trimethylbenzene with $AlBr_3$ in yield of 26%, slightly yellow oil:

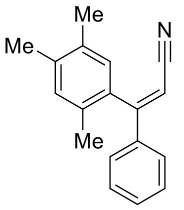

^{1}H NMR (400 MHz, $CDCl_3$): δ 2.03 (s, 3H, CH_3), 2.26 (s, 3H, CH_3), 2.28 (s, 3H, CH_3), 5.93 (s, 1H, -CH=), 6.98 (s, 1H, Har), 7.05 (s, 1H, Har), 7.28-7.40 (m, 5H, Har);

^{13}C NMR (100 MHz, $CDCl_3$): δ 19.2(CH_3), 19.3(CH_3), 19.7(CH_3), 96.1(-CH=), 117.8(C≡N), 127.3(CarH), 128.9(CarH), 130.3(CarH), 130.5(CarH), 132.2(CarH), 133.1(Car), 134.2(Car), 134.3(Car), 137.9(Car), 138.0(Car), 163.4(C);

IR (KBr), ν, cm^{-1}: 2214 (C≡N);

HRMS, *m/z*: calcd. $C_{18}H_{17}NNa^+$ $[M+Na]^+$ 270.1253, found 270.1256.

(*Z*)-3-(4-Chlorophenyl)-3-(2,4,5-trimethylphenyl)prop-2-enenitrile 2g obtained from nitrile **1b** and 1,2,4-trimethylbenzene with $AlBr_3$ in yield of 38%, slightly yellow oil:

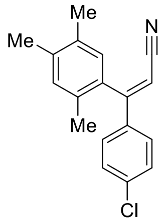

^{1}H NMR (400 MHz, $CDCl_3$): δ 2.01 (s, 3H, CH_3), 2.26 (s, 3H, CH_3), 2.28 (s, 3H, CH_3), 5.90 (s, 1H, -CH=), 6.96 (s, 1H, Har), 7.05,(s, 1H, Har), 7.22 (d, 2H, Har, *J = 8.4* Hz), 7.32 (d, 2H, Har, *J = 8.8* Hz);

^{13}C NMR (100 MHz, $CDCl_3$): δ 19.2(CH_3), 19.3(CH_3), 19.7(CH_3), 96.5(-CH=), 117.4(C≡N), 128.6(CarH), 129.2(CarH), 130.3(CarH), 132.3(CarH), 133.0(Car), 133.7(Car), 134.4(Car), 136.4(Car), 136.7(Car), 138.2(Car), 162.1(C);

HRMS, *m/z*: calcd. $C_{18}H_{16}ClNNa^+$ $[M+Na]^+$ 304.0863, found 304.0864.

(*Z*)-3-(4-Bromophenyl)-3-(2,4,5-trimethylphenyl)prop-2-enenitrile 2h obtained from nitrile **1c** and 1,2,4-trimethylbenzene with $AlBr_3$ in yield of 25%, slightly yellow oil:

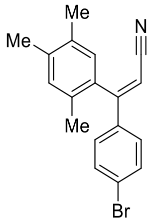

^{1}H NMR (400 MHz, $CDCl_3$): δ 2.01 (s, 3H, CH_3), 2.25 (s, 3H, CH_3), 2.28 (s, 3H, CH_3), 5.91 (s, 1H, -CH=), 6.95 (s, 1H, Har), 7.04 (s, 1H, Har), 7.15 (d, 2H, Har, *J = 8.4* Hz), 7.47 (d, 2H, Har, *J = 8.4* Hz);

^{13}C NMR (100 MHz, $CDCl_3$): δ 19.2(CH_3), 19.4(CH_3), 19.7(CH_3), 96.6(-CH=), 117.4(C≡N), 125.1(Car), 128.8(CarH), 130.3(CarH), 132.2(CarH), 132.3(CarH), 133.1(Car), 133.6(Car), 134.5(Car), 136.9(Car), 138.2(Car), 162.2(C);

IR (KBr), ν, cm^{-1}: 2215 (C≡N);

HRMS, *m/z*: calcd. $C_{18}H_{16}BrNNa^+$ $[M+Na]^+$ 348.0358, found 348.0359.

(*Z*)-3-(2,4-Dimethylphenyl)-3-phenylprop-2-enenitrile 2i obtained from nitrile **1a** and 1,3-dimethylbenzene with $AlBr_3$ in yield of 42%; slightly yellow oil:

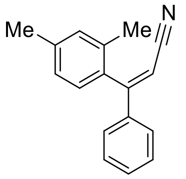

^{1}H NMR (400 MHz, $CDCl_3$): δ 2.04 (s, 3H, CH_3), 2.37 (s, 3H, CH_3), 5.94 (s, 1H, -CH=), 7.09-7.12 (m, 3H, Har), 7.28d (d, 2H, Har, *J=7.2* Hz), 7.32-7.36 (m, 2H, Har), 7.38-7.42 (m, 1H, Har);

^{13}C NMR (100 MHz, $CDCl_3$): δ 19.7(CH_3), 21.4(CH_3), 96.3(-CH=), 117.7(C≡N), 126.9 (CarH), 127.3(CarH), 128.9(CarH), 129.3(CarH), 130.6(CarH), 131.7(CarH), 133.9(Car), 135.8(Car), 137.9(Car), 139.4(Car), 163.4(C);

IR (KBr), ν, cm^{-1}: 2215 (C≡N);

HRMS, *m/z*: calcd. $C_{17}H_{15}NNa^+$ $[M+Na]^+$ 256.1097, found 256.1099.

(*Z*)-3-(4-Bromophenyl)-3-(2,4-dimethylphenyl)prop-2-enenitrile 2j obtained from nitrile **1c** and 1,3-dimethylbenzene with $AlBr_3$ in yield of 33%, slightly yellow oil:

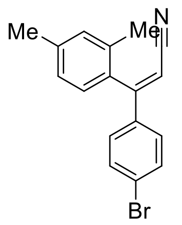

^{1}H NMR (400 MHz, $CDCl_3$): δ 2.03 (s, 3H, CH_3), 2.37 (s, 3H, CH_3), 5.93 (s, 1H, -CH=), 7.09-7.12 (m, 3H, Har), 7.14 (d, 2H, Har, *J = 8.8* Hz), 7.48 (d, 2H, Har, *J = 8.4* Hz);

^{13}C NMR (100 MHz, $CDCl_3$): δ 19.7(CH_3), 21.4(CH_3), 96.8(-CH=), 117.4(C≡N), 125.2(Car), 127.1(CarH), 128.7(CarH), 129.3(CarH), 131.8(CarH), 132.2(Car), 133.4(Car), 135.8(Car), 136.8(Car), 139.7(Car), 162.2(C);

HRMS, *m/z*: calcd. $C_{17}H_{14}BrNNa^+$ $[M+Na]^+$ 334.0202, found 334.0200.

(*Z*)-3-(4-Chlorophenyl)-3-(2,4-dimethylphenyl)prop-2-enenitrile 2k obtained from nitrile **1b** and 1,3-dimethylbenzene with $AlBr_3$ in yield of 64%, slightly yellow oil:

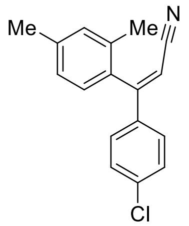

^{1}H NMR (400 MHz, $CDCl_3$): δ 2.03 (s, 3H, CH_3), 2.37 (s, 3H, CH_3), 5.92 (s, 1H, -CH=), 7.01-7.09 (m, 3H, Har), 7.21 (d, 2H, Har, *J = 8.6* Hz), 7.32 (m, 2H, Har, *J = 8.6* Hz);

^{13}C NMR (100 MHz, $CDCl_3$): δ 19.7(CH_3), 21.4(CH_3), 96.7(-CH=), 117.4(C≡N), 127.1 (CarH), 128.6(CarH), 129.2(CarH), 129.3(CarH), 131.8(CarH), 133.4(Car), 135.8(Car), 136.3(Car), 136.8(Car), 139.7(Car), 162.1(C);

IR (KBr), ν, cm^{-1}: 2216 (C≡N);

HRMS, *m/z*: calcd. $C_{17}H_{14}ClNNa^+$$[M+Na]^+$ 290.0707, found 290.0735.

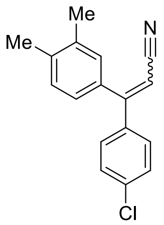

(*E*)- and (*Z*)-3-(4-Chlorophenyl)-3-(3,4-dimethylphenyl)prop-2-enenitrile 2l obtained from nitrile **1b** and 1,2-dimethylbenzene with $AlBr_3$ in yield of 20%, slightly yellow oil:

^{1}H NMR (400 MHz, $CDCl_3$): mixture of (E-) and (Z)-isomers, δ 2.25 (s, 3H, CH_3), 2.28 (c 3H, CH_3), 2.29 (s, 3H, CH_3), 2.32 (s, 3H, CH_3), 5.63 (s, 1H, -CH=), 5.70 (c 1H, -CH=), 6.98-7.00 (m, 1H, Har), 7.03 (s, 1H, Har), 7.12-7.17 (m, 3H, Har), 7.20-7.22 (m, 1H, Har), 7.24 (d, 2H, Har, *J = 7.2* Hz), 7.34 (d, 2H, Har, *J =7.2* Hz), 7.37 (d, 2H, Har, *J = 6.8* Hz), 7.42 (d, 2H, Har, *J = 7.2* Hz);

^{13}C NMR (100 MHz, $CDCl_3$): δ 19.8(CH_3), 19.9(CH_3), 94.3(-CH=), 94.6(-CH=), 118.0(2C≡N), 126.1(CarH), 127.2(CarH), 128.9(CarH), 129.0(CarH), 129.6(CarH), 129.9(CarH), 130.0(CarH), 130.1(CarH), 130.6(CarH), 131.0(CarH), 134.3(Car), 135.8(Car), 136.1(Car), 136.2(Car), 136.6(Car), 137.1(Car), 137.3(Car), 137.9(Car), 139.4(Car), 140.0(Car), 162.2(2C);

HRMS, *m/z*: calcd. $C_{17}H_{14}ClNNa^+$ $[M+Na]^+$ 290.0707, found 290.0704.

(*Z*)**-3-(4-Bromophenyl)-3-(2,3-dimethylphenyl)prop-2-enenitrile 2m** obtained from nitrile **1c** and 1,2-dimethylbenzene with $AlBr_3$ in yield of 20%, slightly yellow oil:

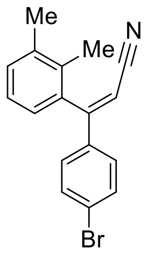

^{1}H NMR (400 MHz, $CDCl_3$): δ 1.99 (s, 3H, CH_3), 2.31 (s, 3H, CH_3), 5.97 (s, 1H, -CH=), 7.04 (d, 1H, Har, *J = 6.0* Hz), 7.14 (m, 2H, Har, *7.2* Hz), 7.20 (t, 1H, Har, *J = 6.4* Hz), 7.25 (d, 1H, Har, *J = 6.0* Hz), 7.48 (d, 2H, Har, *J = 6.8* Hz);

^{13}C NMR (100 MHz, $CDCl_3$): δ 16.7(CH_3), 20.5(CH_3), 96.8(-CH=), 117.3(C≡N), 125.2(Car), 126.1(CarH), 127.0(CarH), 128.7(CarH), 131.2(CarH), 132.3(CarH), 134.4(Car), 136.4(Car), 136.7(Car), 138.0(Car), 162.6(C);

HRMS, *m/z*: calcd. $C_{17}H_{14}BrNNa^{+}$ $[M+Na]^{+}$ 334.0202, found 334.0199.

(*E*)**- and** (*Z*)**-3-(3,4-Dimethylphenyl)-3-phenylprop-2-enenitrile 2n** [3] obtained from nitrile **1a** and 1,2-dimethylbenzene with $AlBr_3$ in yield of 25%, slightly yellow oil:

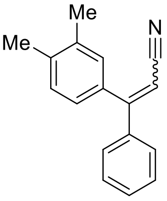

^{1}H NMR (400 MHz, $CDCl_3$): mixture of (E-) and (Z)-isomers, δ 2.25 (c 3H, CH_3), 2.28 (c 3H, CH_3), 2.29 (s, 3H, CH_3), 2.32 (s, 3H, CH_3), 5.66 (c 1H, -CH=), 5.70 (s, 1H, -CH=), 7.01 (m, 1H, Har), 7.07(s, 1H, Har), 7.13 (d, 1H, Har, *J = 8.0* Hz), 7.17-7.21 (m, 3H, Har), 7.29-7.31 (m, 2H, Har), 7.35-7.38 (m, 2H, Har), 7.42-7.46 (m, 5H, Har);

^{13}C NMR (100 MHz, $CDCl_3$): mixture of (E-) and (Z)-isomers, δ 19.8(CH_3), 19.9(3CH_3), 93.9(-CH=), 94.2(-CH=), 118.3(2C≡N), 126.2(CarH), 127.3(CarH), 128.6(CarH), 128.7(CarH), 129.6(CarH), 129.7(CarH), 129.9(CarH), 130.0(2CarH), 130.4(CarH), 130.7(CarH), 134.7(Car), 136.7(Car), 136.9(Car), 137.1(Car), 137.4(Car), 139.1(Car), 139.5(Car), 139.7(Car), 163.4(C), 163.5(C);

IR (KBr), ν, cm^{-1}: 2212 (C≡N);

HRMS, *m/z*: calcd. $C_{17}H_{15}NNa^{+}[M+Na]^{+}$ 256.1097, found 256.1097.

3-(2,3-Dimethylphenyl)-3-phenylprop-2-enenitrile 2o (E- or Z-configuration was unclear) obtained from nitrile **1a** and 1,2-dimethylbenzene with $AlBr_3$ in yield of 20%, slightly yellow oil:

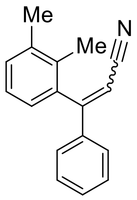

^{1}H NMR (400 MHz, $CDCl_3$): δ 2.01 (s, 3H, CH_3), 2.32 (s, 3H, CH_3), 5.98 (s, 1H, -CH=), 7.06d (d, 1H, Har, *J = 6.0* Hz), 7.19 (t, 1H, Har, *J = 6.0* Hz), 7.24 (d, 1H, Har, *J = 6.4* Hz), 7.28 (d, 2H, Har, *J = 6.0* Hz), 7.35 (t, 2H, Har, *J = 6.0* Hz), 7.38-7.41 (m, 1H, Har);

^{13}C NMR (100 MHz, CDCl$_3$): δ 16.7(CH$_3$), 20.5(CH$_3$), 96.3(-CH=), 117.6(C≡N), 125.9(CarH), 127.1(CarH), 127.3(CarH), 129.0(CarH), 130.6(CarH), 130.9(CarH), 134.4(Car), 136.9(Car), 137.8(2Car), 163.8(C);

IR (KBr), ν, cm^{-1}: 2215 (C≡N);

HRMS, *m/z*: calcd. $C_{17}H_{15}NNa^+$[M+Na]$^+$ 256.1097, found 256.1096.

4,7-Dimethyl-3-phenyl-1*H*-inden-1-one 3a obtained from nitrile **2c** в TfOH in yield of 70%, slightly yellow oil:

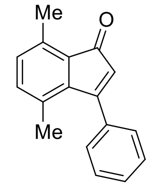

^{1}H NMR (400 MHz, CDCl$_3$): δ 1.87 s, 3H, CH$_3$), 2.53 (s, 3H, CH$_3$), 5.73 (s, 1H, -CH=), 6.92-6.96 (m, 2H, Har), 7.37-7.46 (m, 5H, Har);

^{13}C NMR (100 MHz, CDCl$_3$): δ 17.2(CH$_3$), 19.7(CH$_3$), 126.2(CarH), 127.0(CarH), 128.6(CarH), 129.0(CarH), 130.8(Car), 132.8(CarH), 135.6(Car), 136.5(Car), 136.7(CarH), 141.9(Car), 164.8(C), 198.8(C=O);

IR (KBr), ν, cm^{-1}: 1699 (C=O);

HRMS, *m/z*: calcd. $C_{17}H_{15}O^+$ [M+H]$^+$ 235.1117, found 235.1117.

3-(4-Chlorophenyl)-4,6,7-trimethyl-1*H*- inden-1-one 3b obtained from nitrile **2g** в TfOH in yield of 55%, slightly yellow oil:

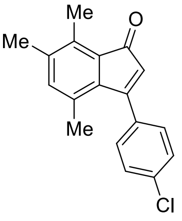

^{1}H NMR (400 MHz, CDCl$_3$): δ 1.85 (s, 3H, CH$_3$), 2.23 (s, 3H, CH$_3$), 2.49 (s, 3H, CH$_3$), 5.66 (s, 1H, -CH=), 6.83 (s, 1H, Har), 7.32 (m, 2H, Har, $J = 8.0$ Hz), 7.41 (m, 2H, Har, $J = 6.8$ Hz);

^{13}C NMR (100 MHz, CDCl$_3$): δ 12.9(CH$_3$), 19.2(CH$_3$), 19.7(CH$_3$), 126.2(CarH), 128.5(CarH), 128.7(Car), 128.9(CarH), 130.2(Car), 130.0(Car), 135.1(Car), 135.7(Car), 137.2(CarH), 139.5(Car), 140.5(Car), 163.3(C), 198.9(C=O);

IR (KBr), ν, cm^{-1}: 1694 (C=O);

HRMS, *m/z*: calcd. $C_{18}H_{15}ClNaO^+$ [M+Na]$^+$ 305.0704, found 305.0701.

4. References

[1] T. Wang, H. Yin, N. Jiao, *Adv. Synth. Catal.*, **2013**, *355*, 1207– 1210.

[2] J. Masllorens, M. Moreno-Mañas, A. Pla-Quintana, R.Pleixats, A. Roglans, *Synthesis*, **2002**, 1903-1911.

[3] L. Hao, F. Wu, Z.-C. Ding, S.-X. Xu, Y.-L. Ma, L. Chen, Z.-P. Zhan, *Chem.Eur.J.*, **2012**, *18*, 6453-6456.

5. ^{1}H and ^{13}C NMR spectra, DEPT-135, COSY, NOESY spectra of compounds 1a-c, 2a-o, 3a,b

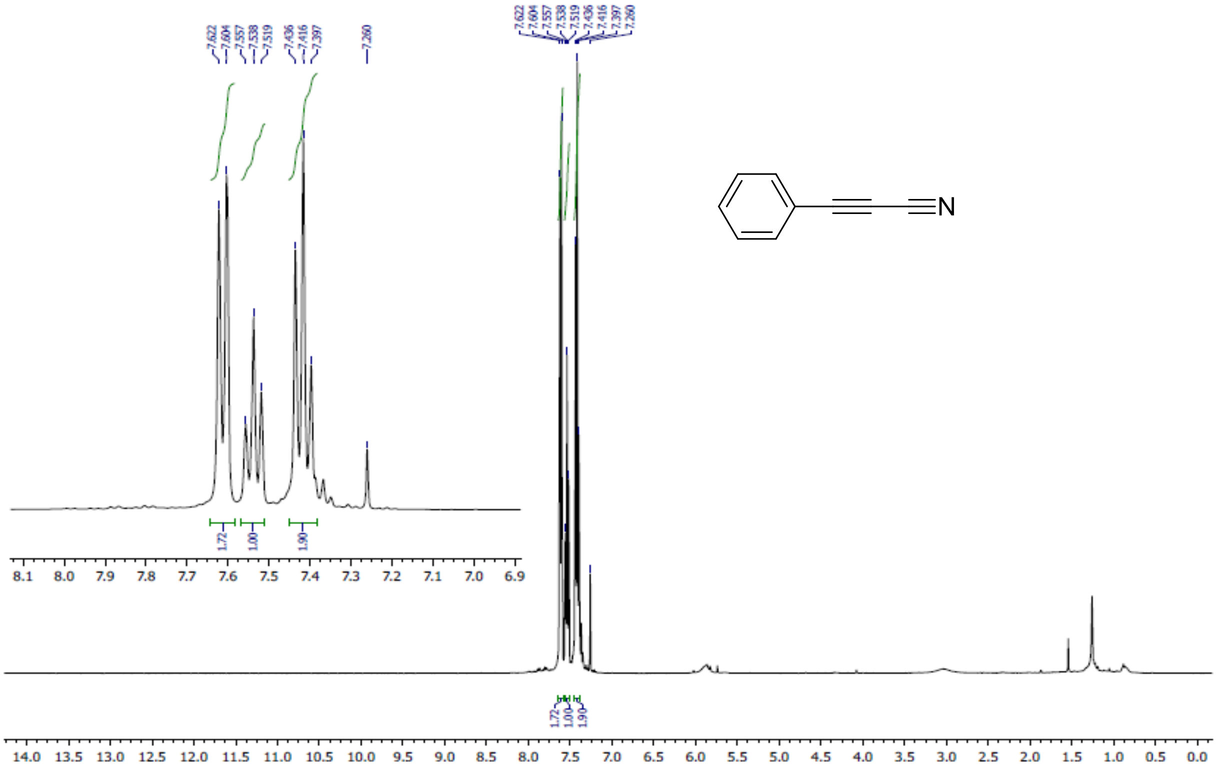

Figure S1. ^{1}H NMR spectrum of compound **1a** ($CDCl_3$, 400 MHz).

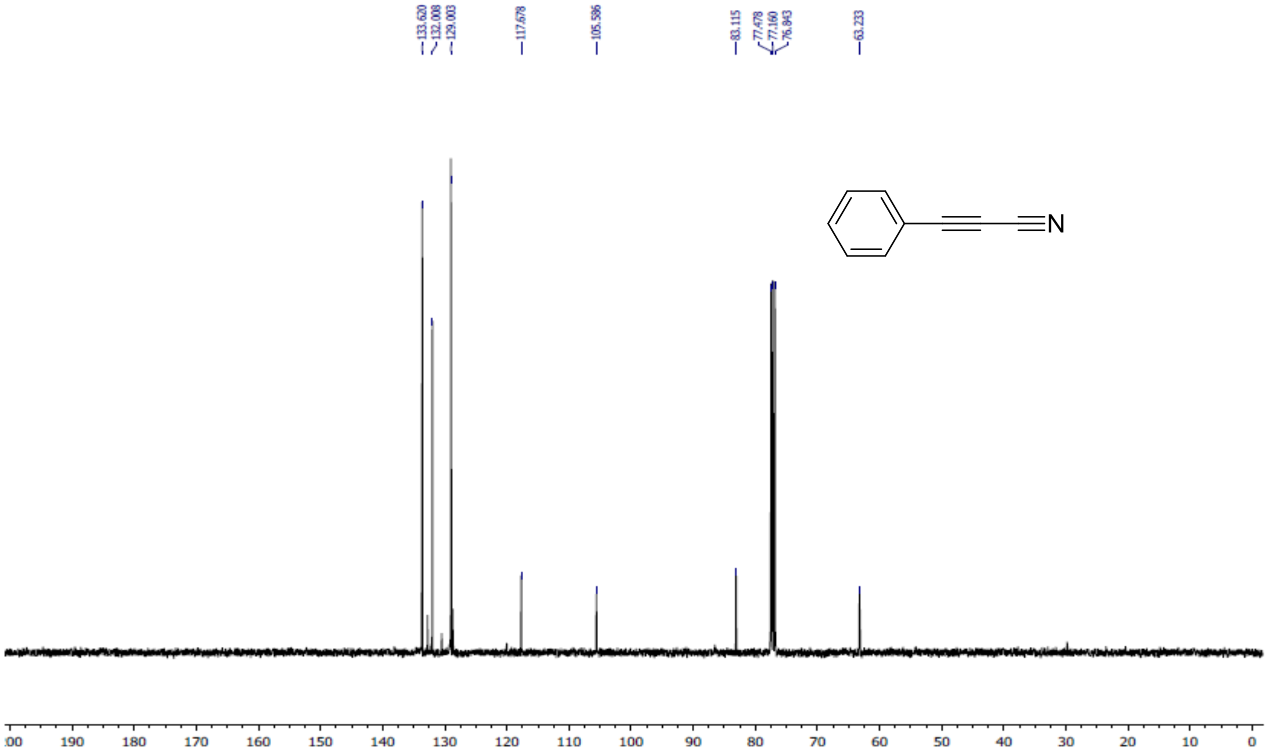

Figure S2. ^{13}C NMR spectrum of compound **1a** ($CDCl_3$, 100 MHz).

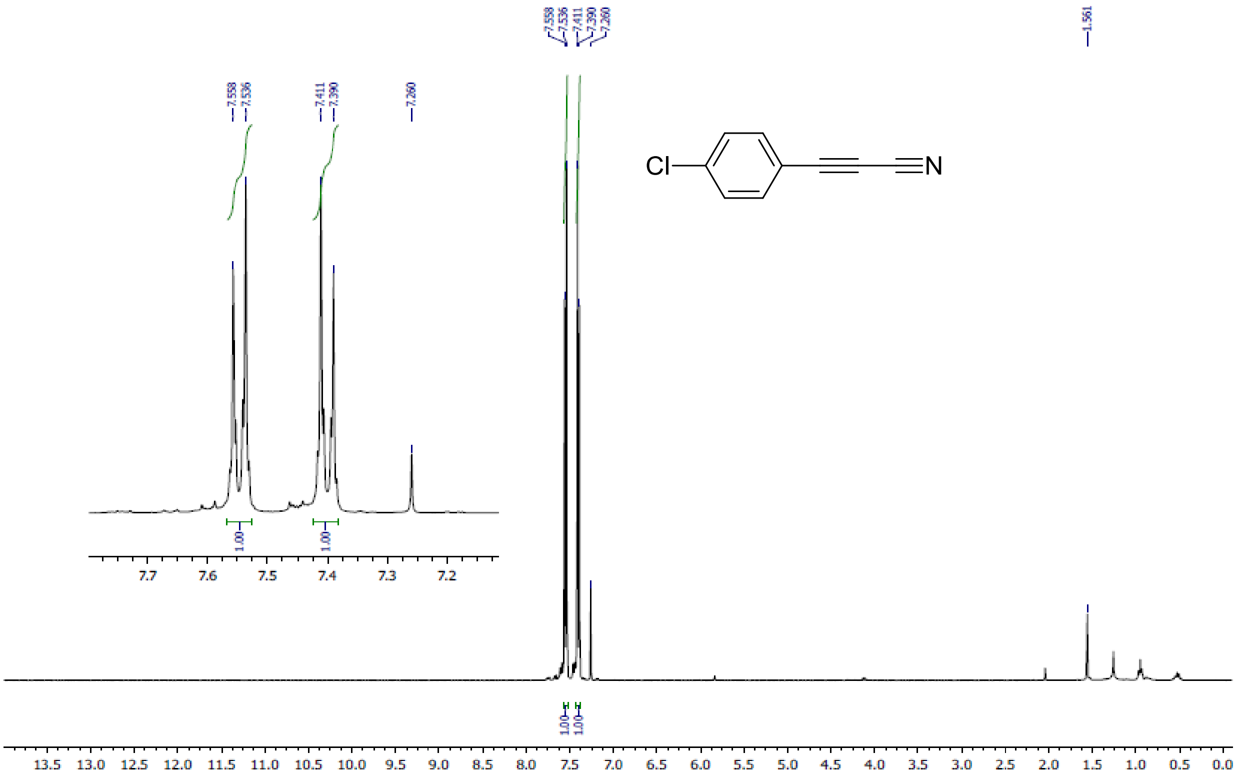

Figure S3. ^{1}H NMR spectrum of compound **1b** ($CDCl_3$, 400 MHz).

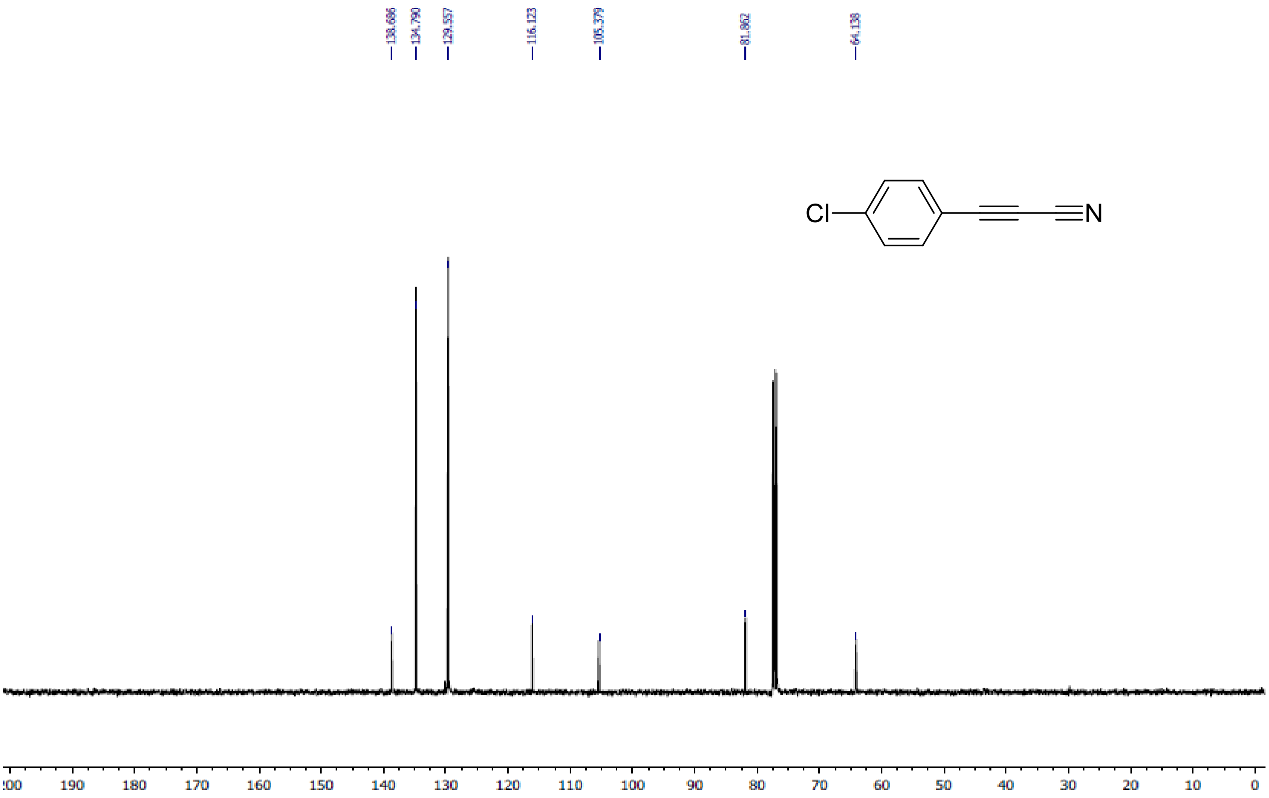

Figure S4. ^{13}C NMR spectrum of compound **1b** ($CDCl_3$, 100 MHz).

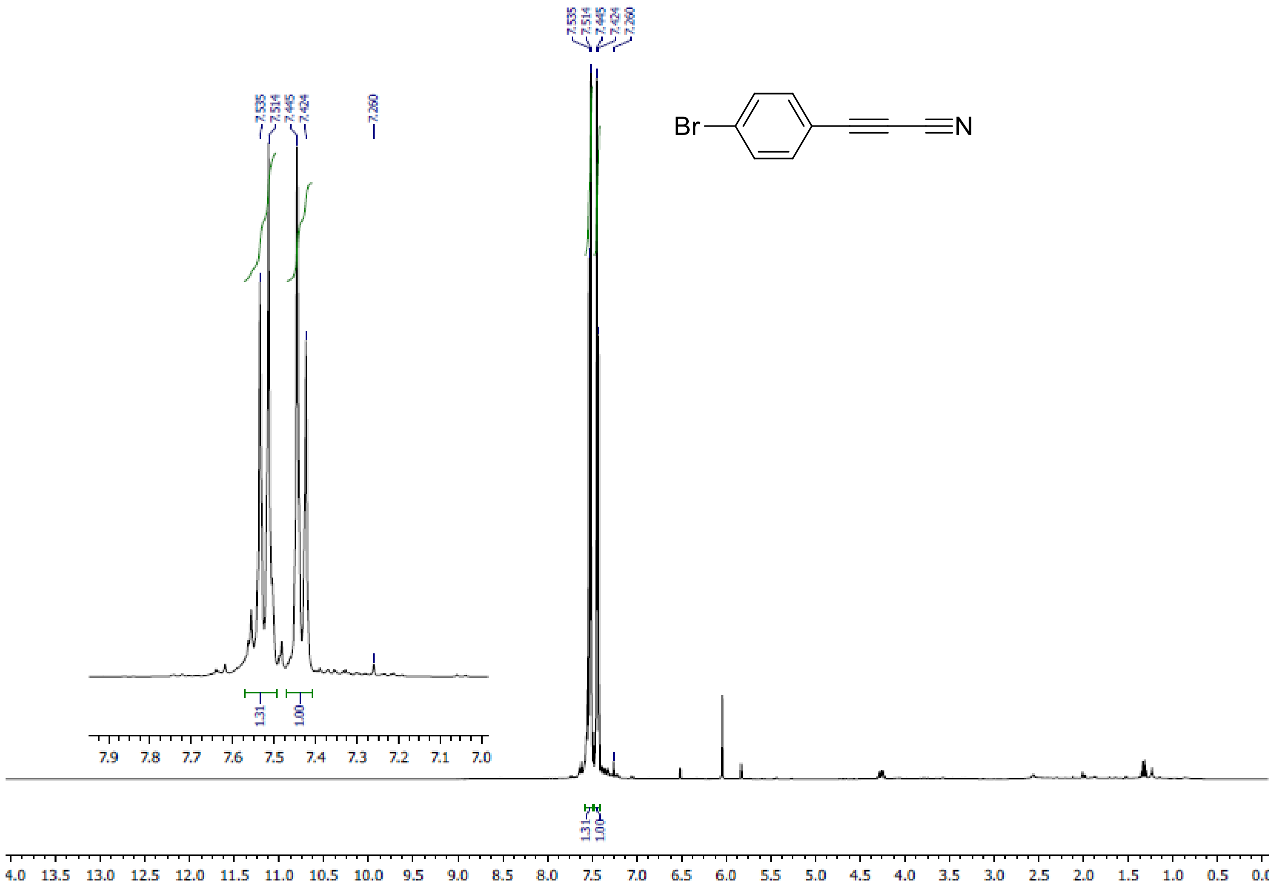

Figure S5. ^{1}H NMR spectrum of compound **1c** ($CDCl_3$, 400 MHz).

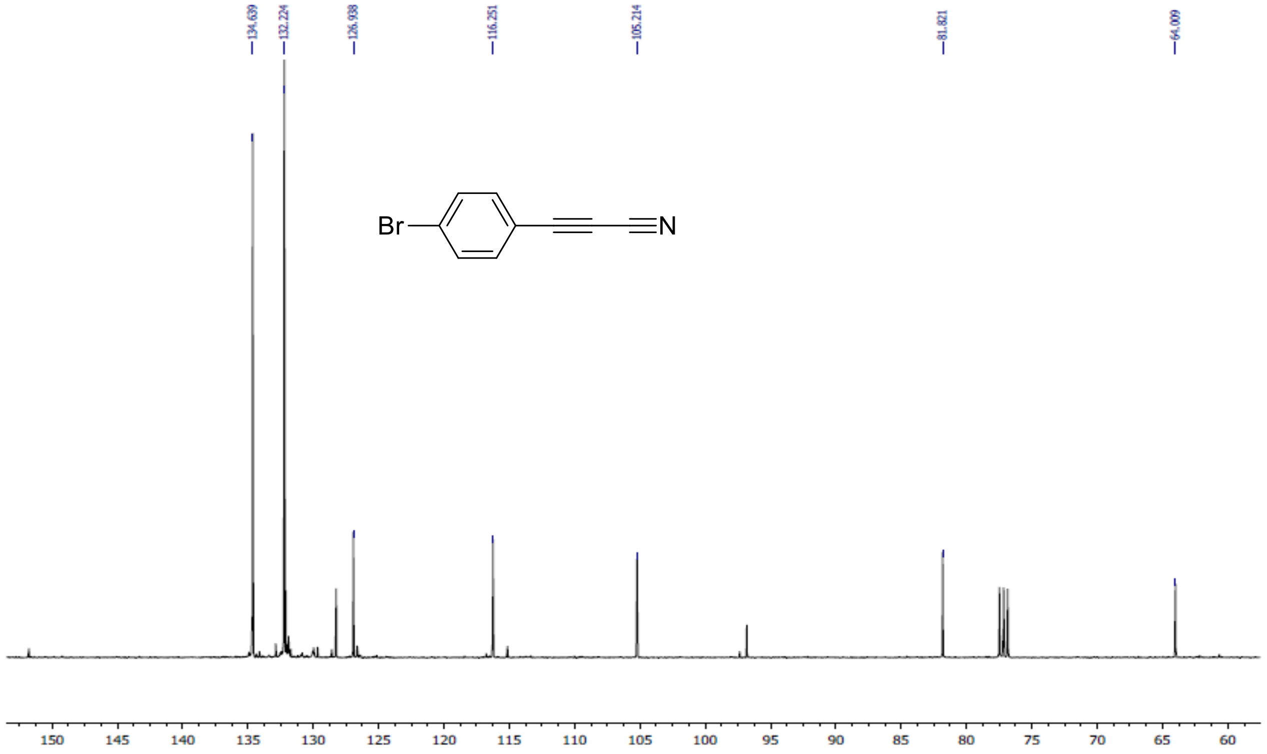

Figure S6. ^{13}C NMR spectrum of compound **1c** ($CDCl_3$, 100 MHz).

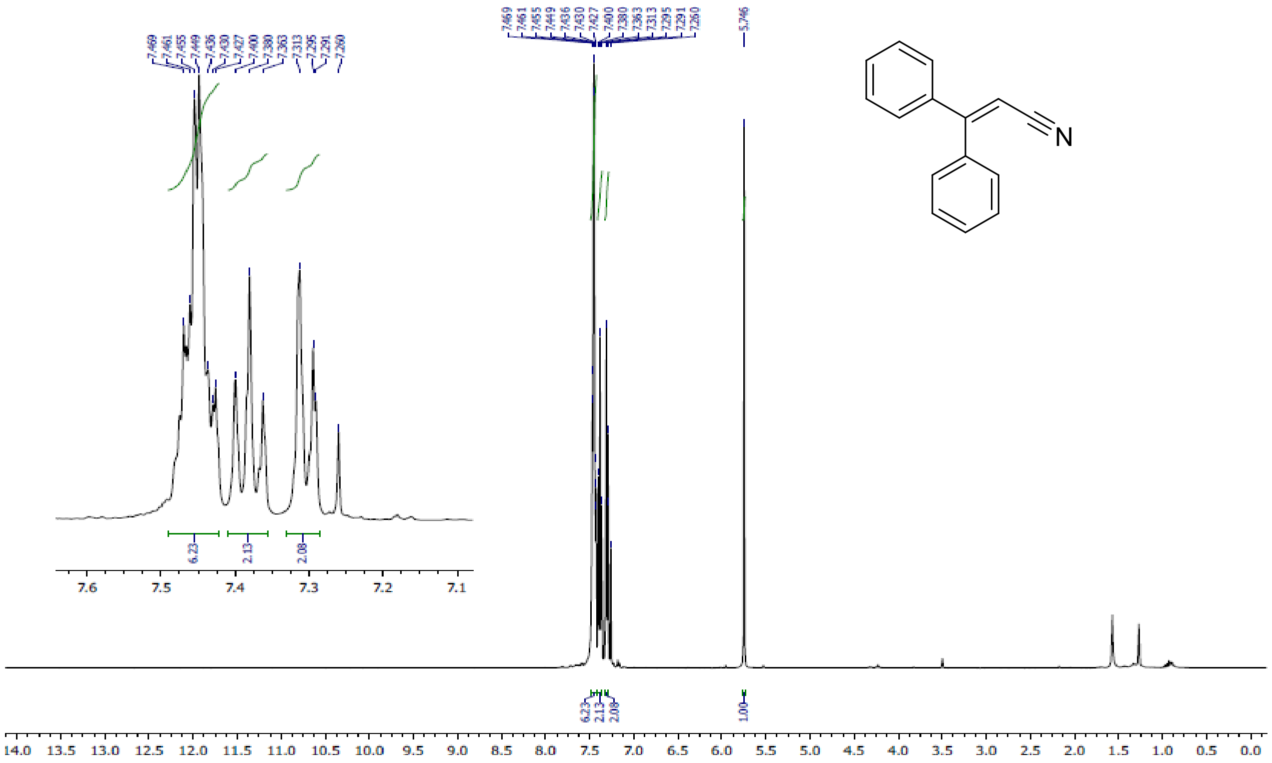

Figure S7. ^{1}H NMR spectrum of compound **2a** ($CDCl_3$, 400 MHz).

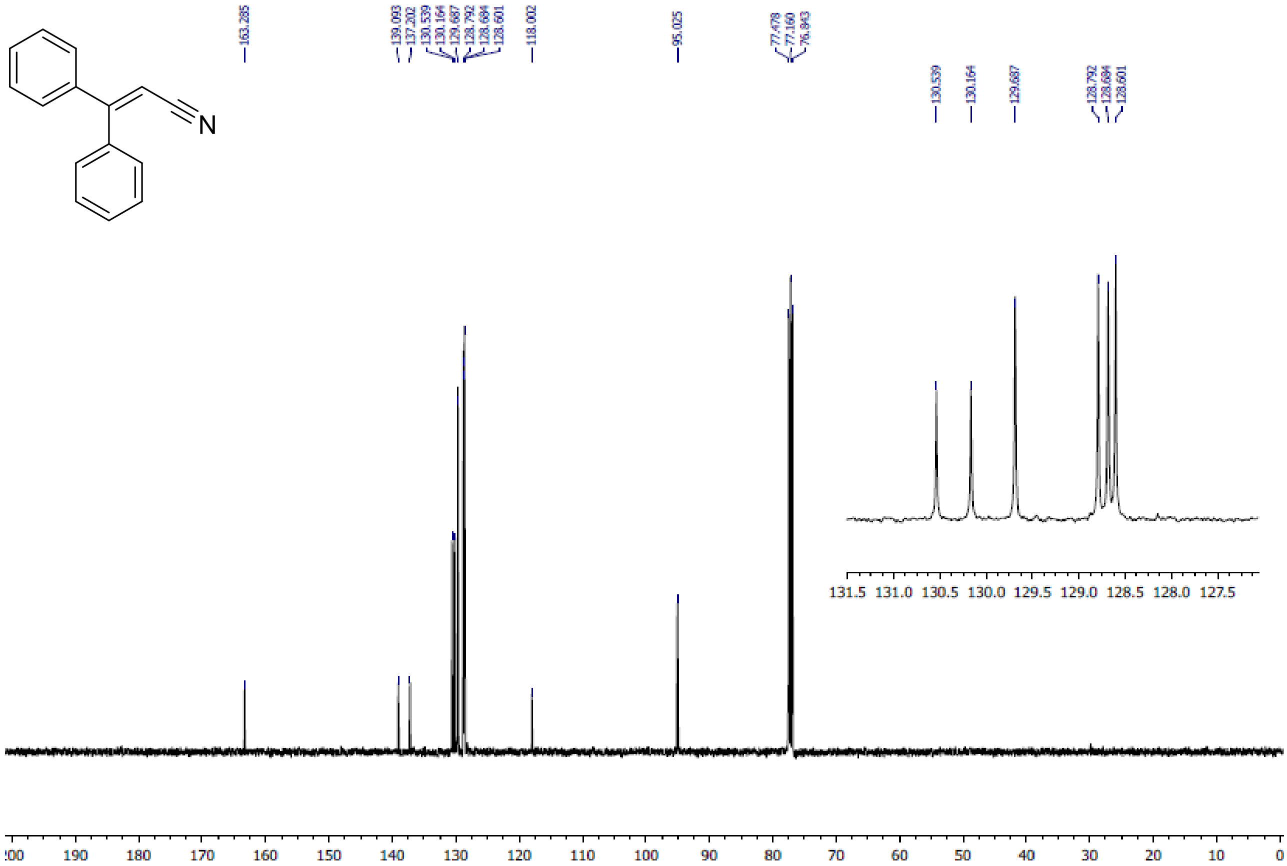

Figure S8. ^{13}C NMR spectrum of compound **2a** ($CDCl_3$, 100 MHz).

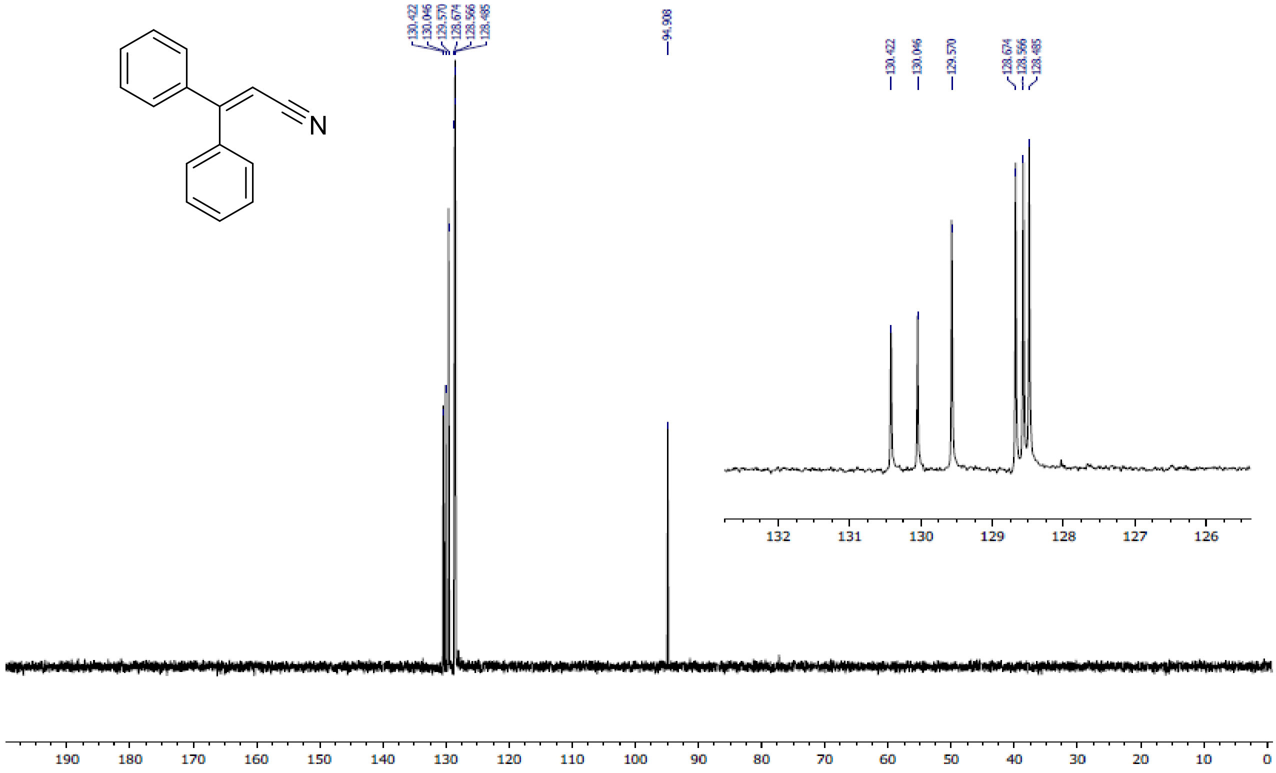

Figure S9. DEPT[135] NMR spectrum of compound **2a** ($CDCl_3$, 400 MHz).

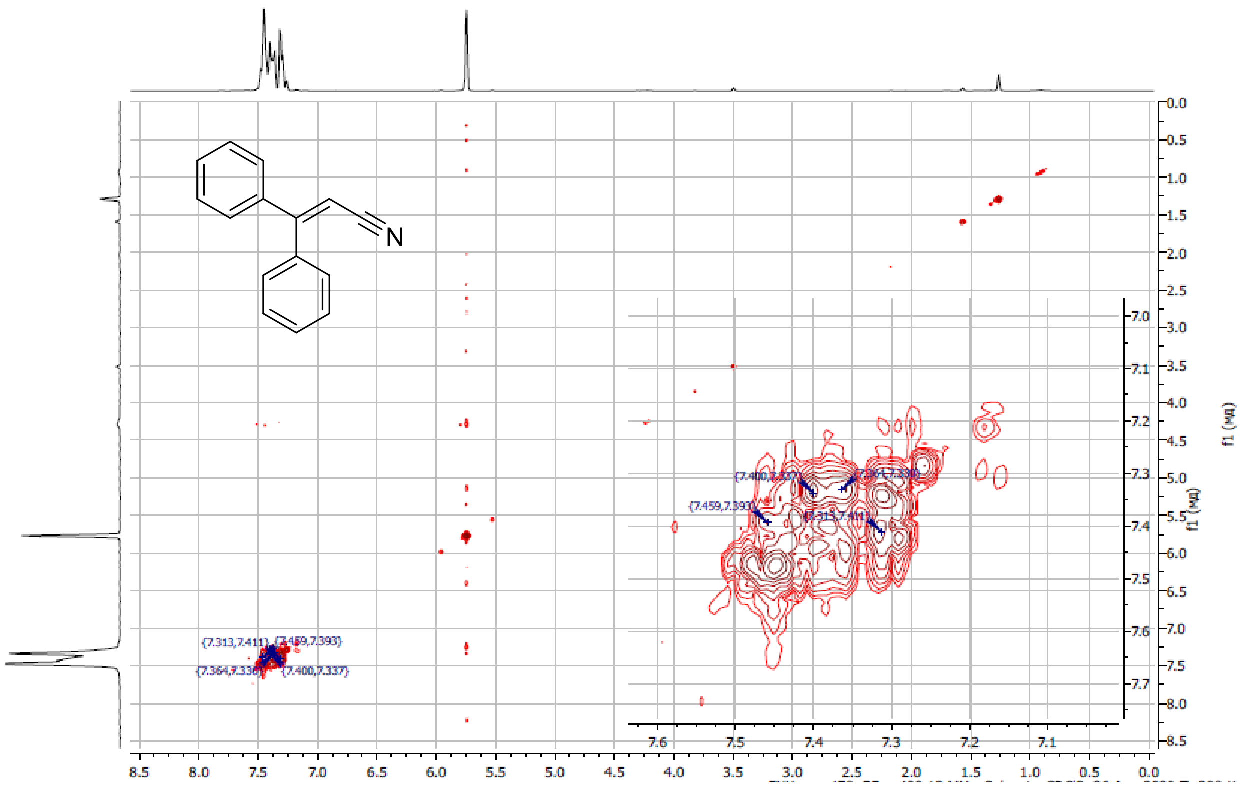

Figure S10. COSY NMR spectrum of compound **2a** ($CDCl_3$, 400 MHz).

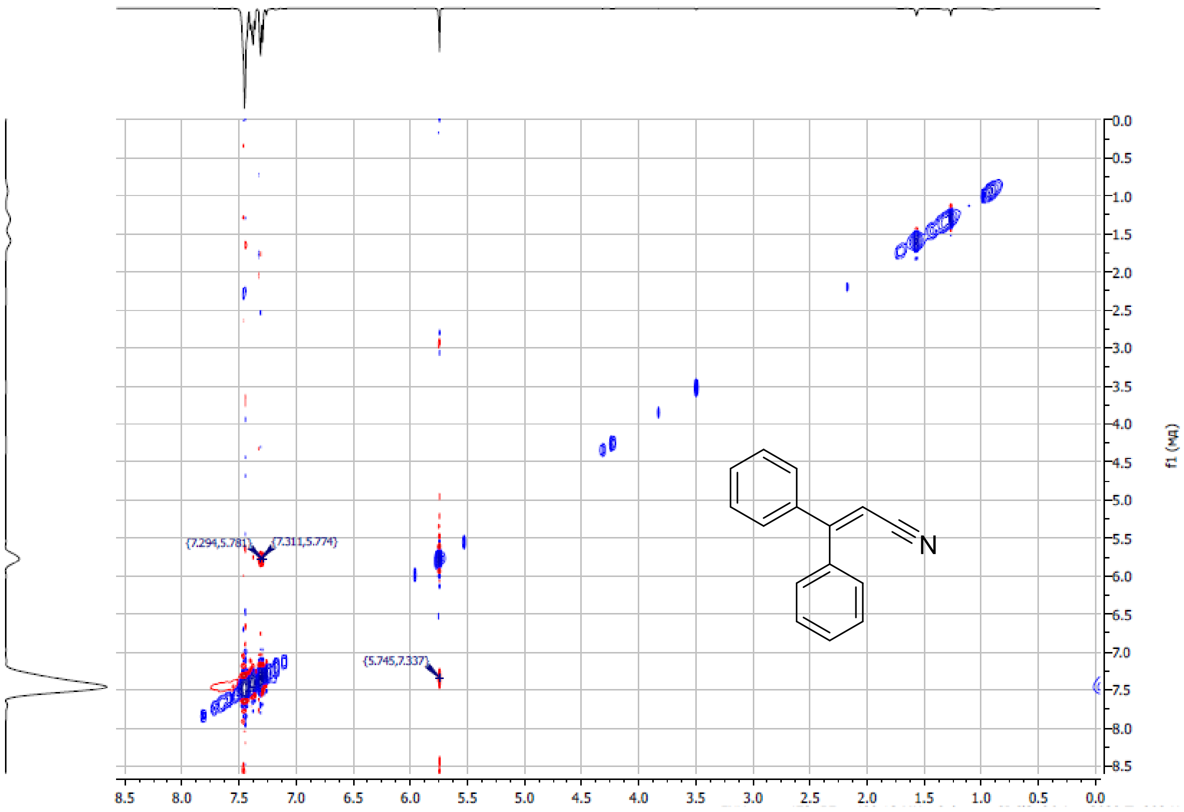

Figure S11. NOESY NMR spectrum of compound **2a** ($CDCl_3$, 400 MHz).

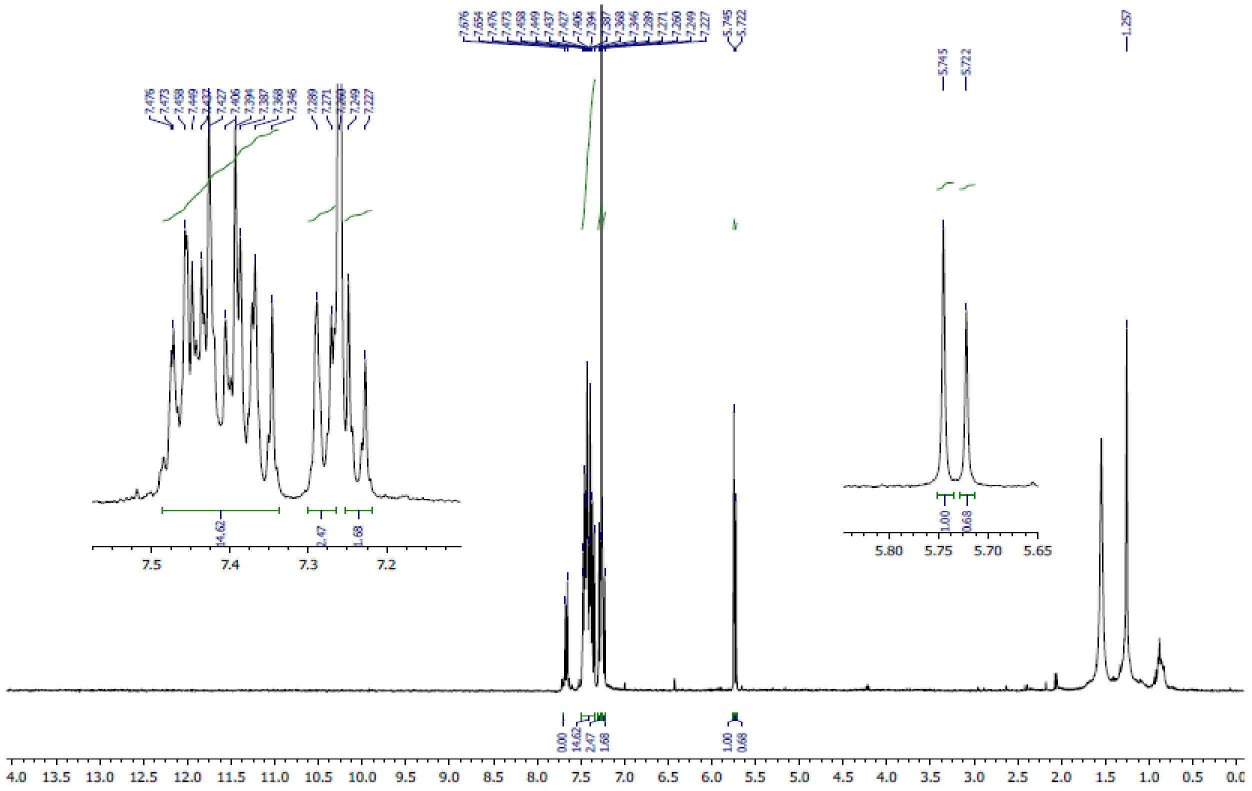

Figure S12. ^{1}H NMR spectrum of mixture of compounds *E*-,*Z*-**2b** ($CDCl_3$, 400 MHz).

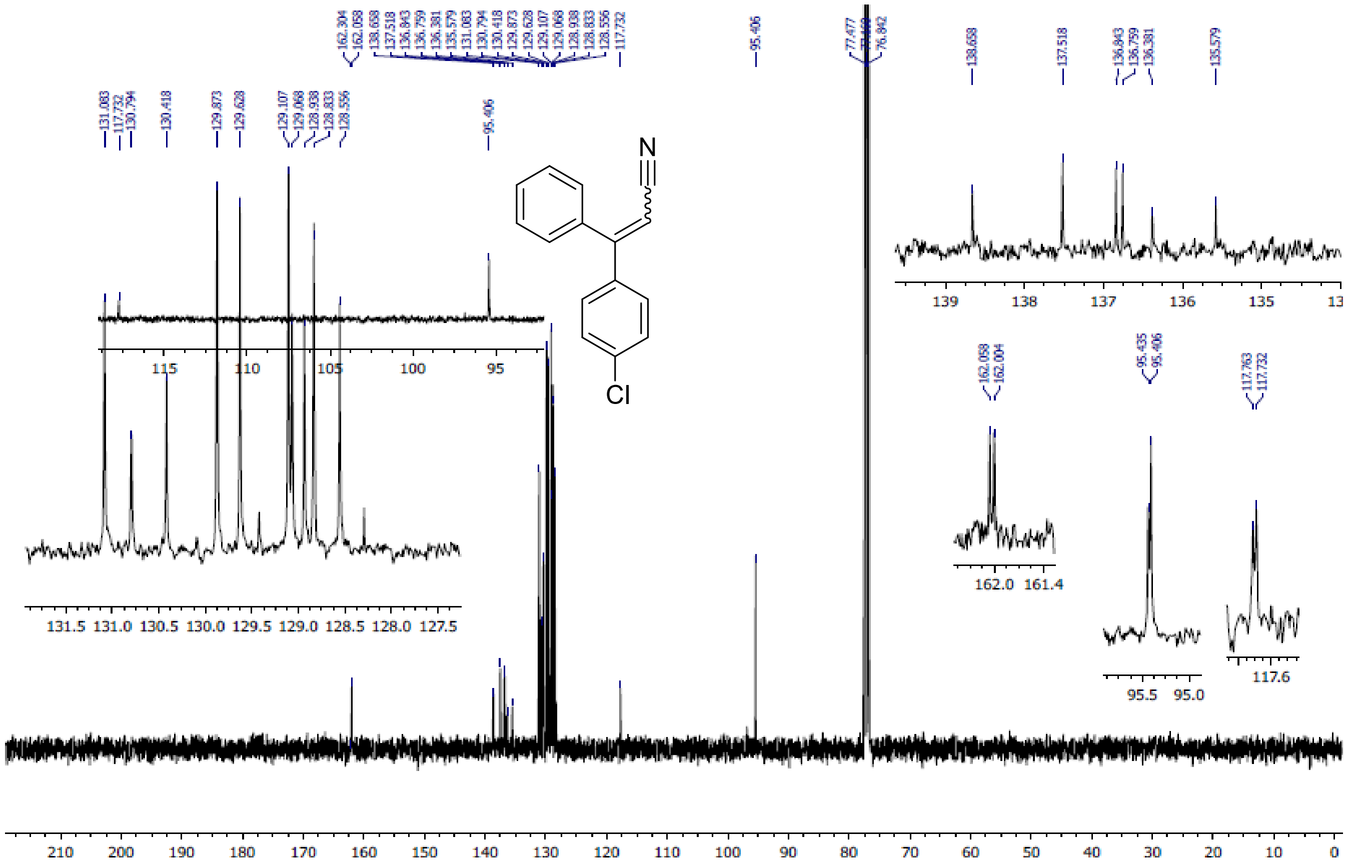

Figure S13. ^{13}C NMR spectrum of mixture of compounds *E*,*Z*-**2b** ($CDCl_3$, 100 MHz).

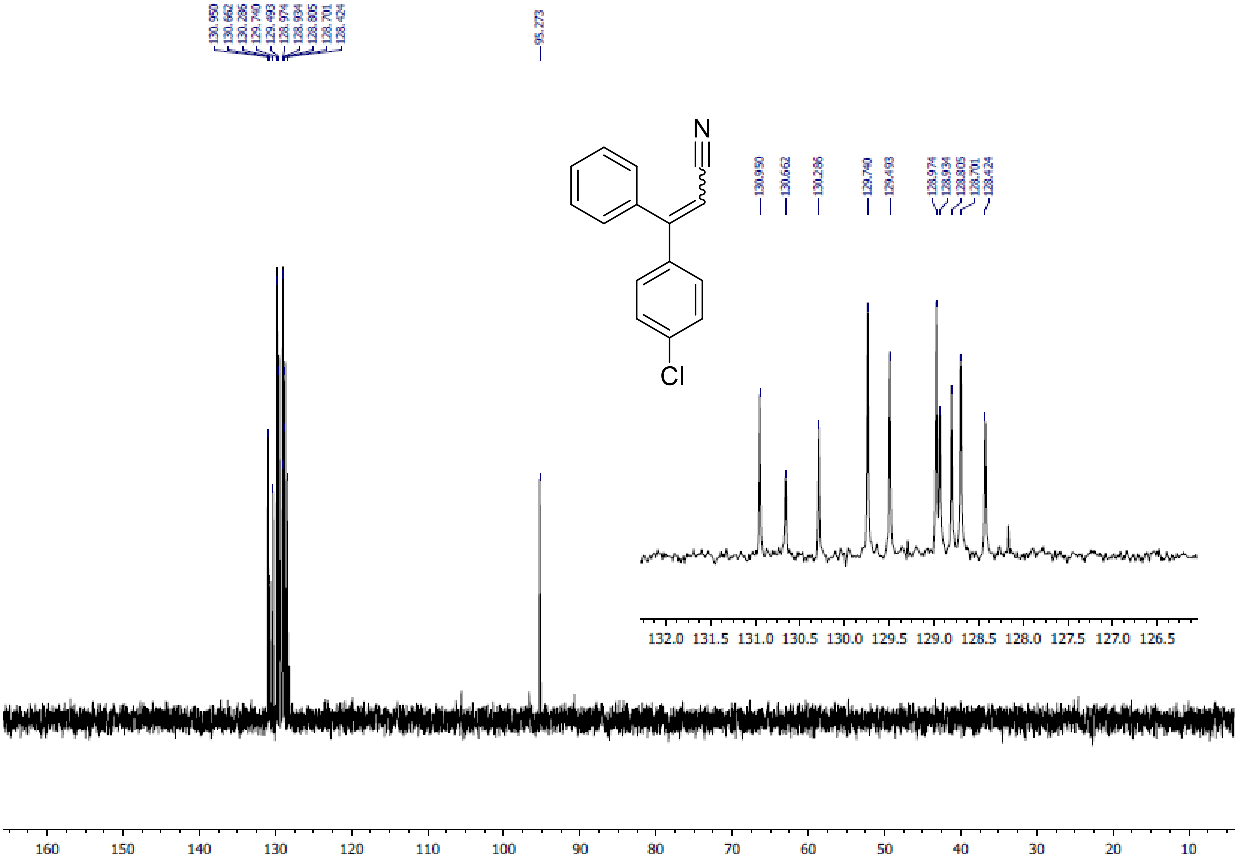

Figure S14. DEPT^{135} NMR spectrum of mixture of compounds *E*,*Z*-**2b** ($CDCl_3$, 400 MHz).

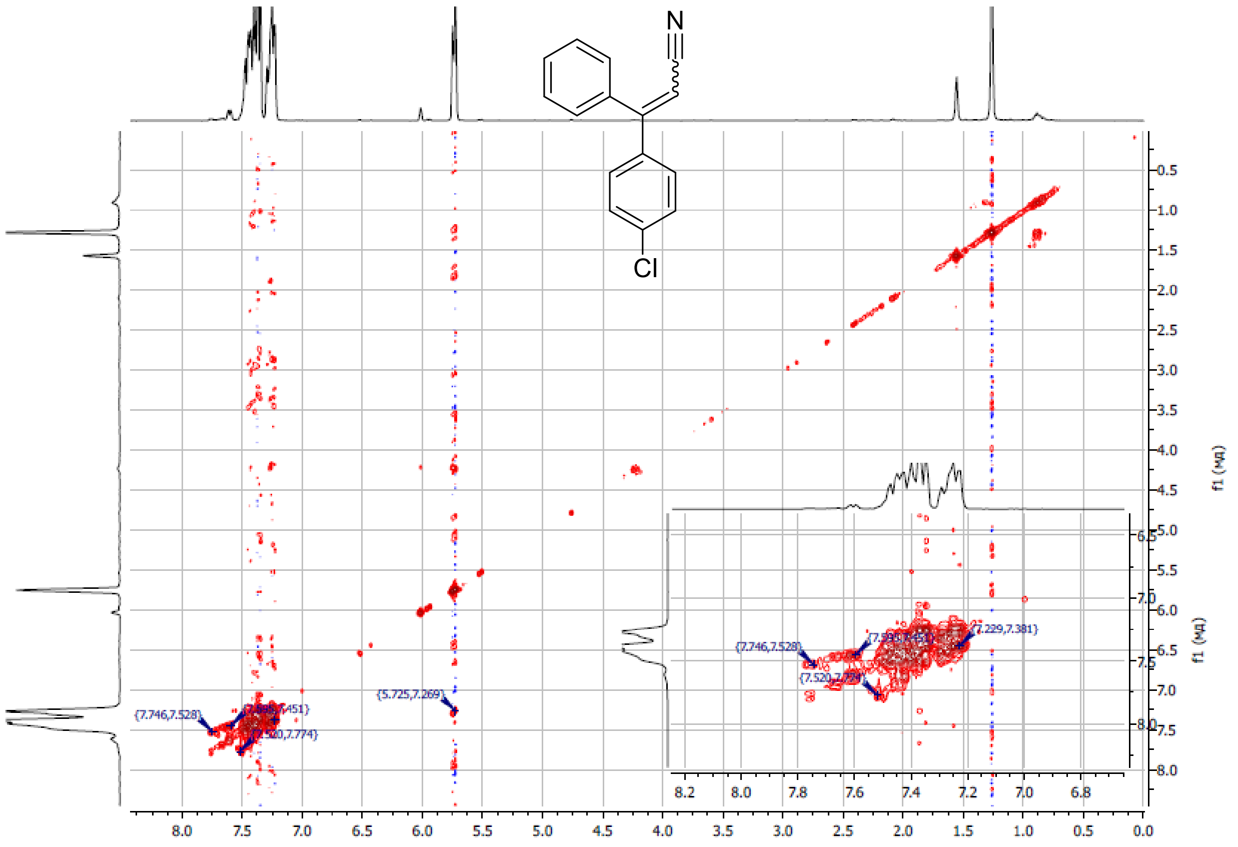

Figure S15. COSY NMR spectrum of mixture of compounds *E*,*Z*-**2b** ($CDCl_3$, 400 MHz).

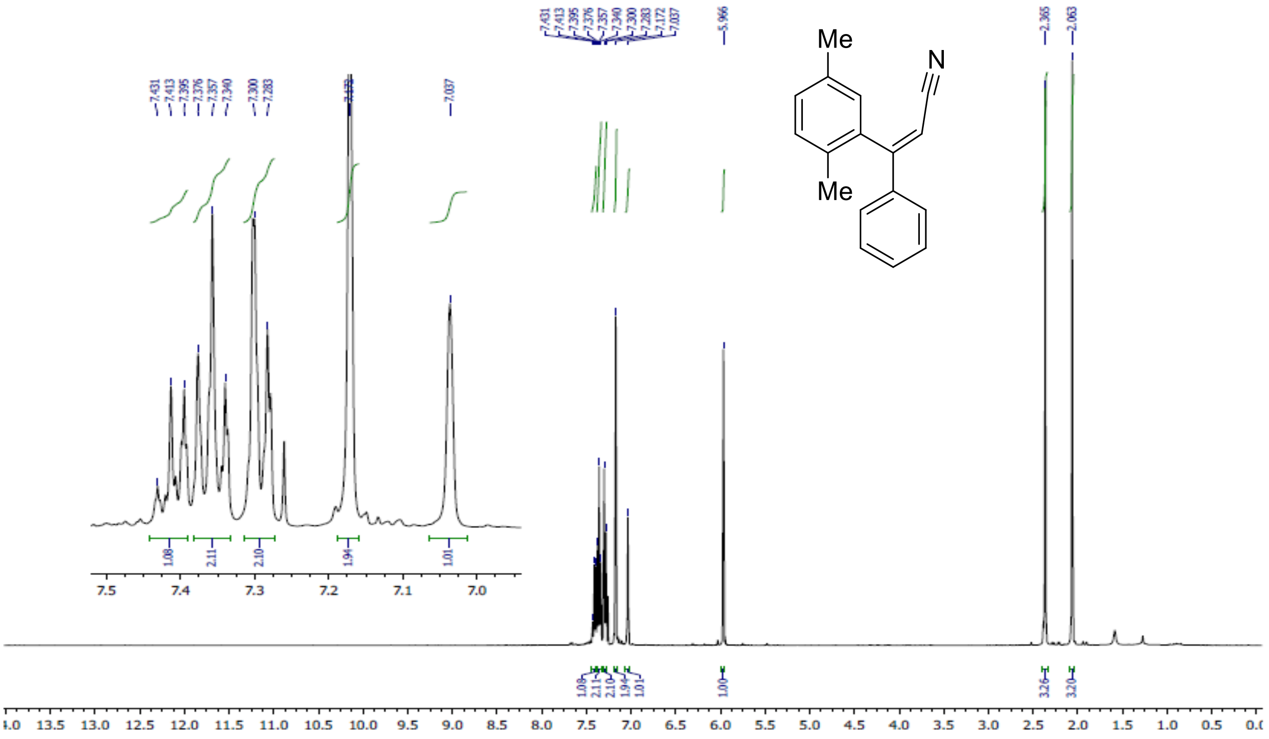

Figure S16. ^{1}H NMR spectrum of compound **2c** ($CDCl_3$, 400 MHz).

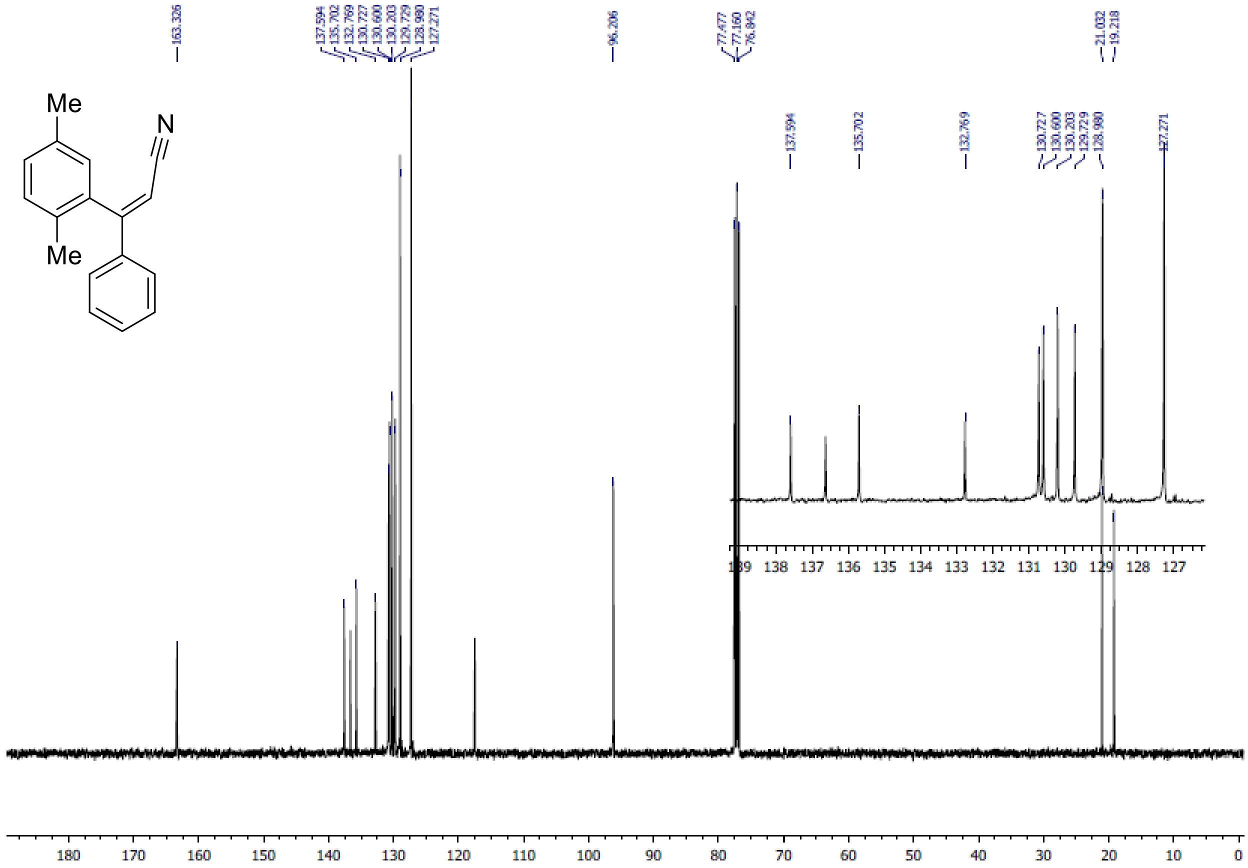

Figure S17. ^{13}C NMR spectrum of compound **2c** ($CDCl_3$, 100 MHz).

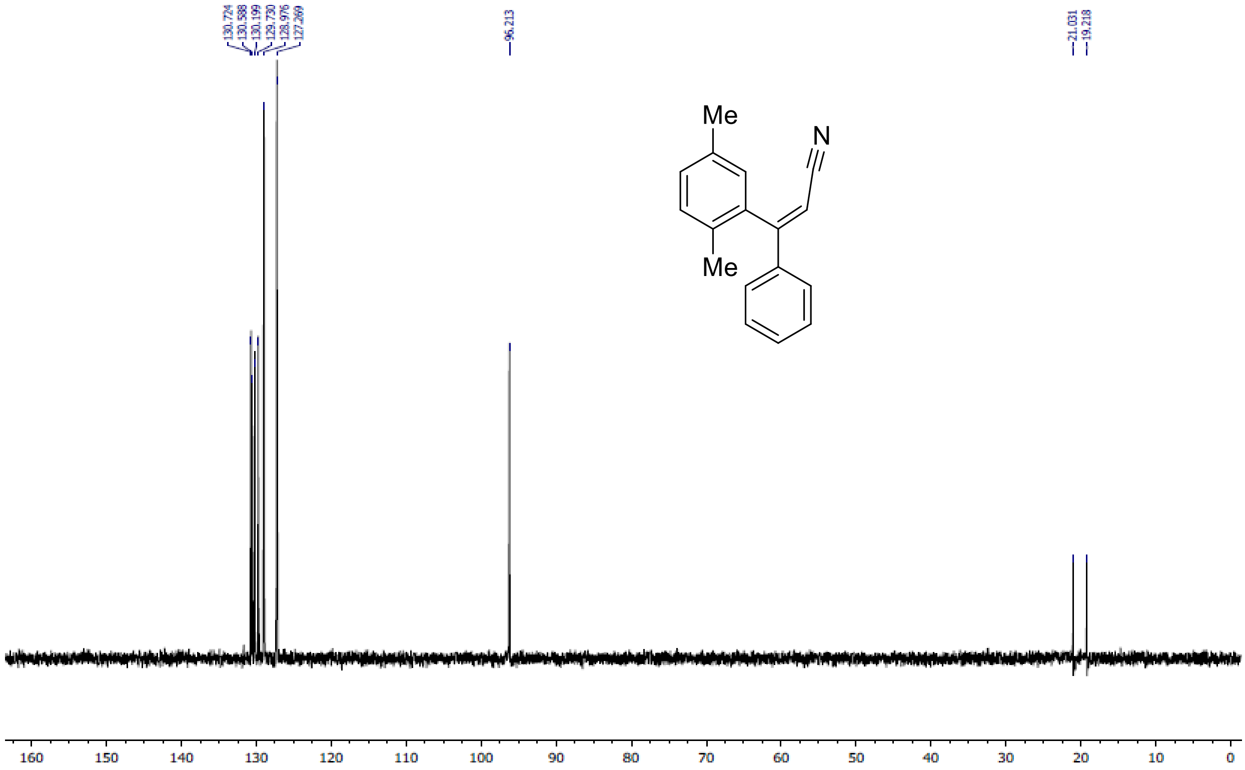

Figure S18. DEPT[135] NMR spectrum of compound **2c** ($CDCl_3$, 400 MHz).

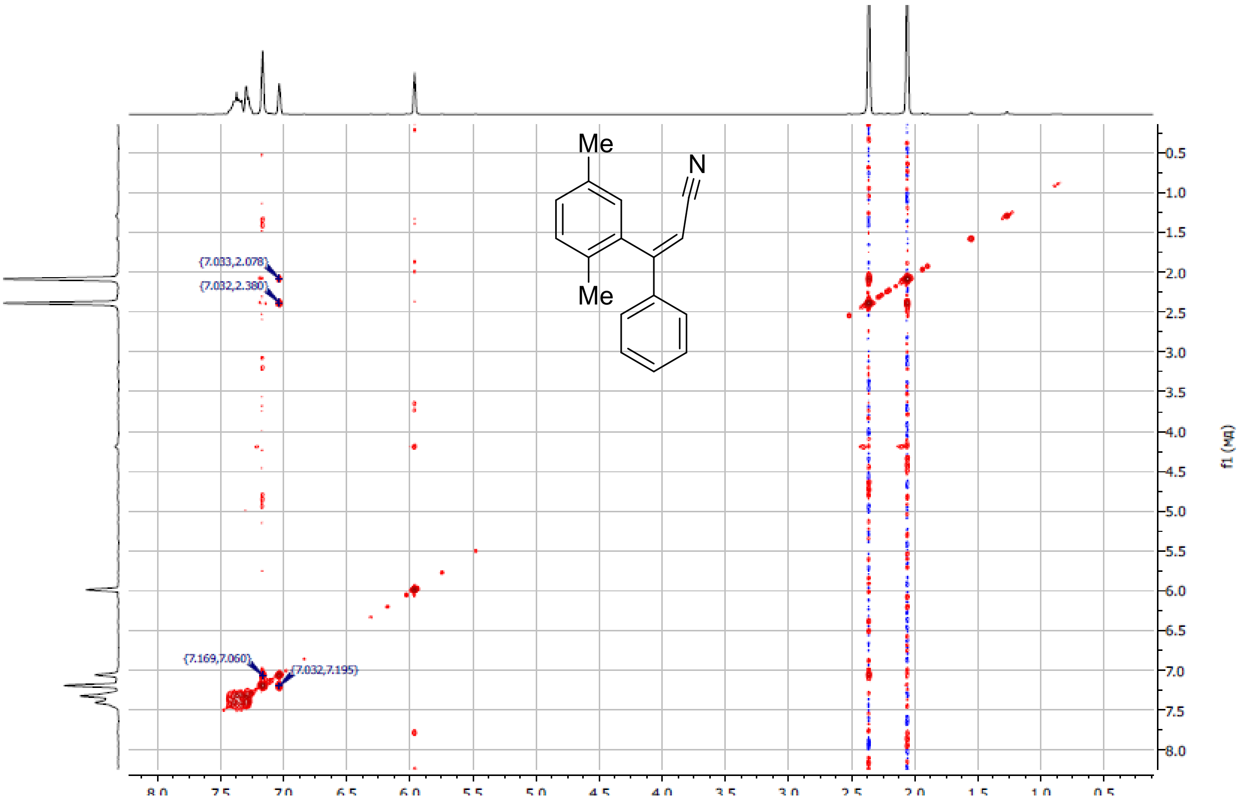

Figure S19. COSY NMR spectrum of compound **2c** ($CDCl_3$, 400 MHz).

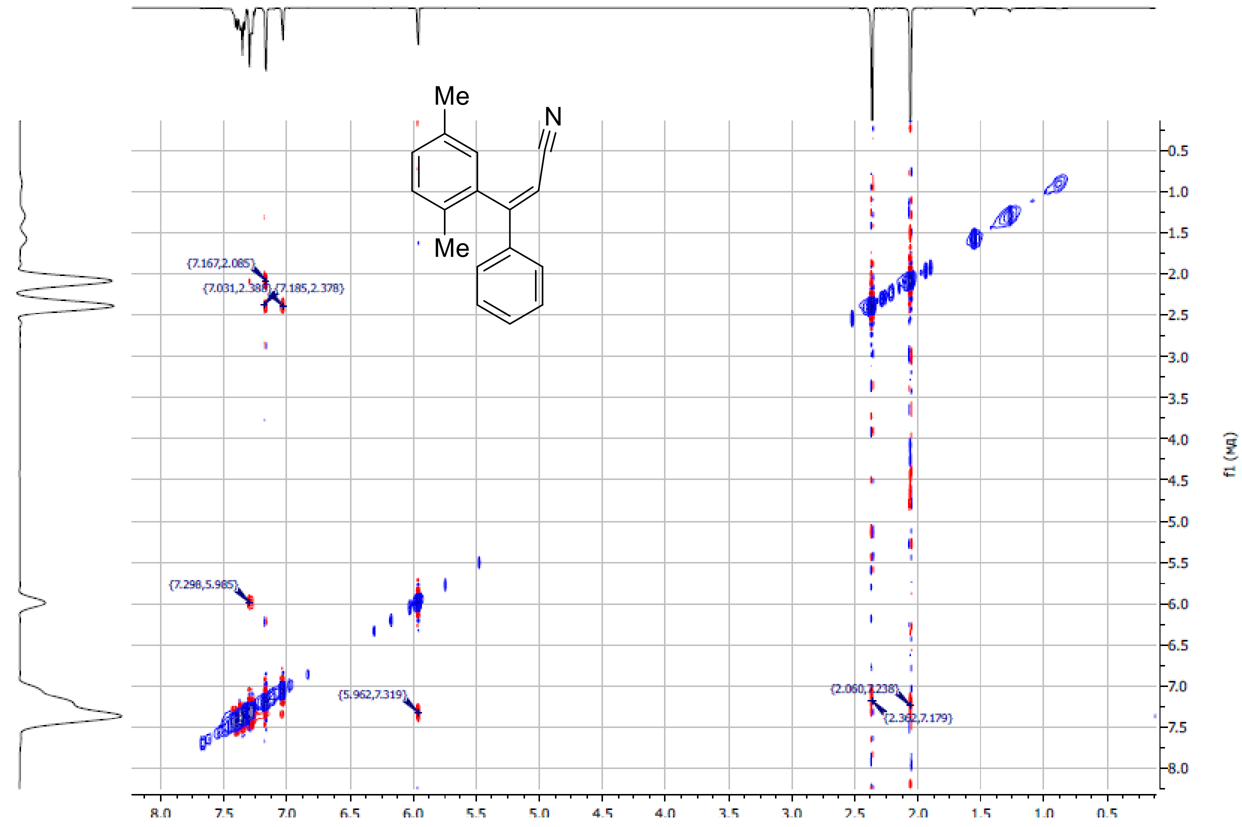

Figure S20. NOESY NMR spectrum of compound **2c** ($CDCl_3$, 400 MHz).

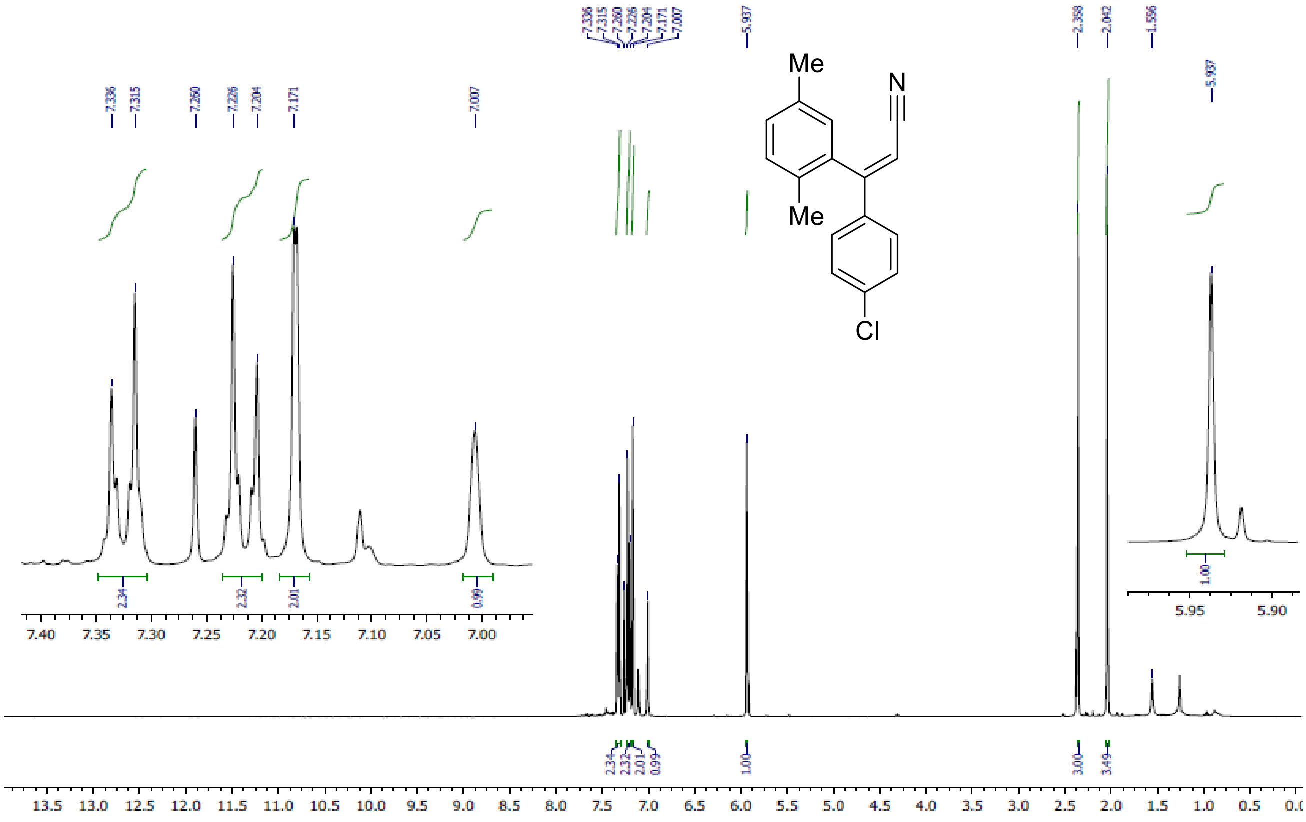

Figure S21. ^{1}H NMR spectrum of compound **2d** ($CDCl_3$, 400 MHz).

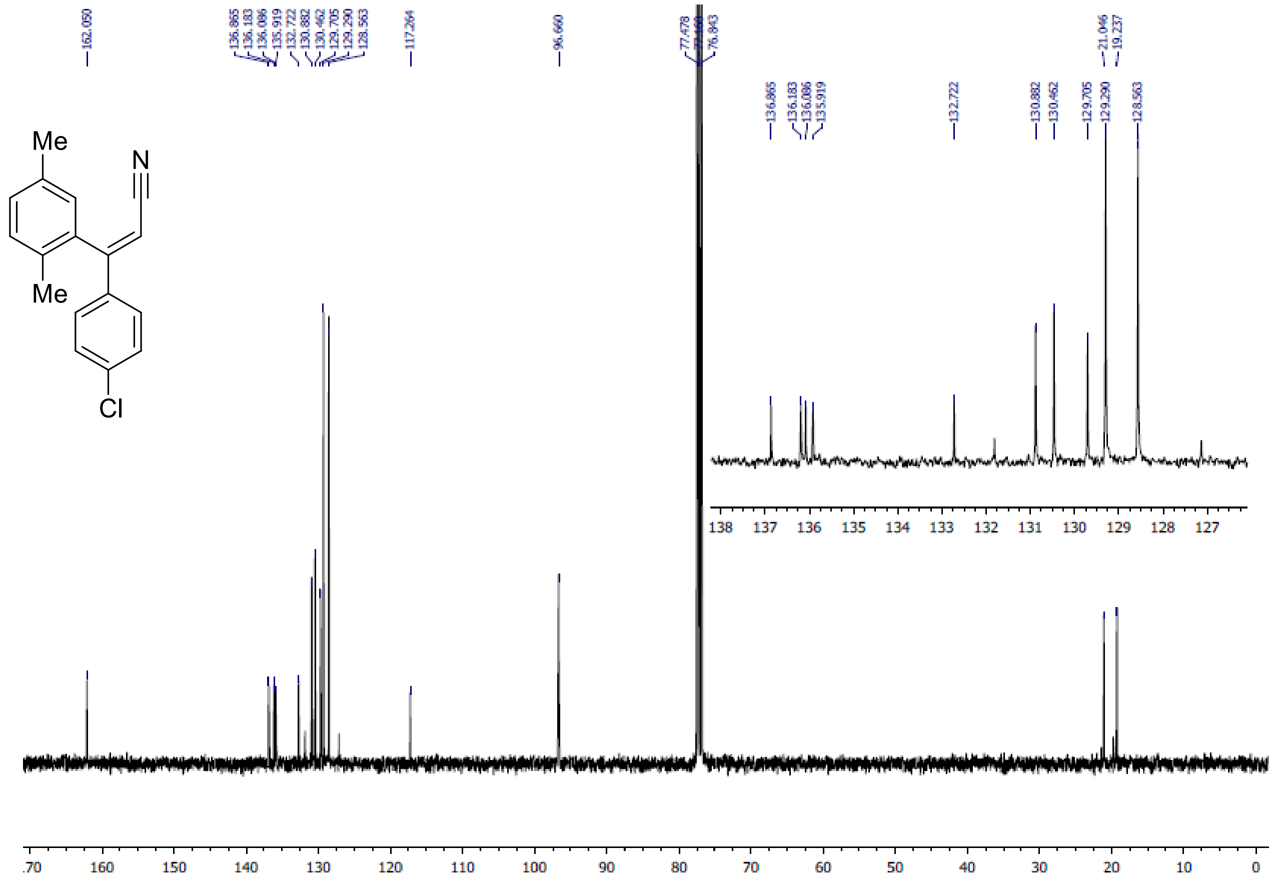

Figure S22. ^{13}C NMR spectrum of compound **2d** ($CDCl_3$, 100 MHz).

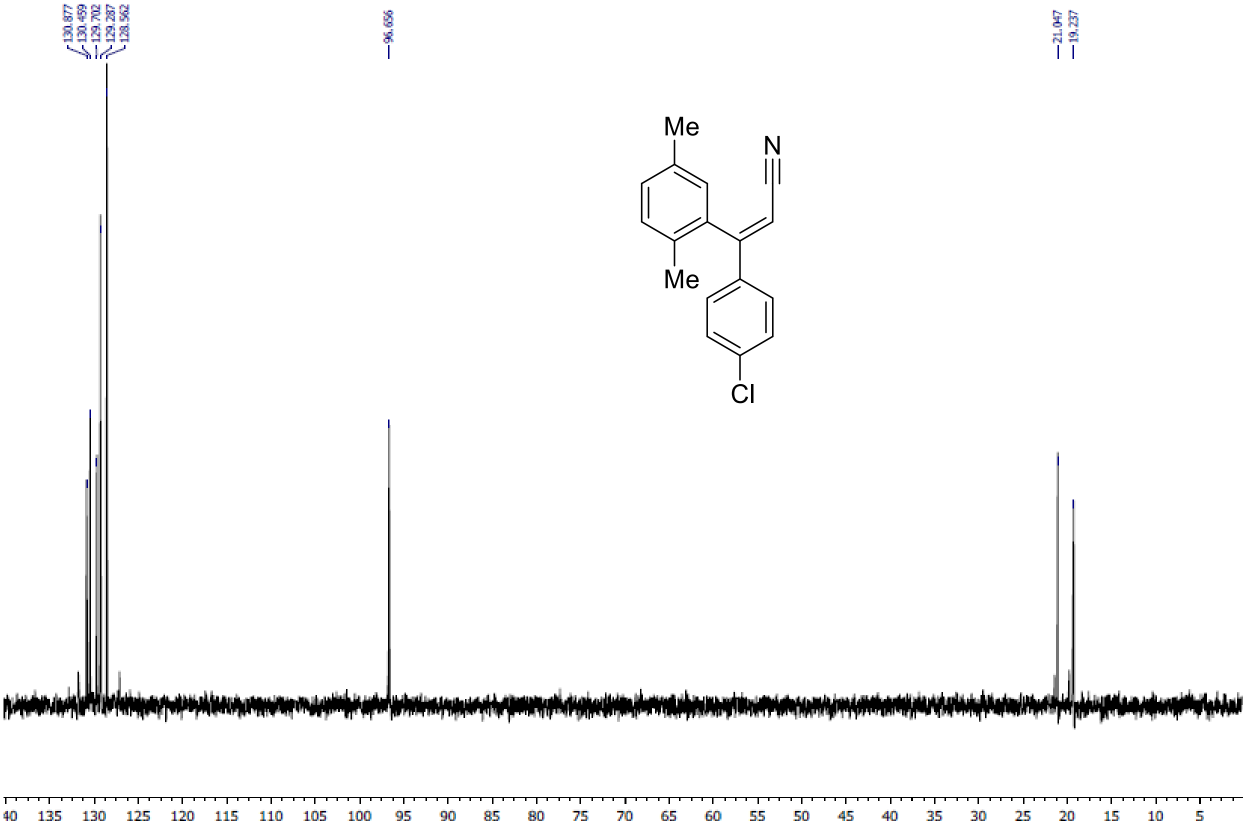

Figure S23. DEPT^{135} NMR spectrum of compound **2d** ($CDCl_3$, 400 MHz).

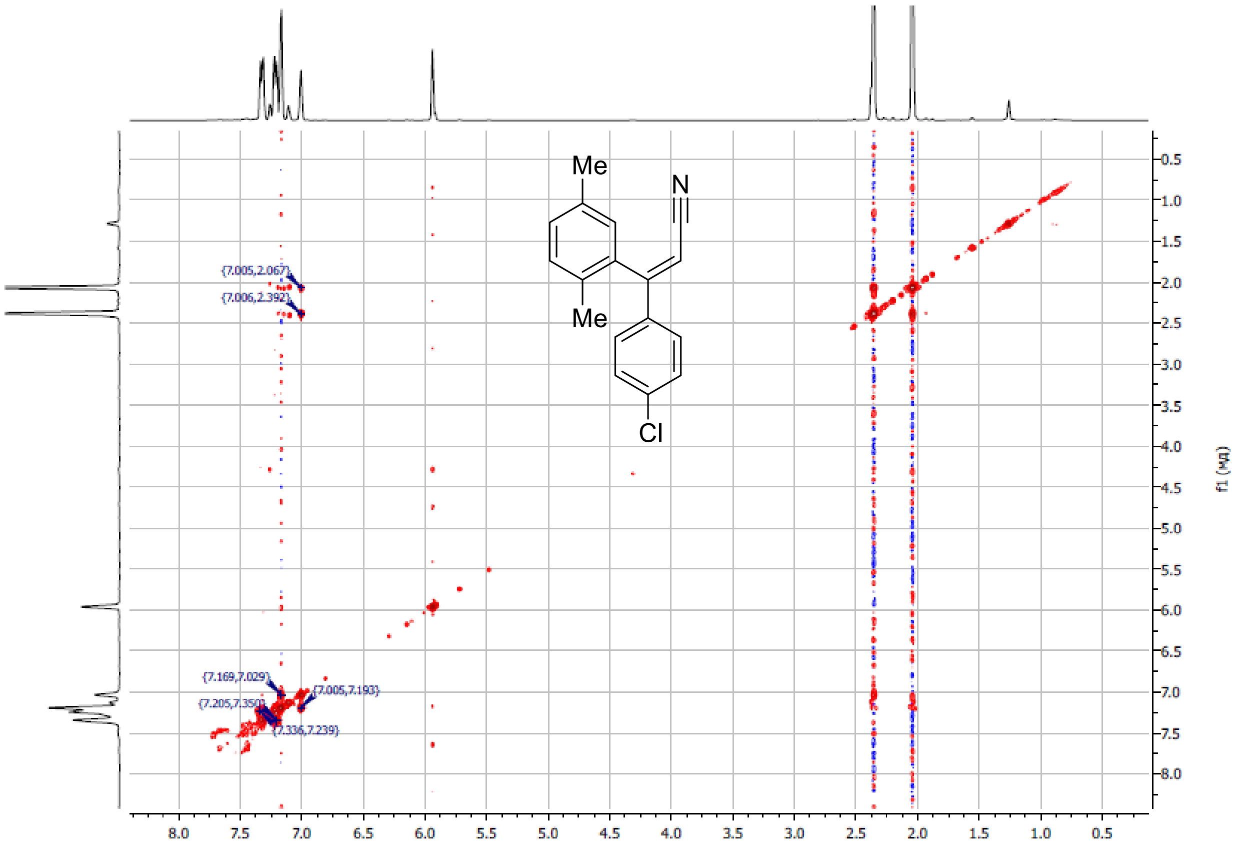

Figure S24. COSY NMR spectrum of compound **2d** ($CDCl_3$, 400 MHz).

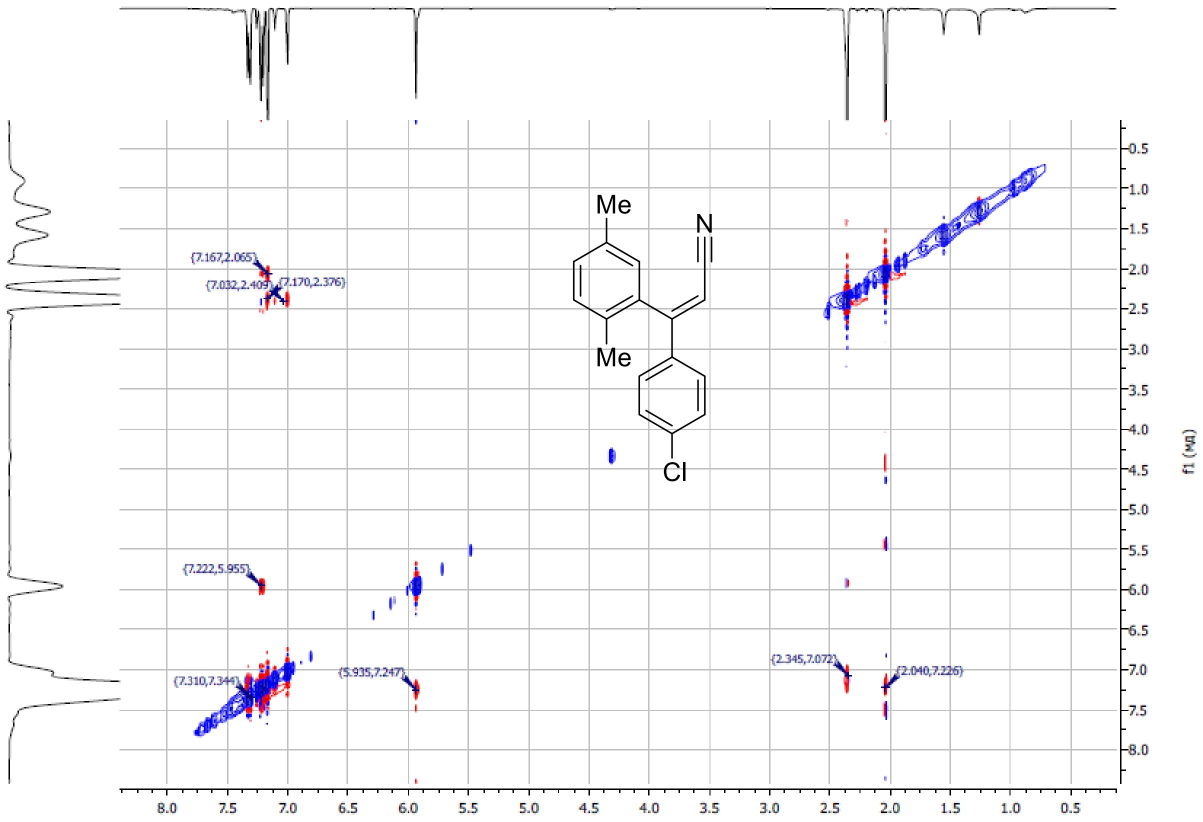

Figure S25. NOESY NMR spectrum of compound **2d** ($CDCl_3$, 400 MHz).

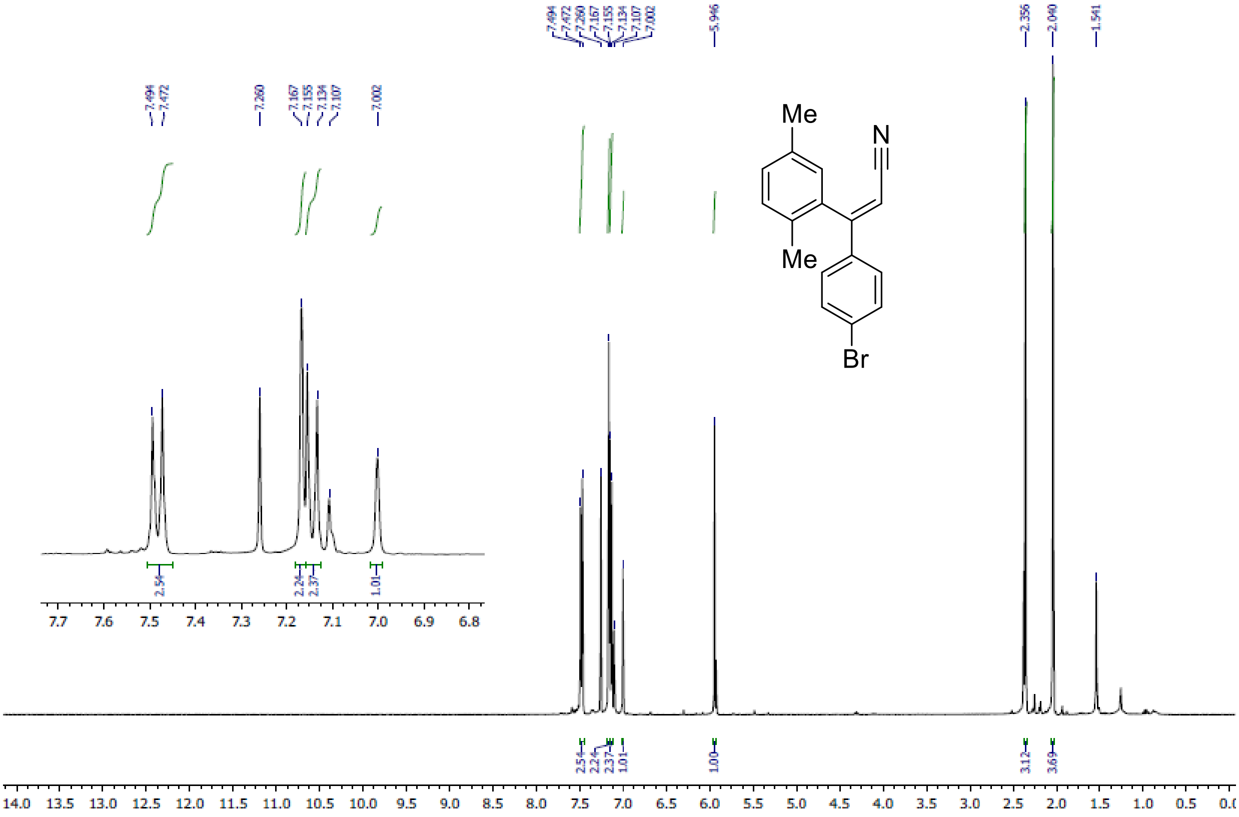

Figure S26. ^{1}H NMR spectrum of compound **2e** ($CDCl_3$, 400 MHz).

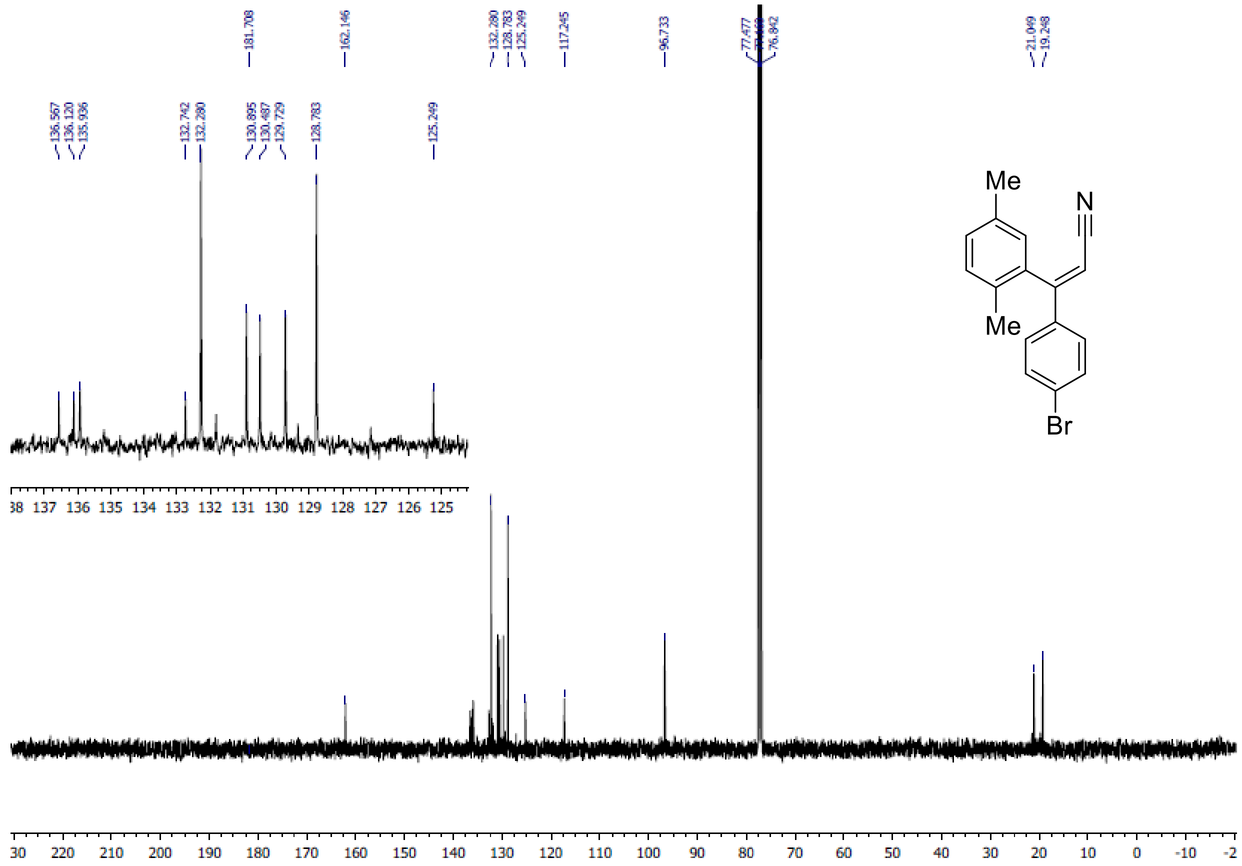

Figure S27. ^{13}C NMR spectrum of compound **2e** ($CDCl_3$, 100 MHz).

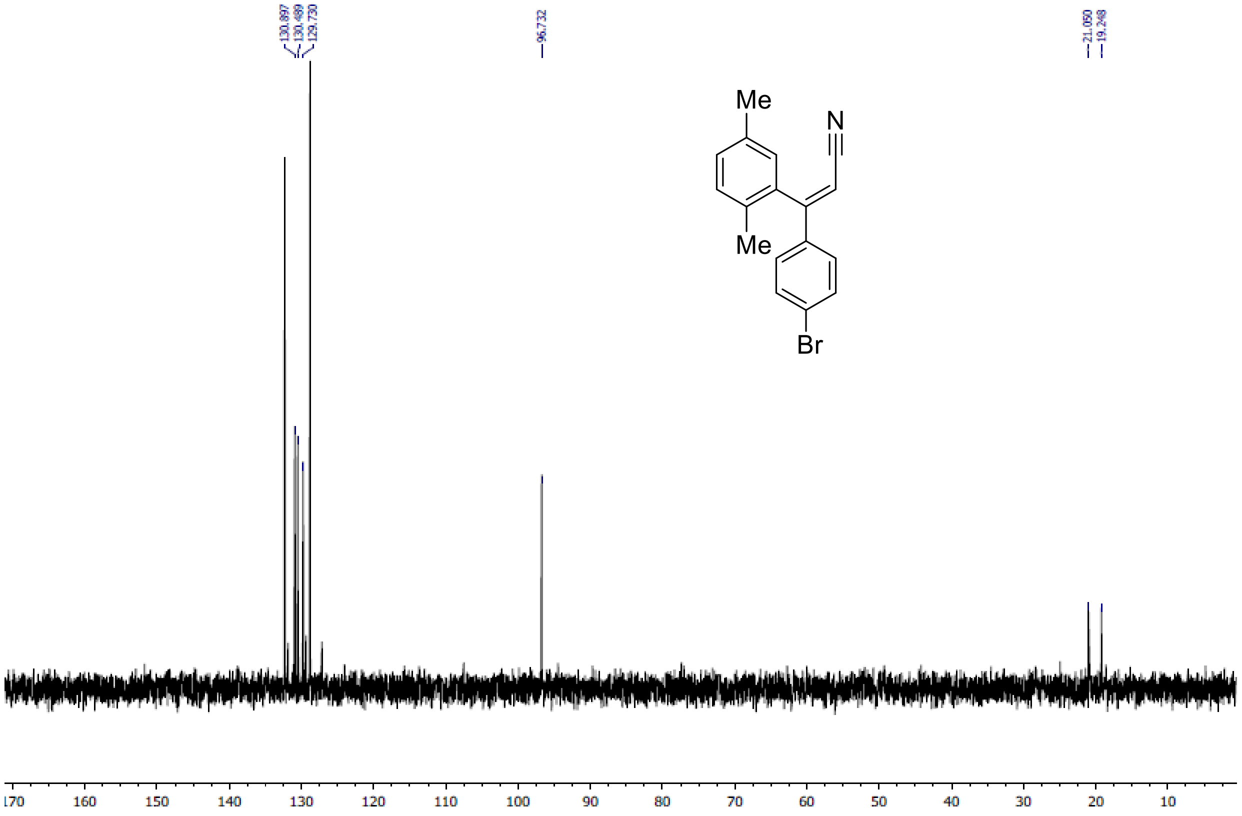

Figure S28. DEPT^{135} NMR spectrum of compound **2e** ($CDCl_3$, 400 MHz).

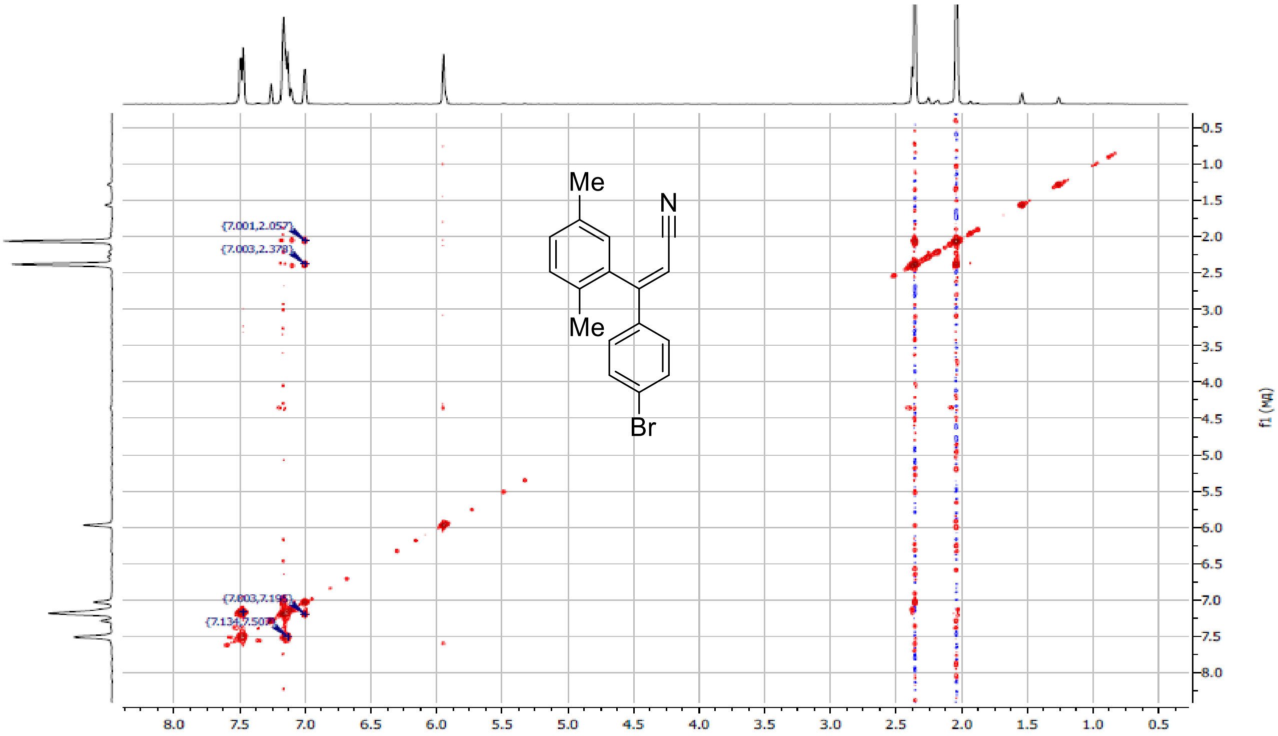

Figure S29. COSY NMR spectrum of compound **2e** ($CDCl_3$, 400 MHz).

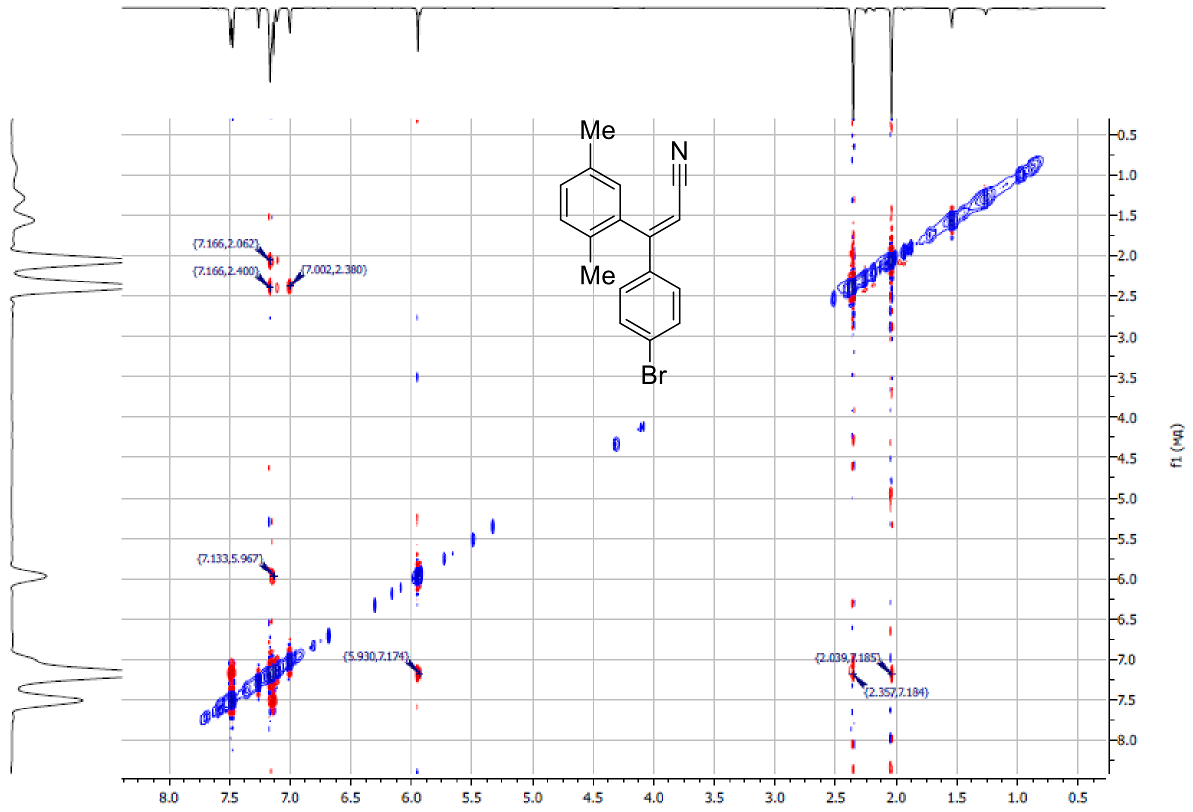

Figure S30. NOESY NMR spectrum of compound **2e** ($CDCl_3$, 400 MHz).

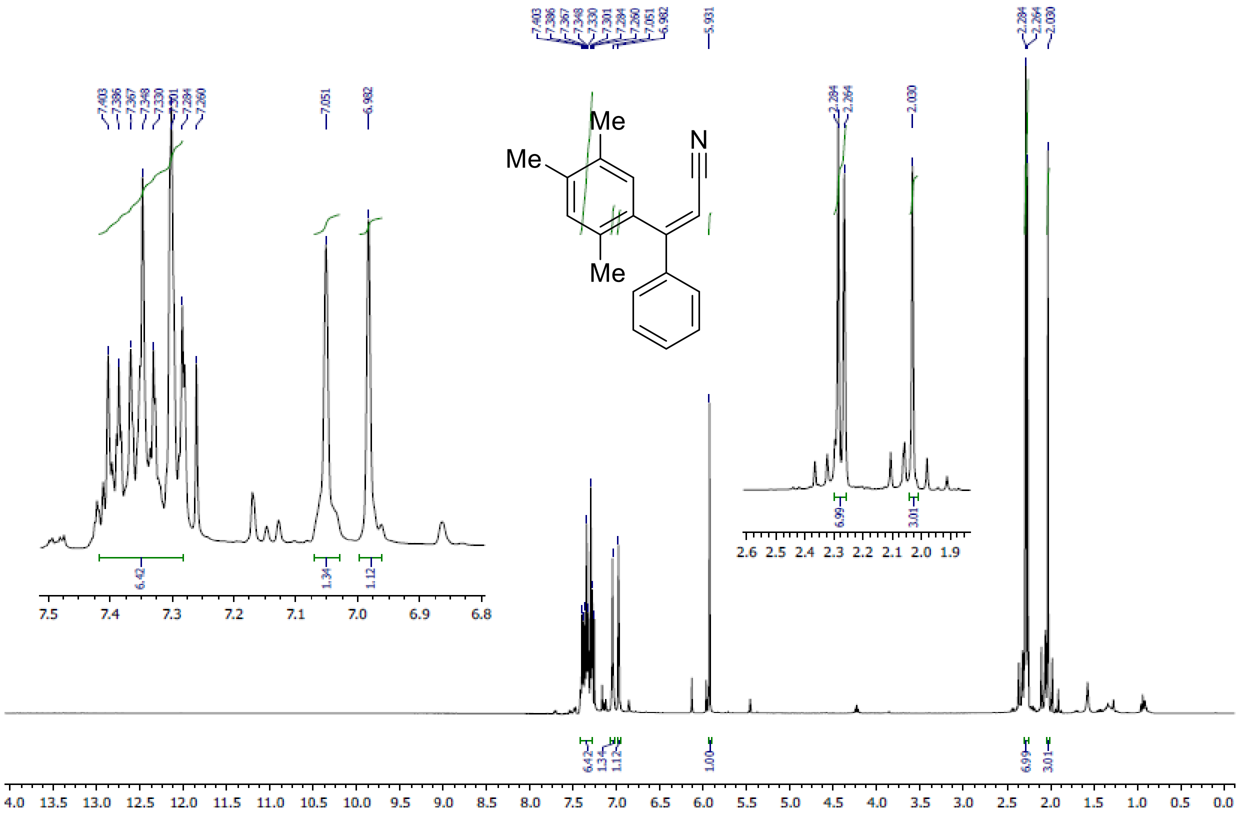

Figure S31. ^{1}H NMR spectrum of compound **2f** ($CDCl_3$, 400 MHz).

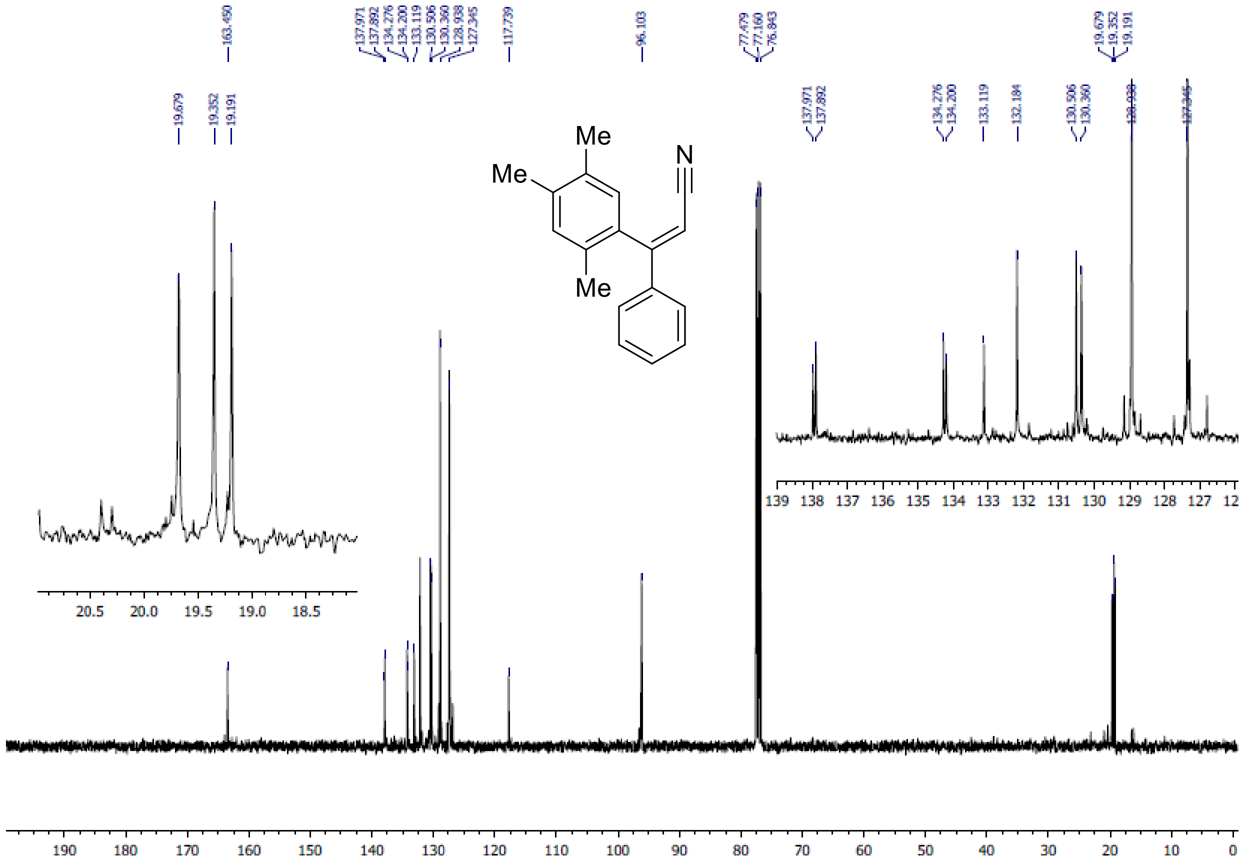

Figure S32. ^{13}C NMR spectrum of compound **2f** ($CDCl_3$, 100 MHz).

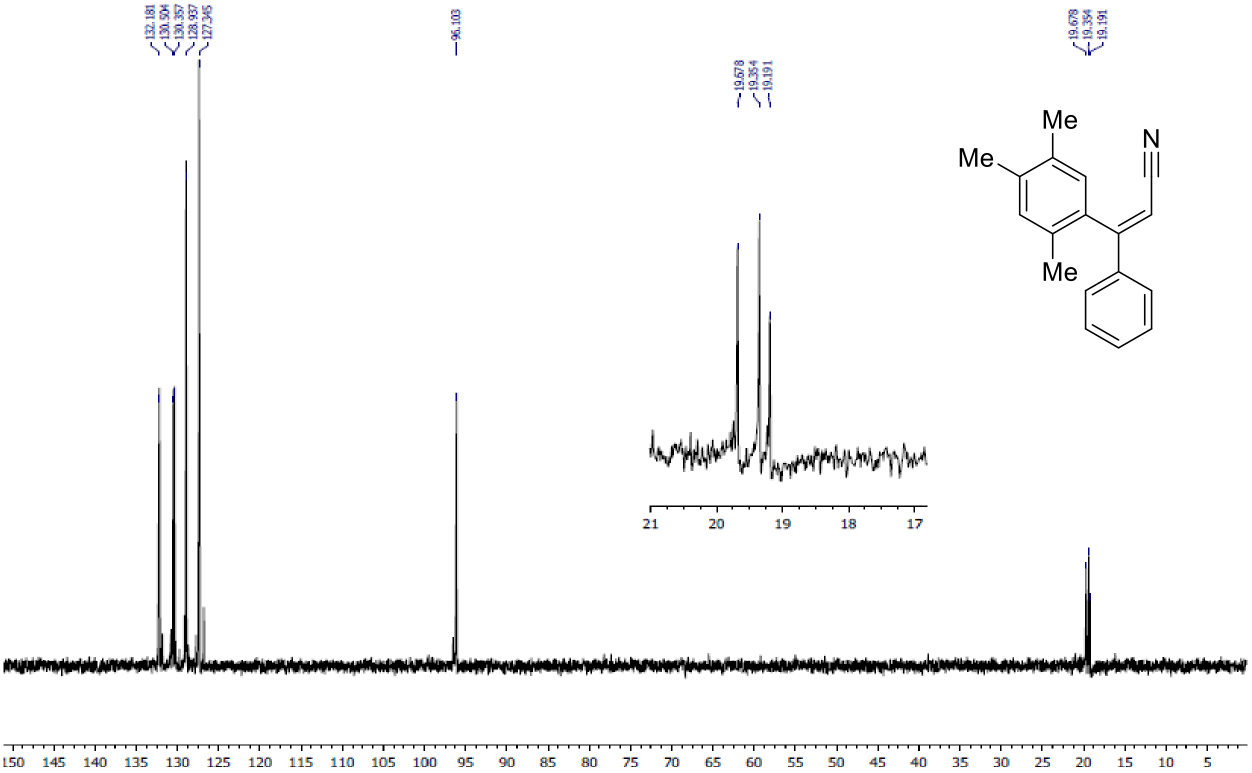

Figure S33. DEPT135 NMR spectrum of compound **2f** ($CDCl_3$, 400 MHz)

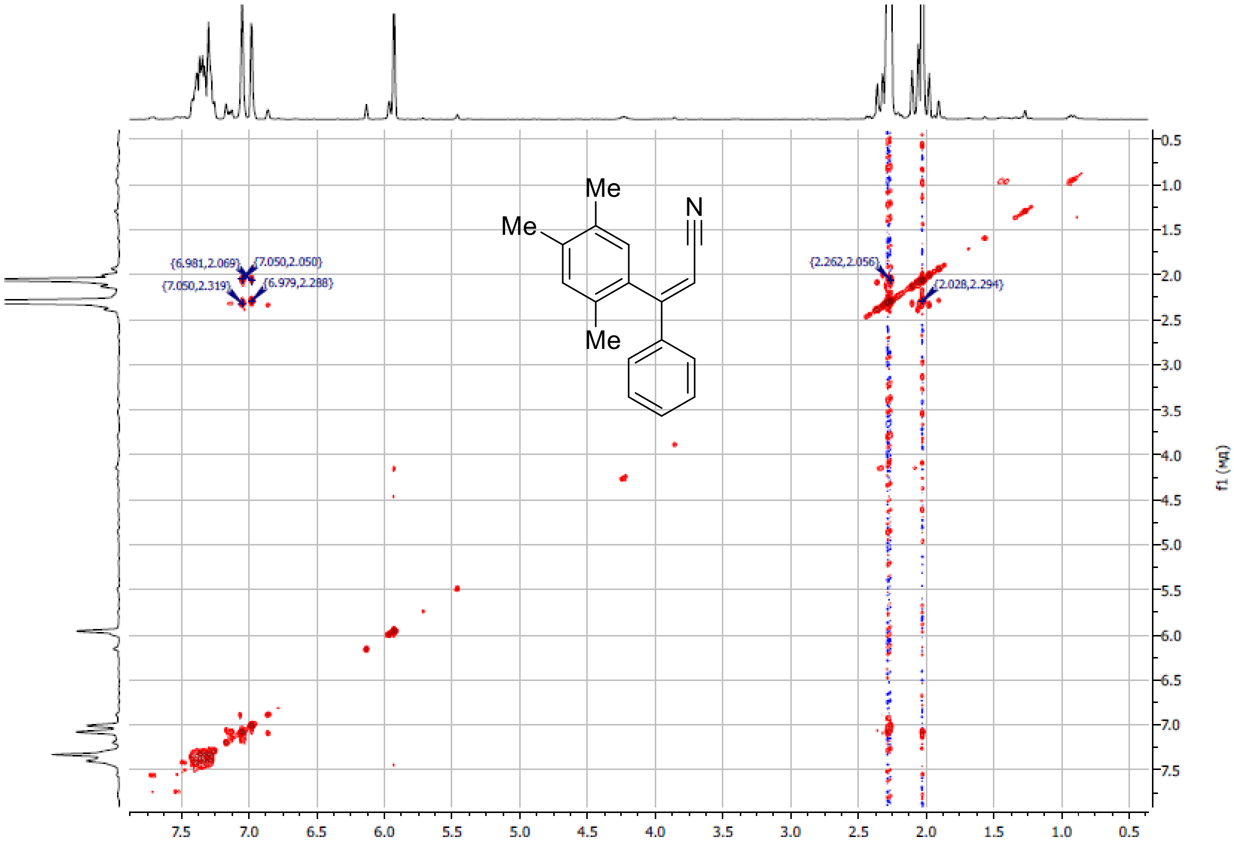

Figure S34. COSY NMR spectrum of compound **2f** ($CDCl_3$, 400 MHz).

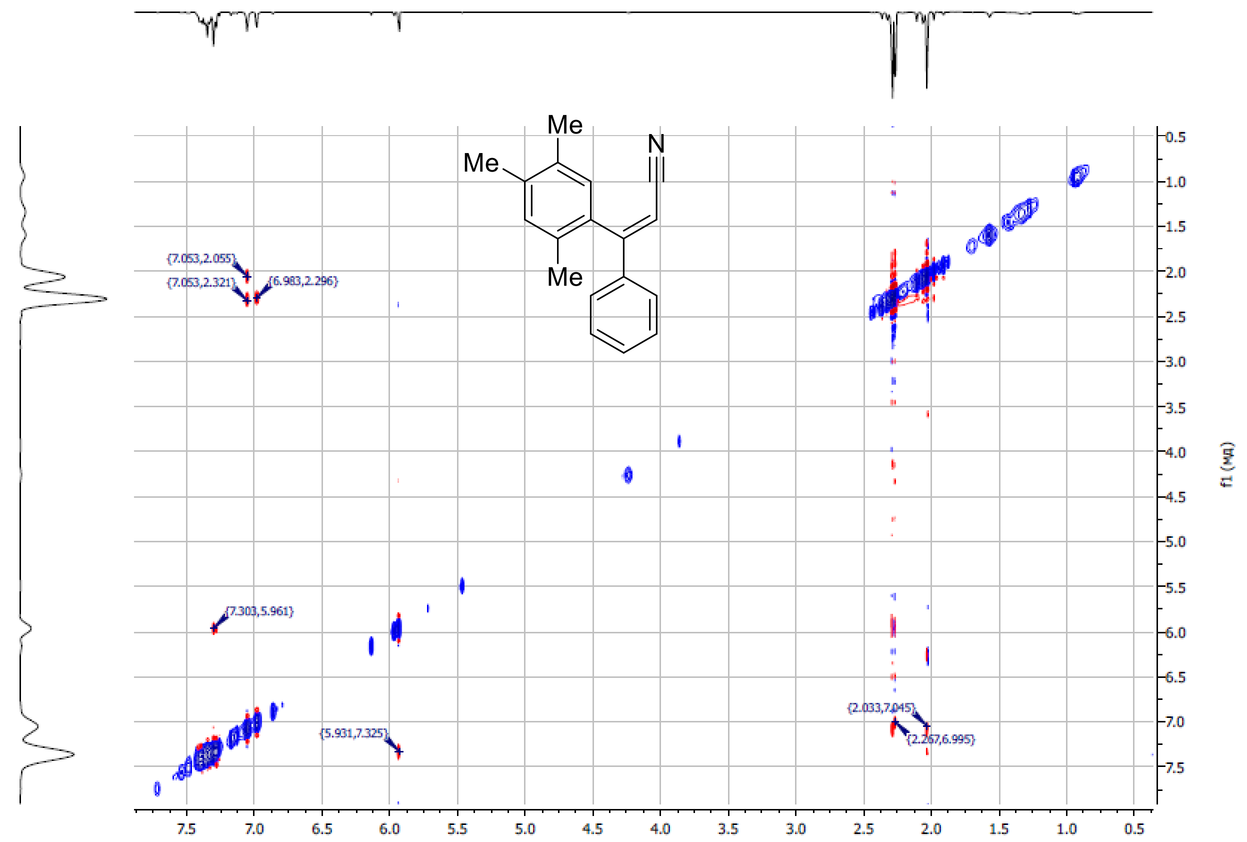

Figure S35. NOESY NMR spectrum of compound **2f** ($CDCl_3$, 400 MHz).

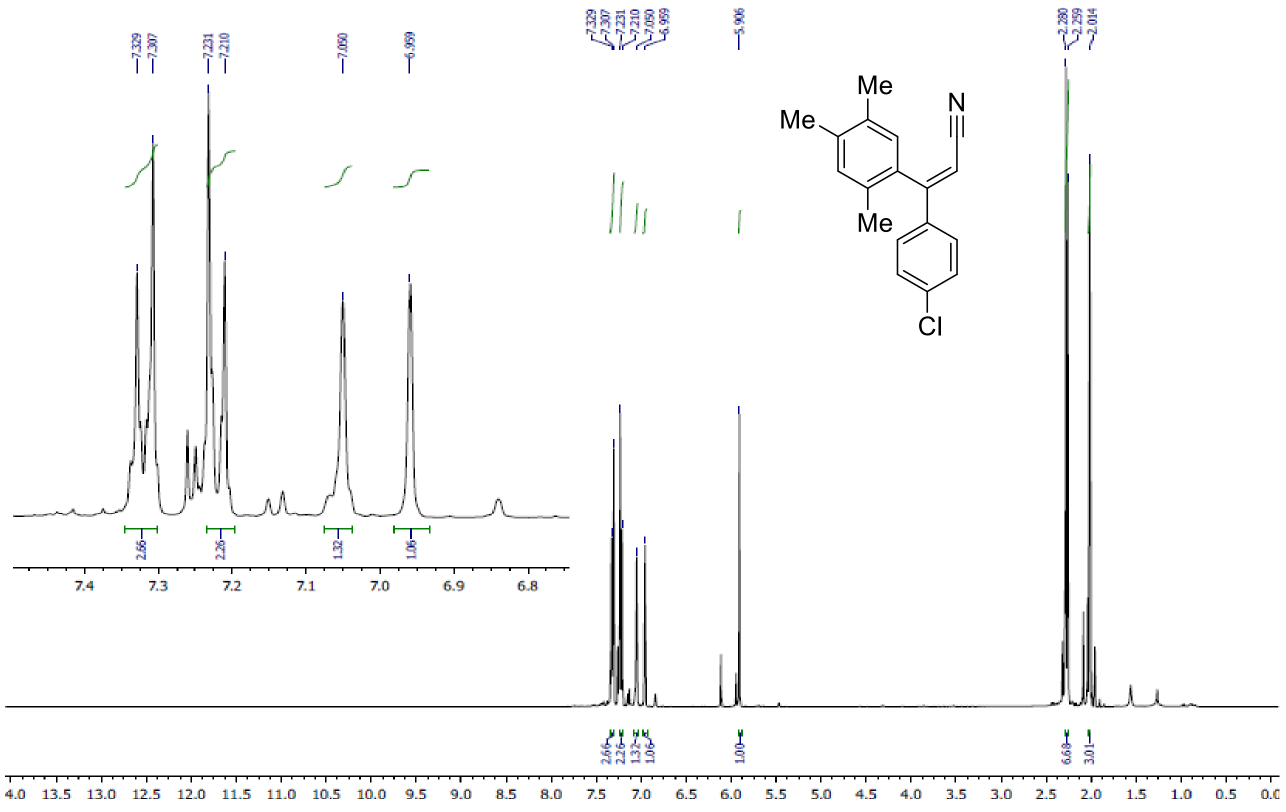

Figure S36. ^{1}H NMR spectrum of compound **2g** ($CDCl_3$, 400 MHz).

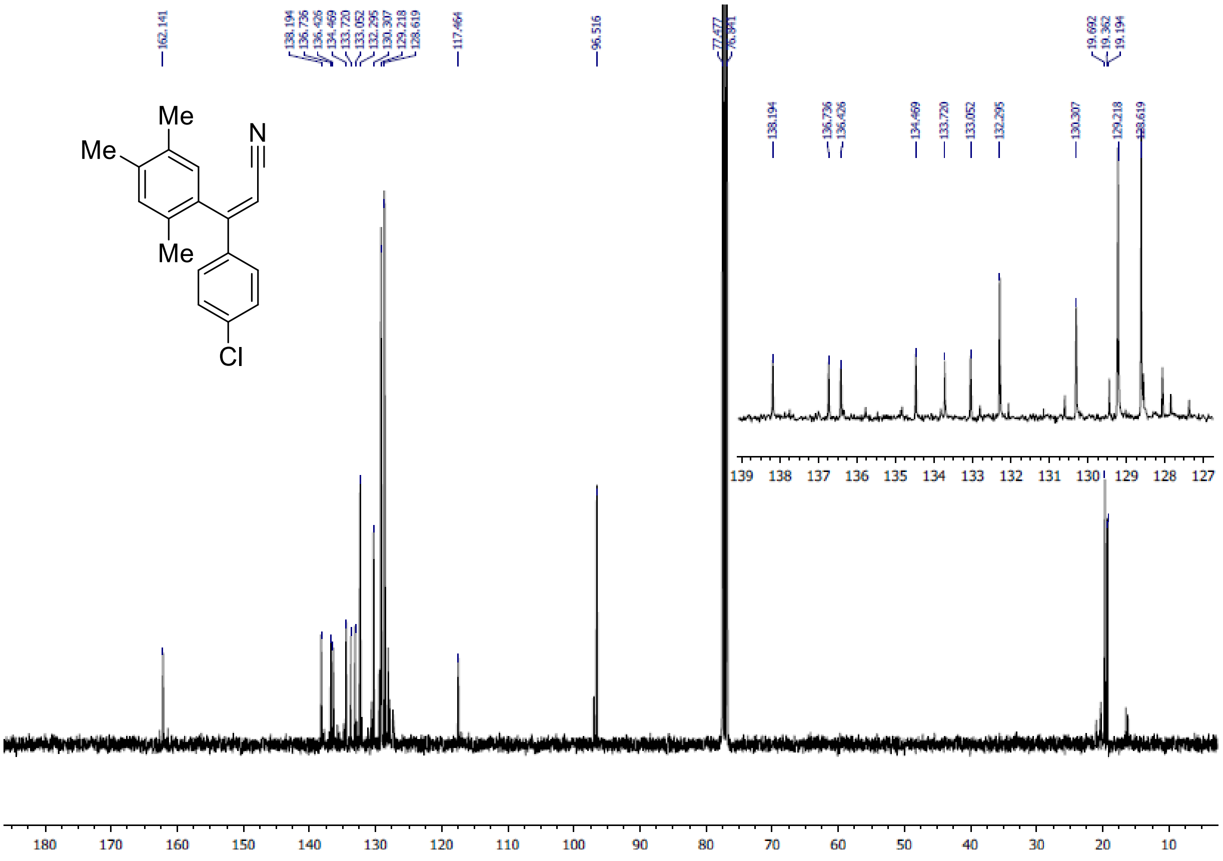

Figure S37. ^{13}C NMR spectrum of compound **2g** ($CDCl_3$, 100 MHz).

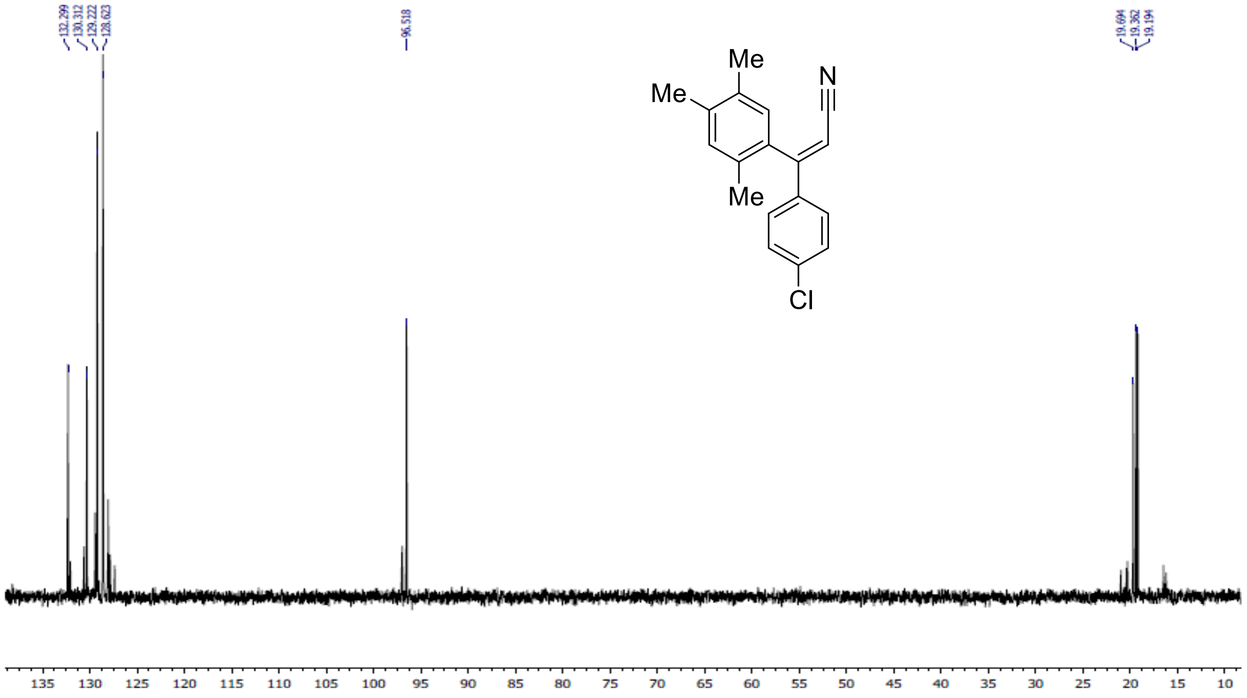

Figure S38. DEPT^{135} NMR spectrum of compound **2g** ($CDCl_3$, 400 MHz).

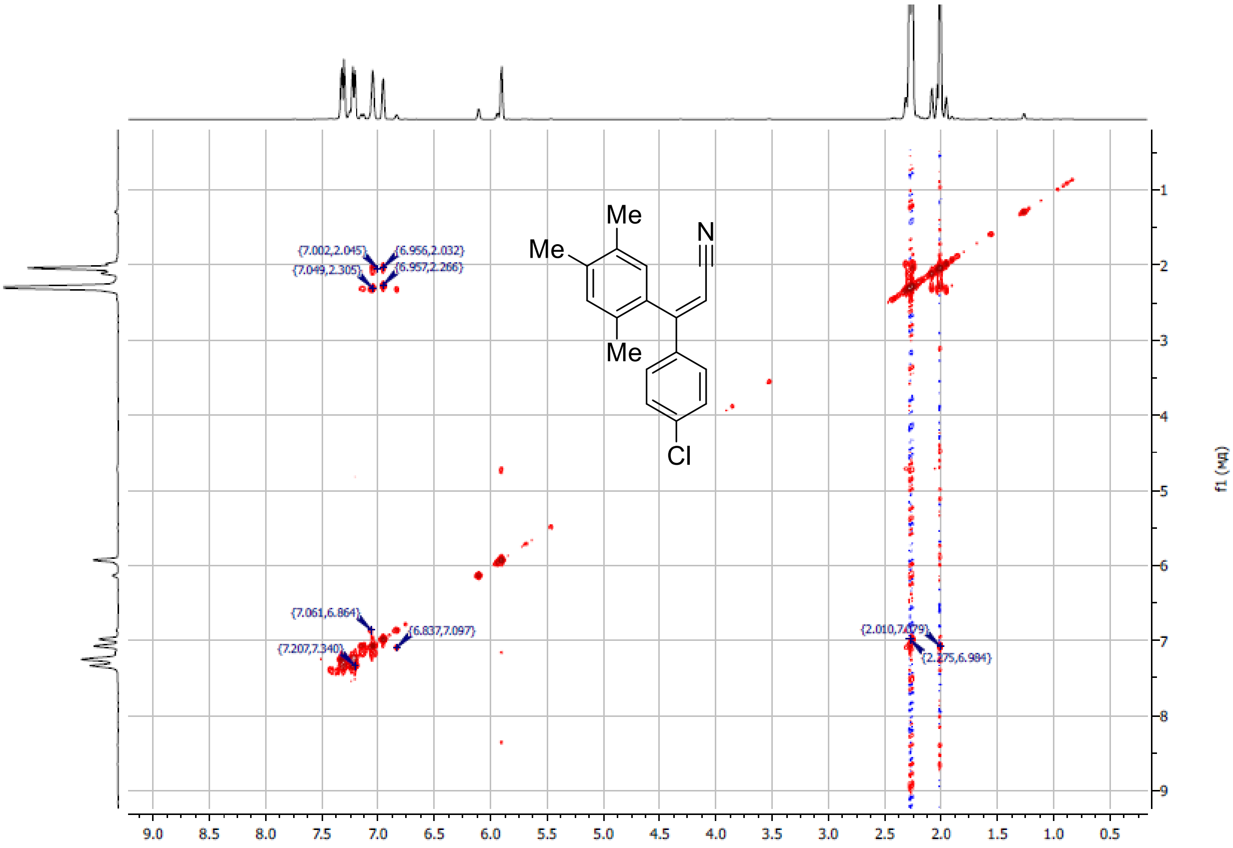

Figure S39. COSY NMR spectrum of compound **2g** ($CDCl_3$, 400 MHz).

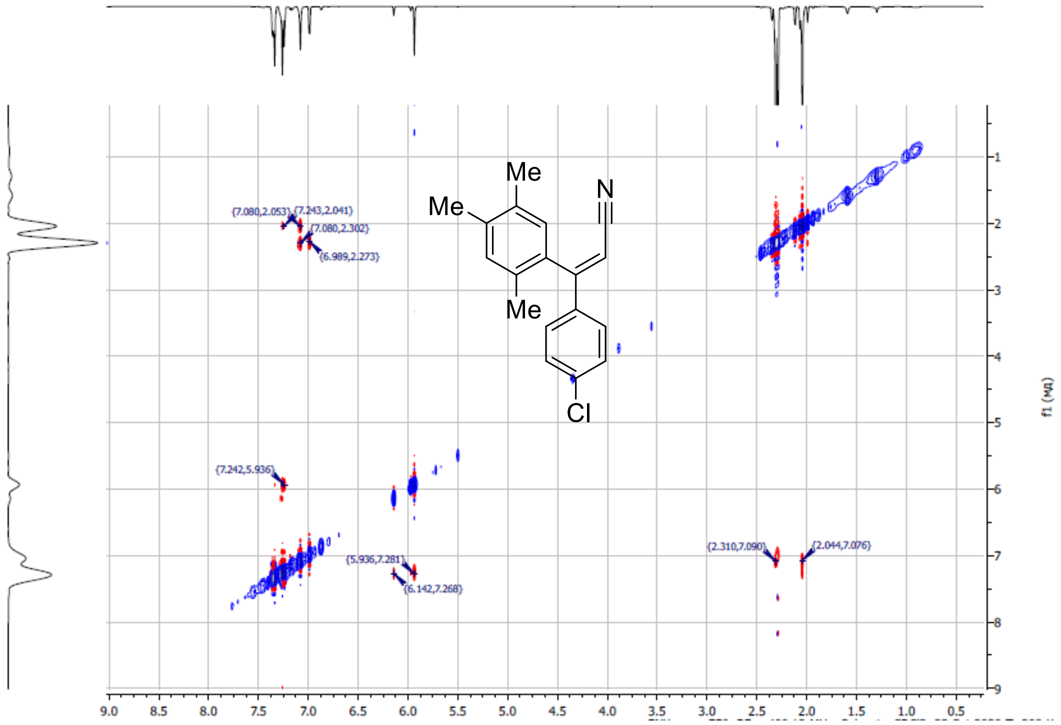

Figure S40. NOESY NMR spectrum of compound **2g** ($CDCl_3$, 400 MHz).

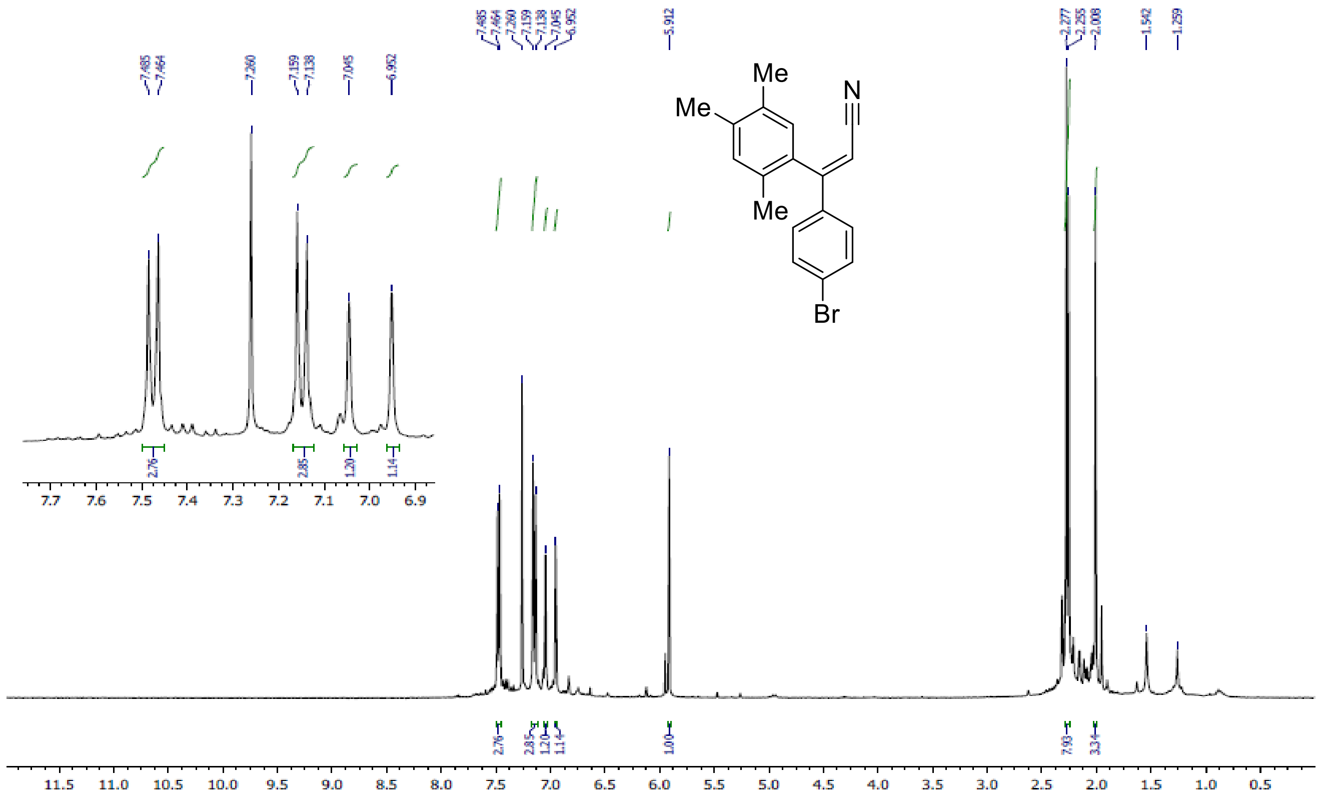

Figure S41. ^{1}H NMR spectrum of compound **2h** ($CDCl_3$, 400 MHz).

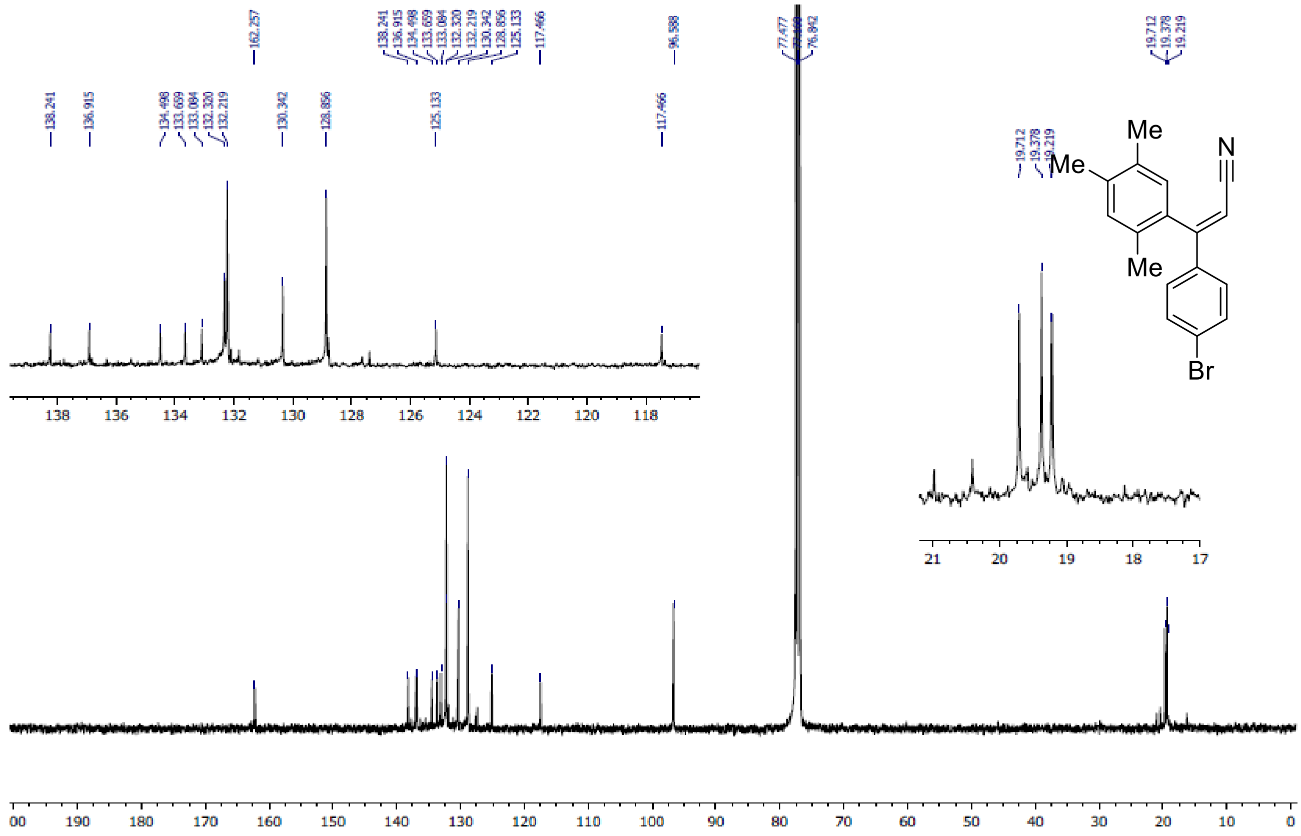

Figure S42. ^{13}C NMR spectrum of compound **2h** ($CDCl_3$, 100 MHz).

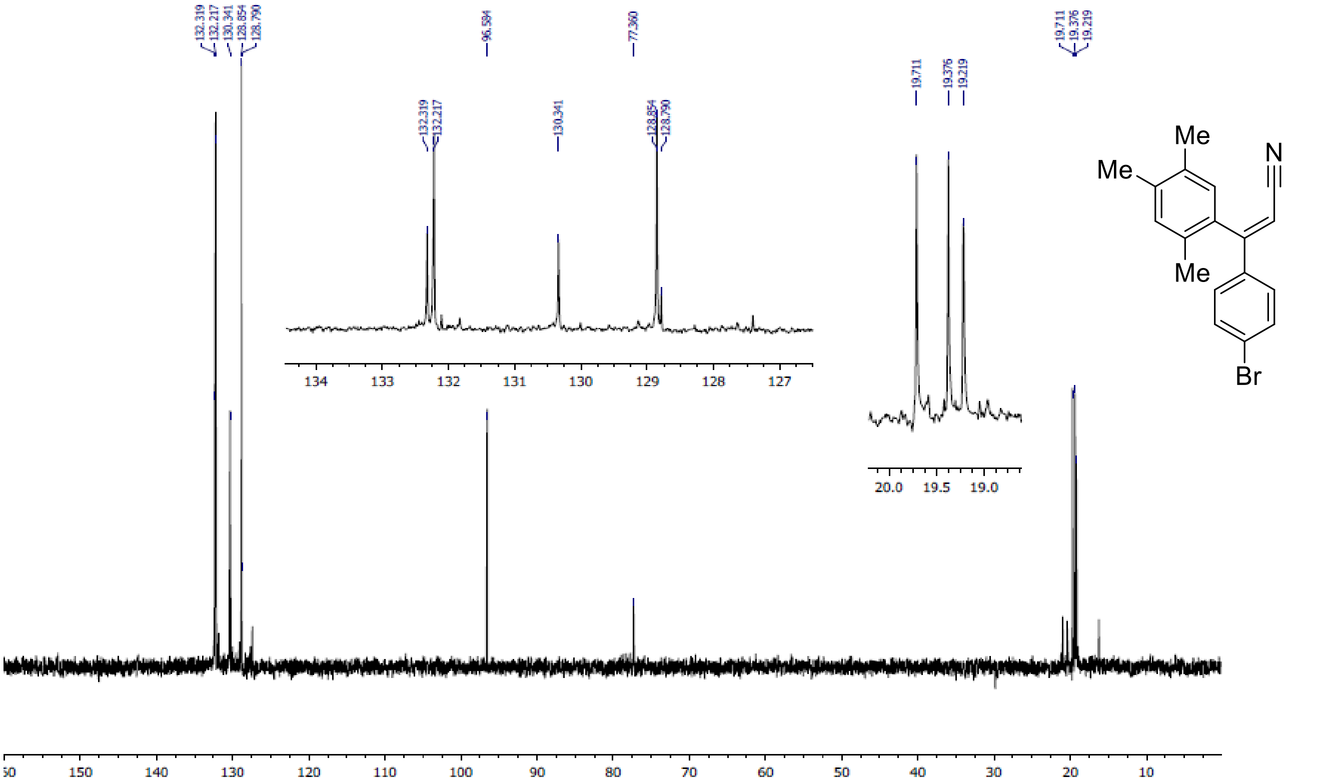

Figure S43. DEPT[135] NMR spectrum of compound **2h** ($CDCl_3$, 400 MHz).

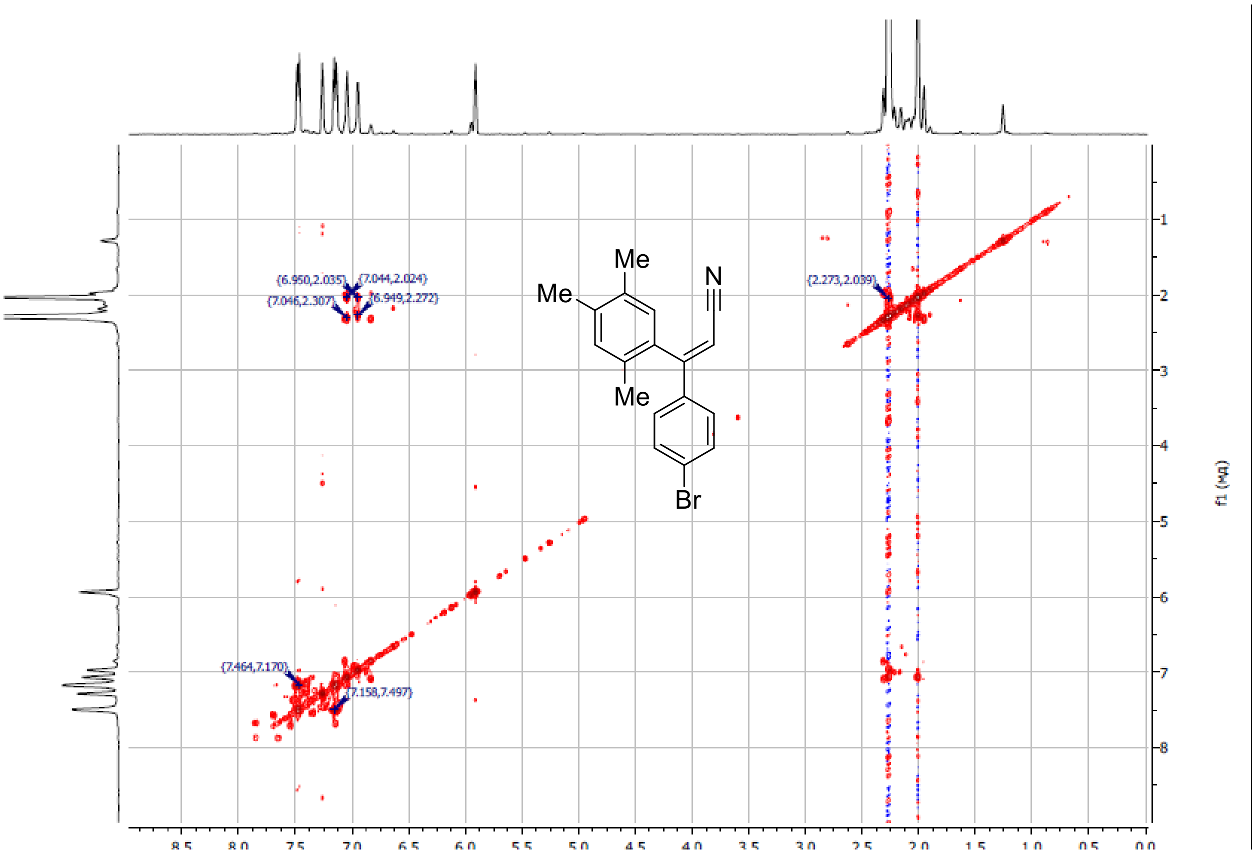

Figure S44. COSY NMR spectrum of compound **2h** ($CDCl_3$, 400 MHz).

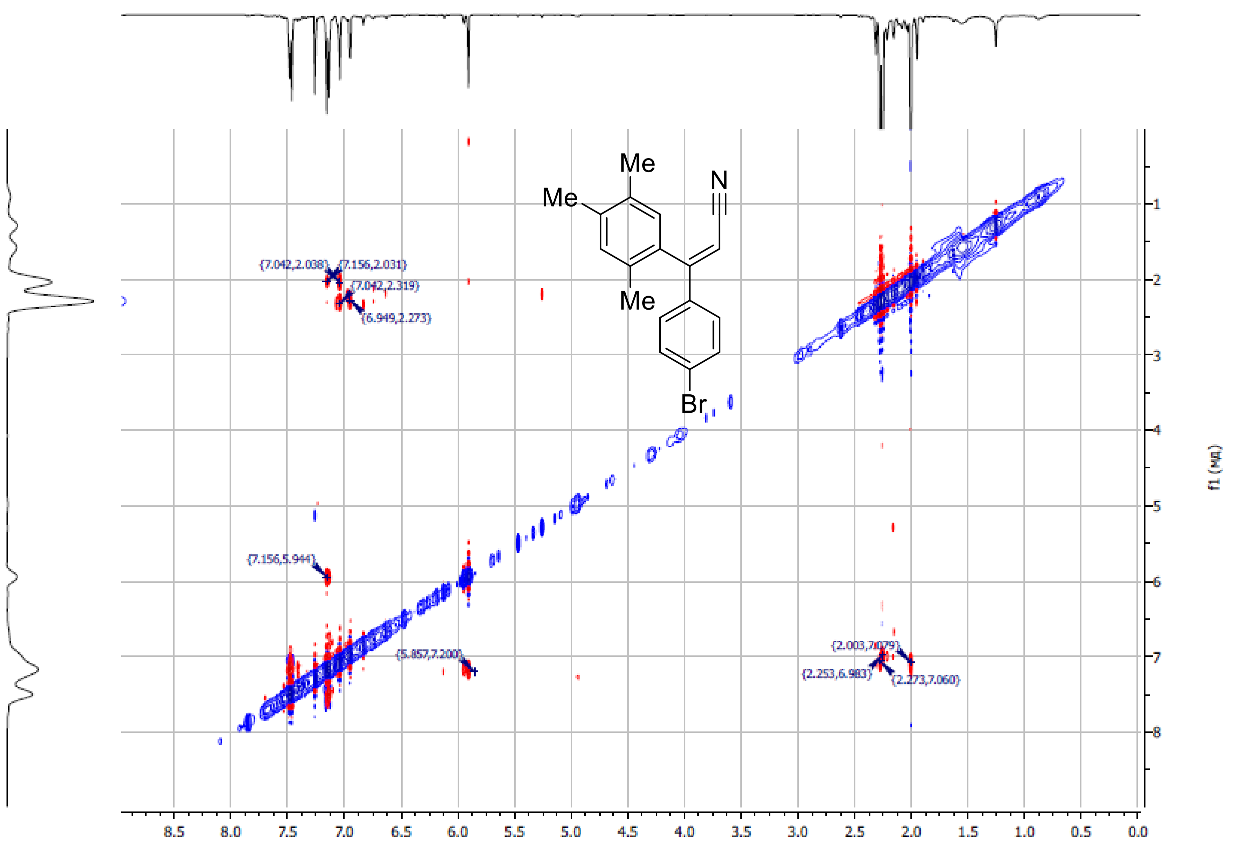

Figure S45. NOESY NMR spectrum of compound **2h** ($CDCl_3$, 400 MHz).

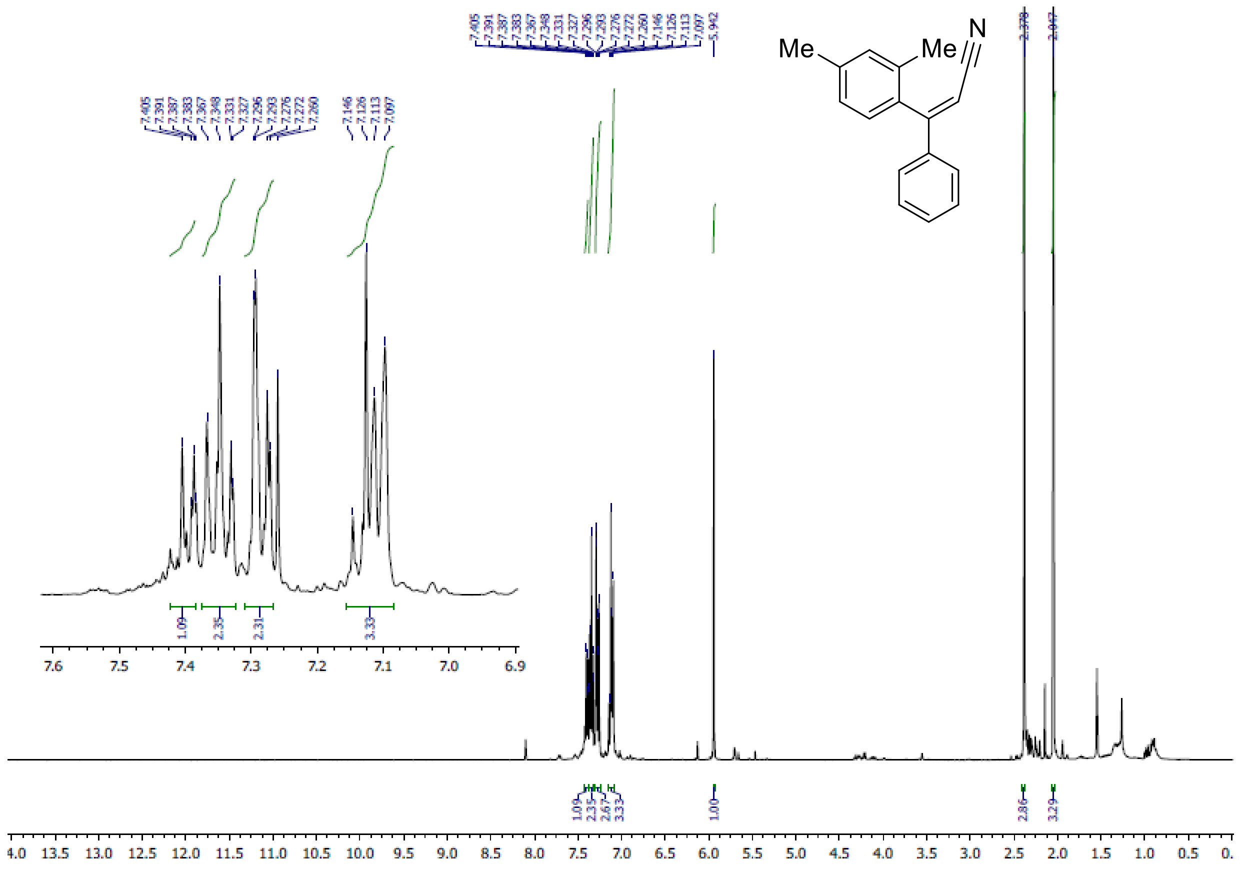

Figure S46. ^{1}H NMR spectrum of compound **2i** ($CDCl_3$, 400 MHz).

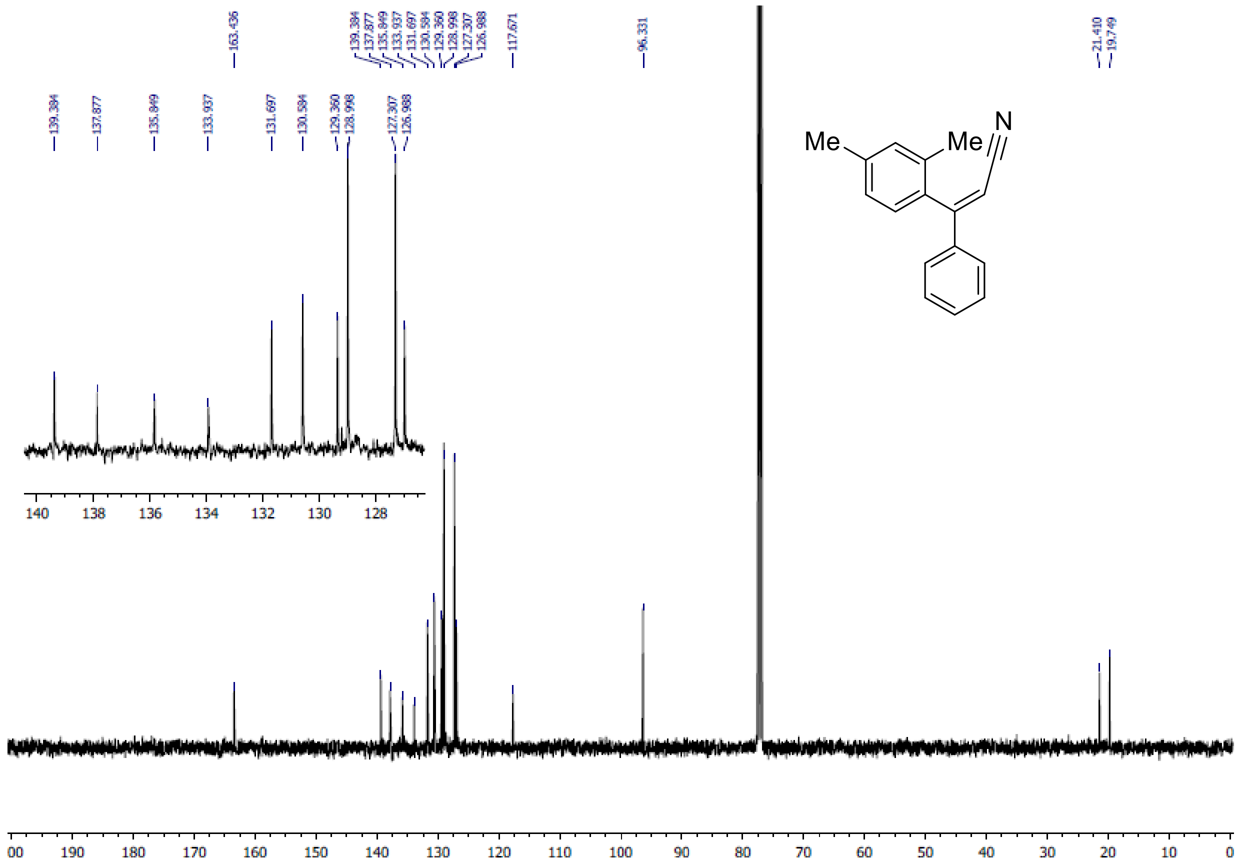

Figure S47. ^{13}C NMR spectrum of compound **2i** ($CDCl_3$, 100 MHz).

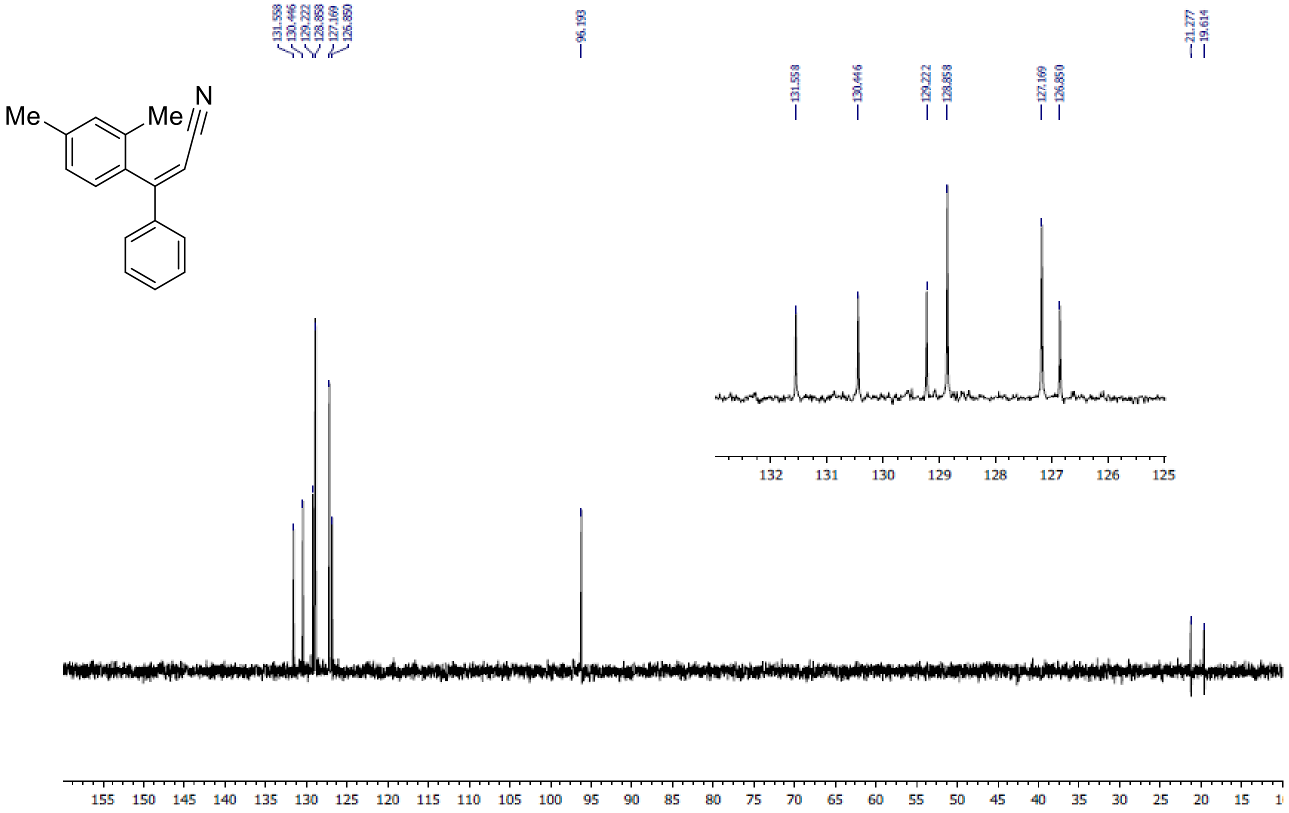

Figure S48. DEPT^{135} NMR spectrum of compound **2i** ($CDCl_3$, 400 MHz).

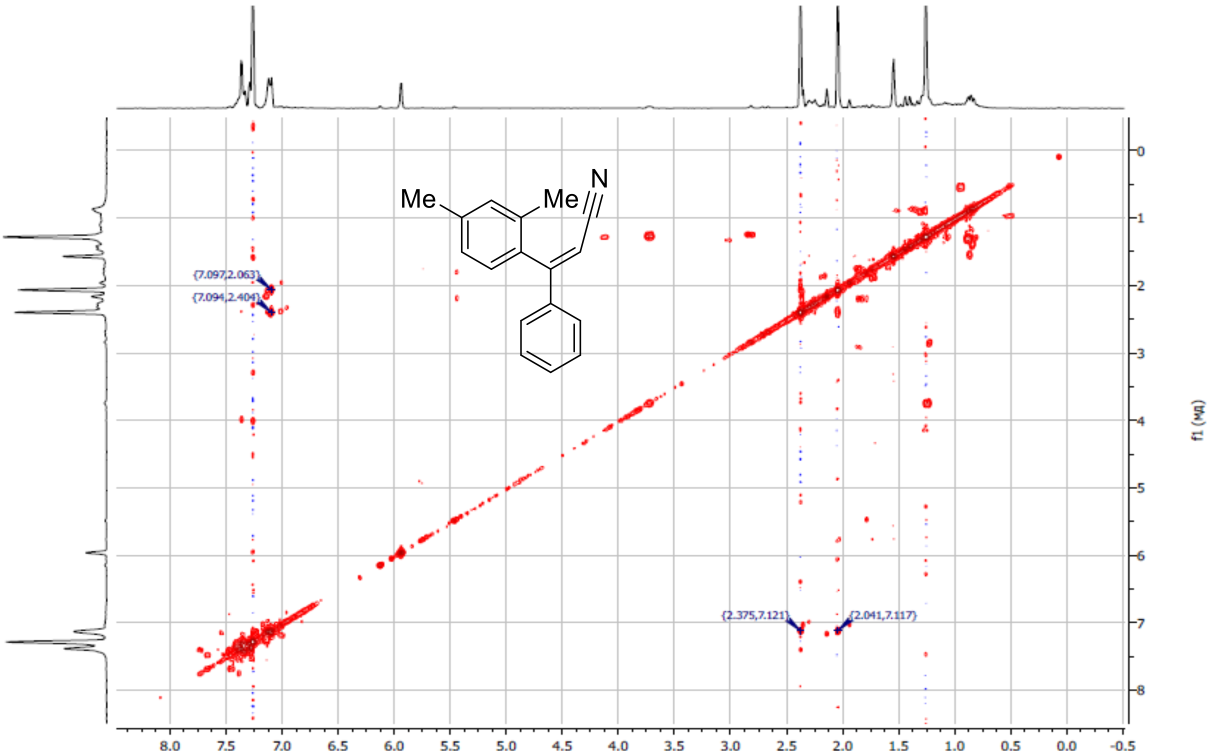

Figure S49. COSY NMR spectrum of compound **2i** ($CDCl_3$, 400 MHz).

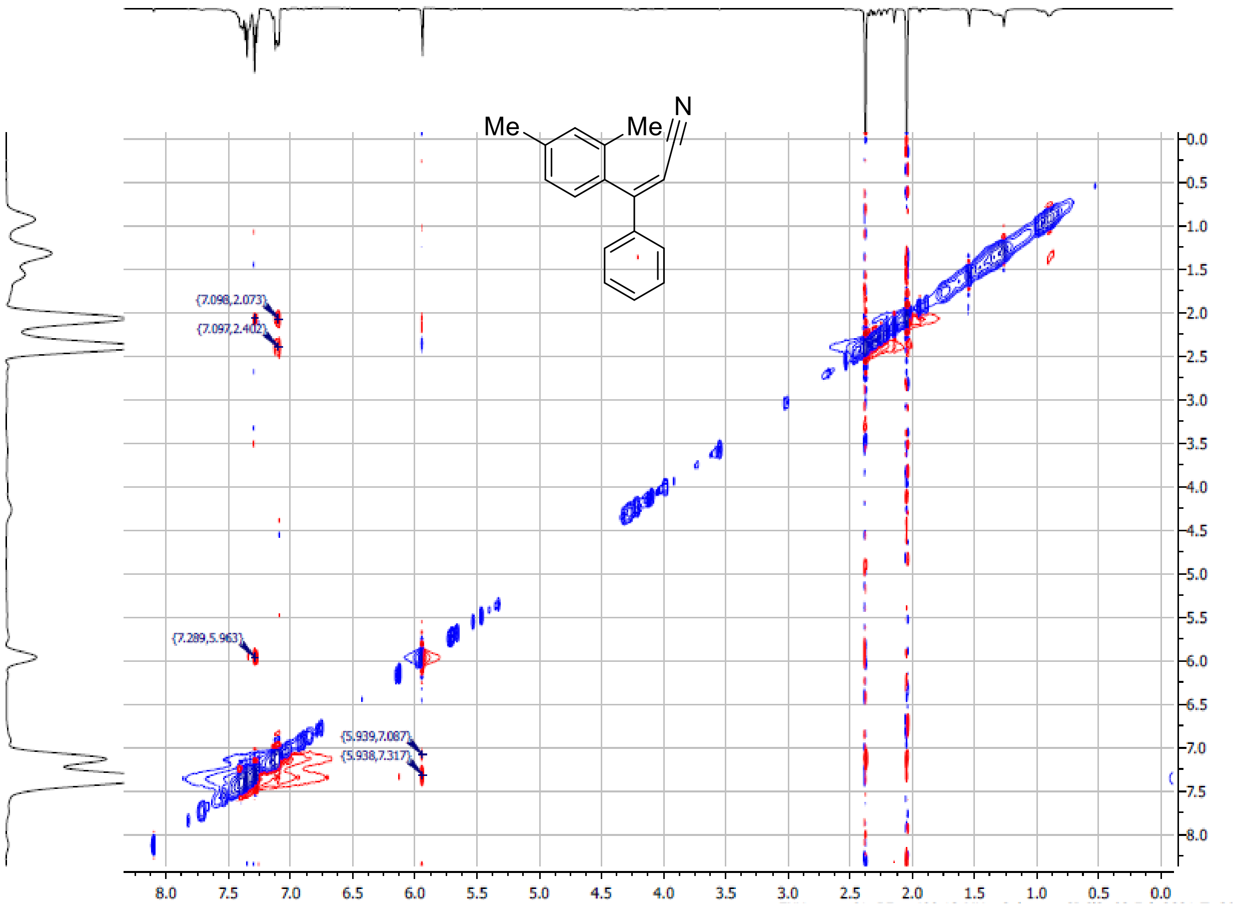

Figure S50. NOESY NMR spectrum of compound **2i** ($CDCl_3$, 400 MHz).

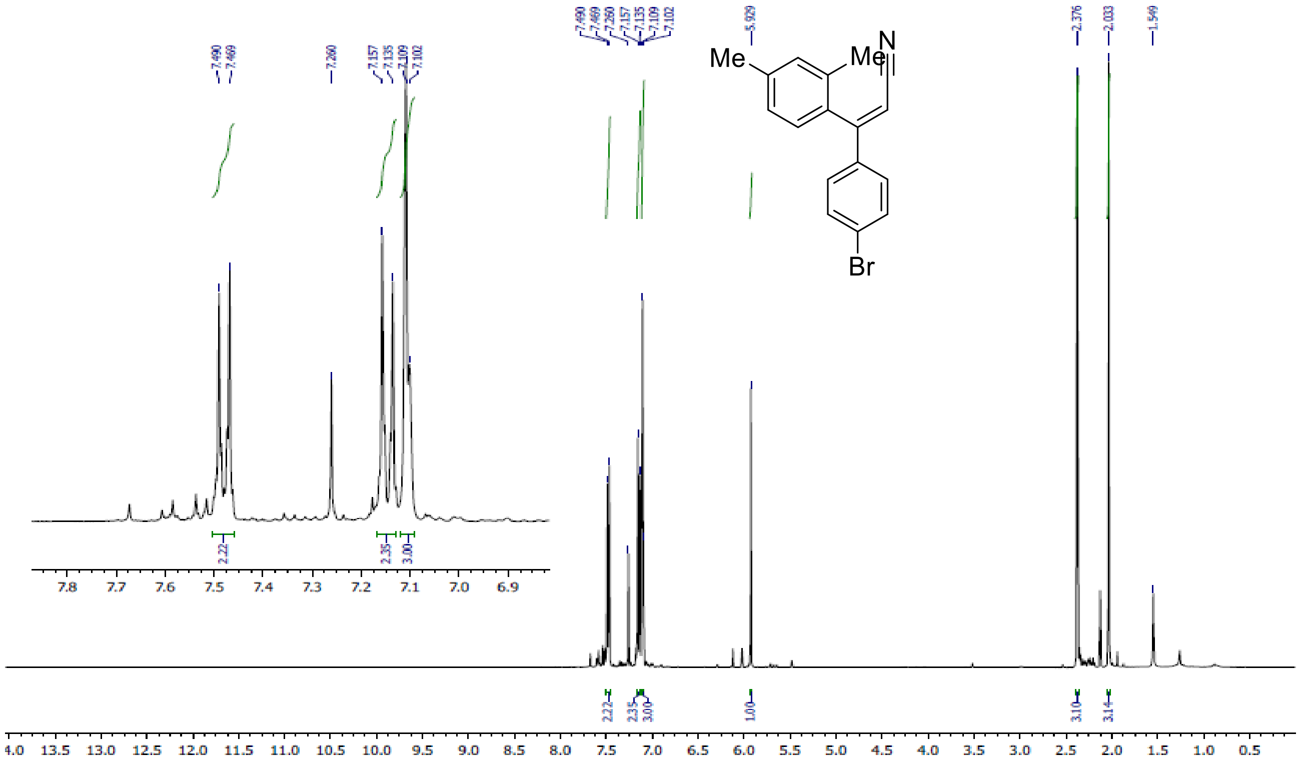

Figure S51. ^{1}H NMR spectrum of compound **2j** ($CDCl_3$, 400 MHz).

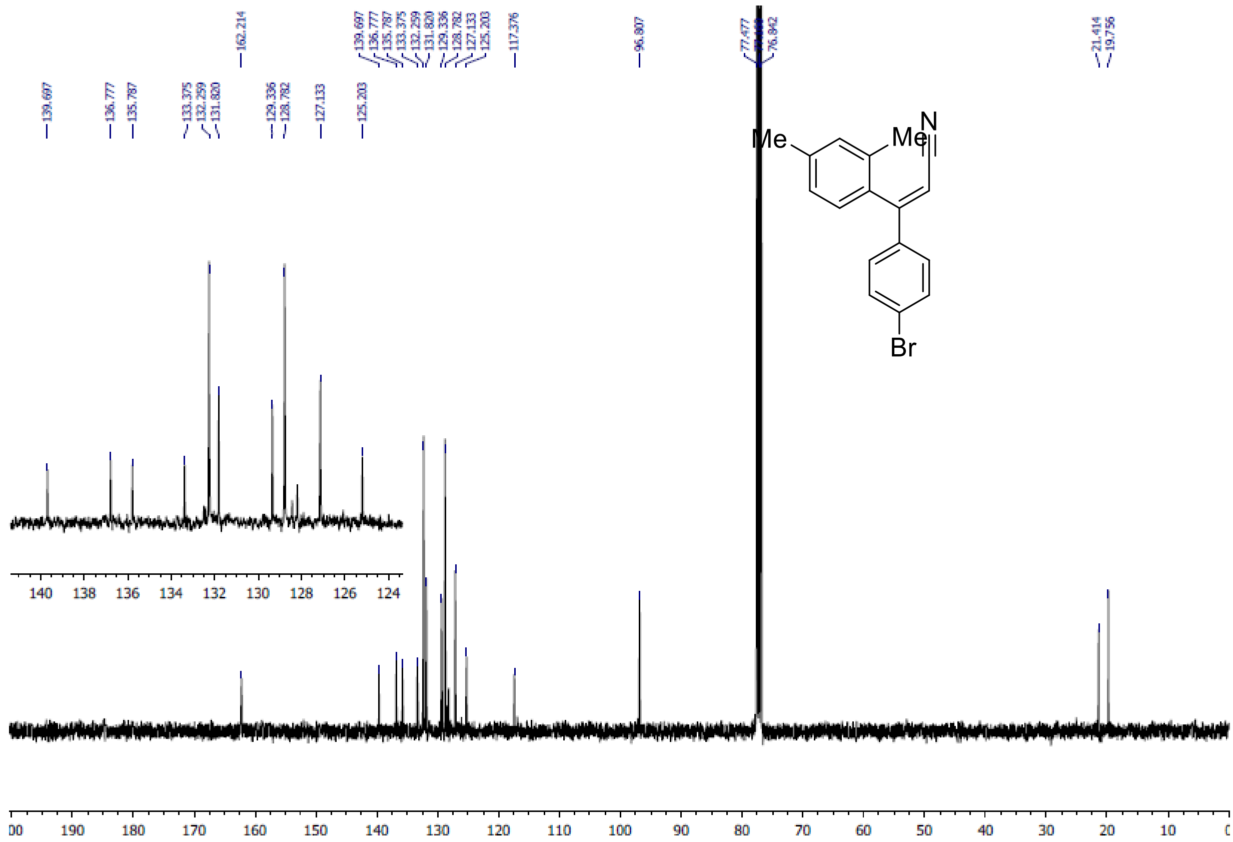

Figure S52. ^{13}C NMR spectrum of compound **2j** ($CDCl_3$, 100 MHz).

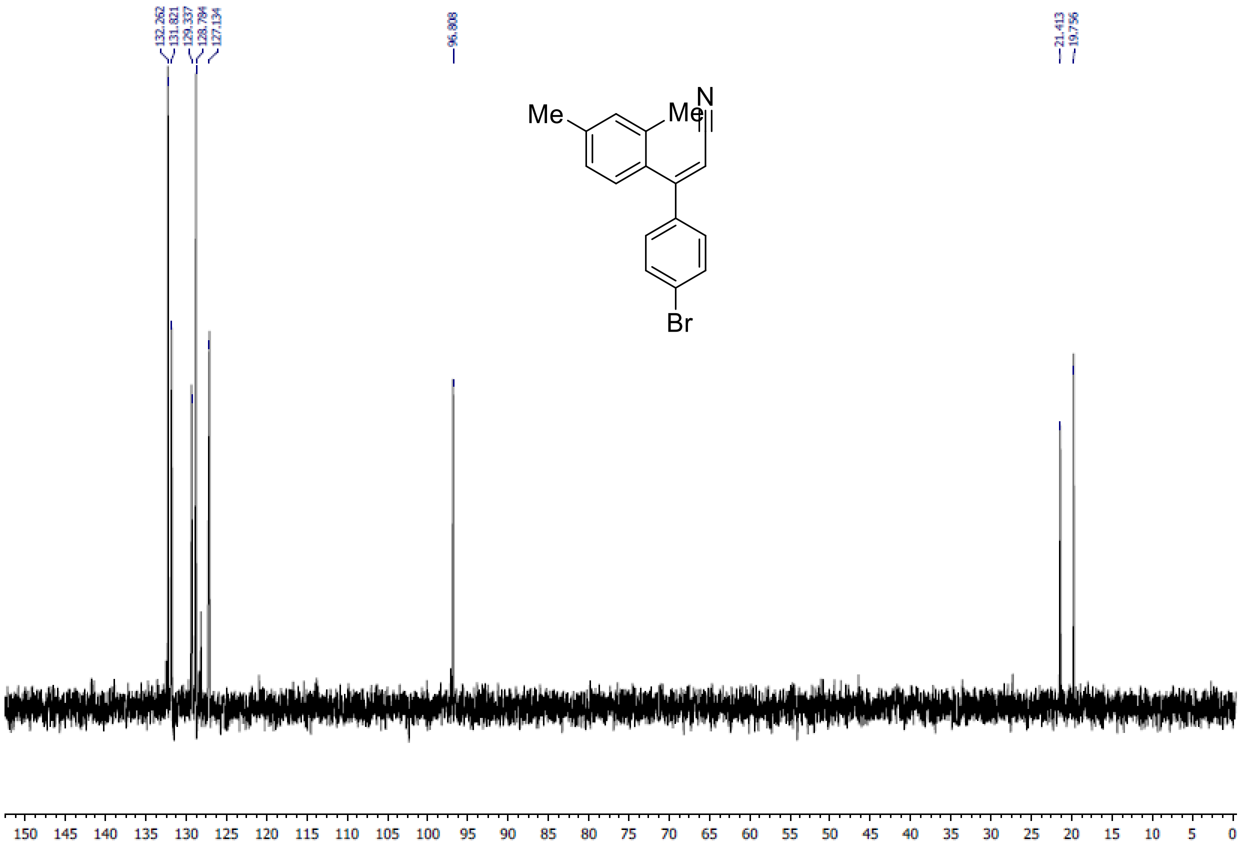

Figure S53. DEPT135 NMR spectrum of compound **2j** ($CDCl_3$, 400 MHz).

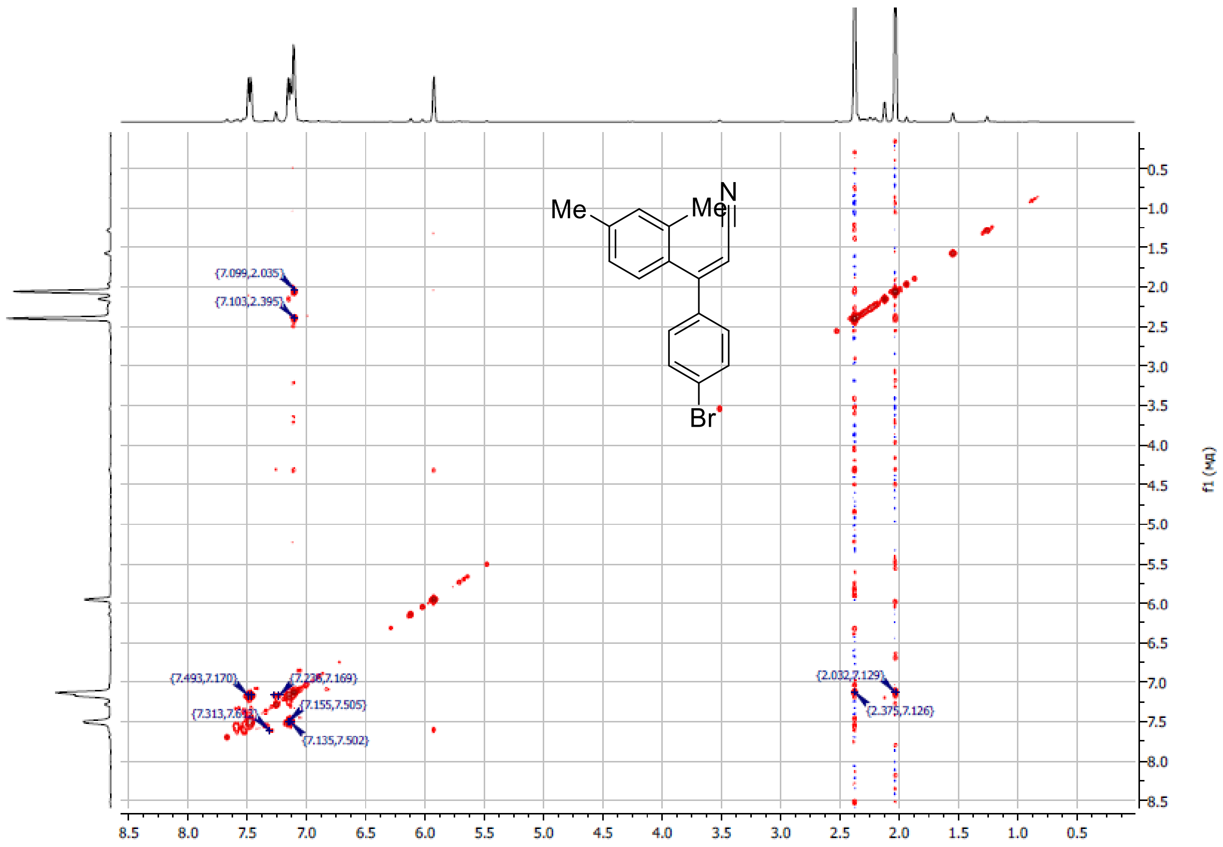

Figure S54. COSY NMR spectrum of compound **2j** ($CDCl_3$, 400 MHz).

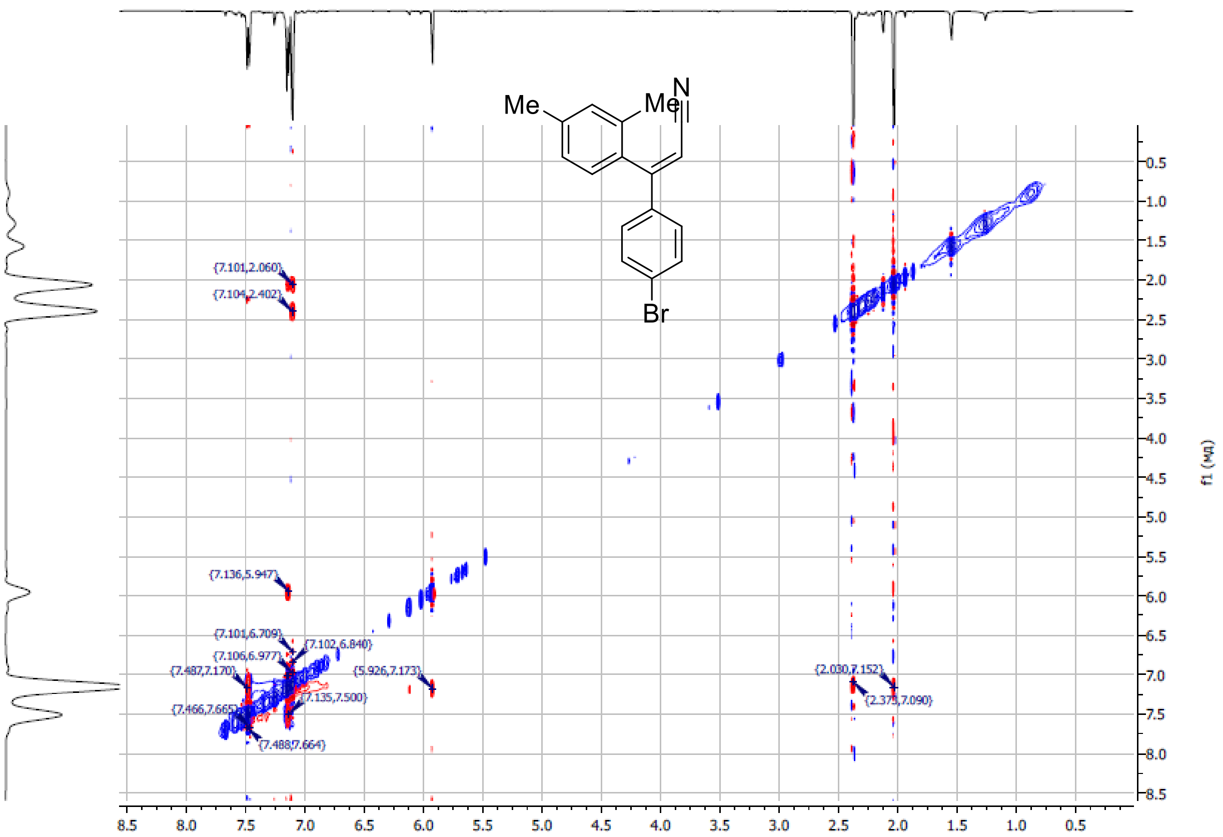

Figure S55. NOESY NMR spectrum of compound **2j** ($CDCl_3$, 400 MHz).

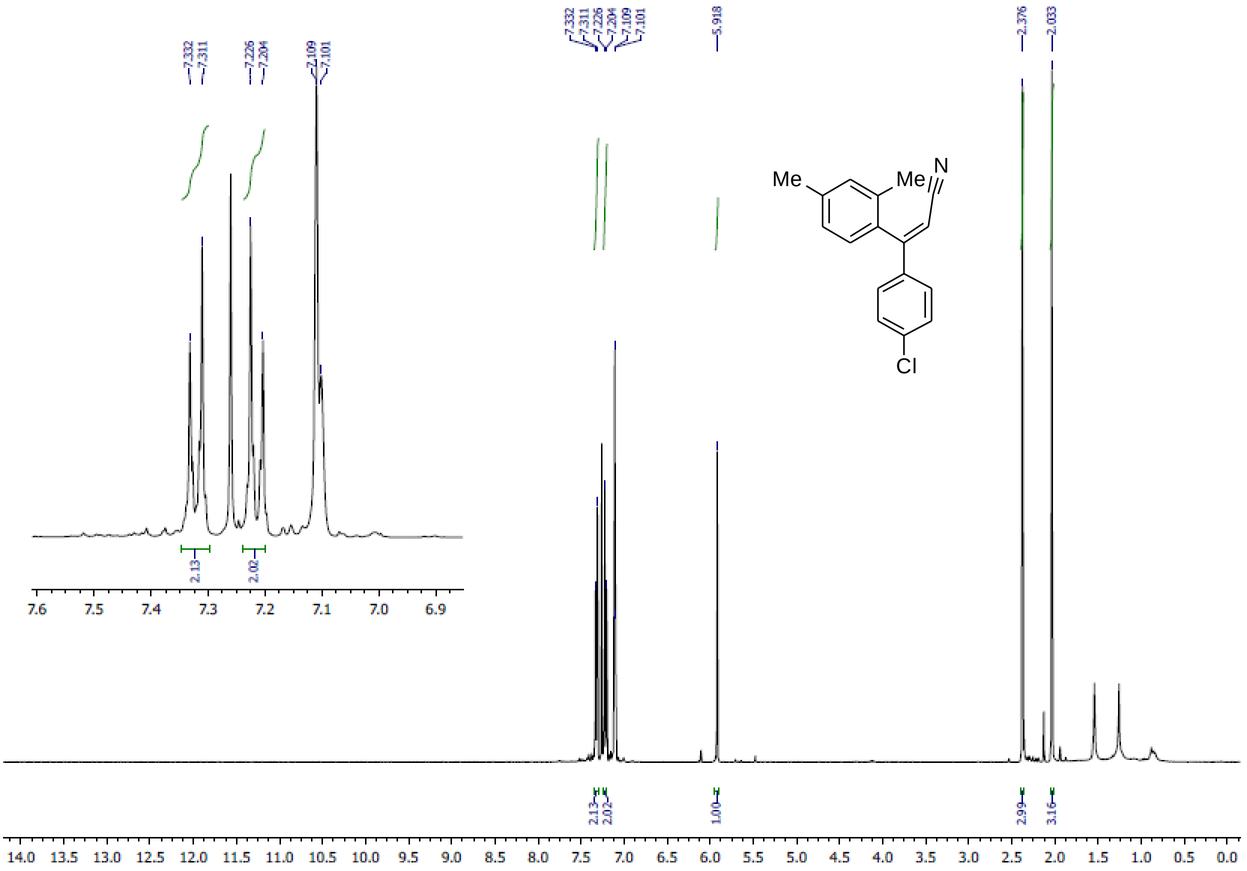

Figure S56. ^1H NMR spectrum of compound **2k** ($CDCl_3$, 400 MHz).

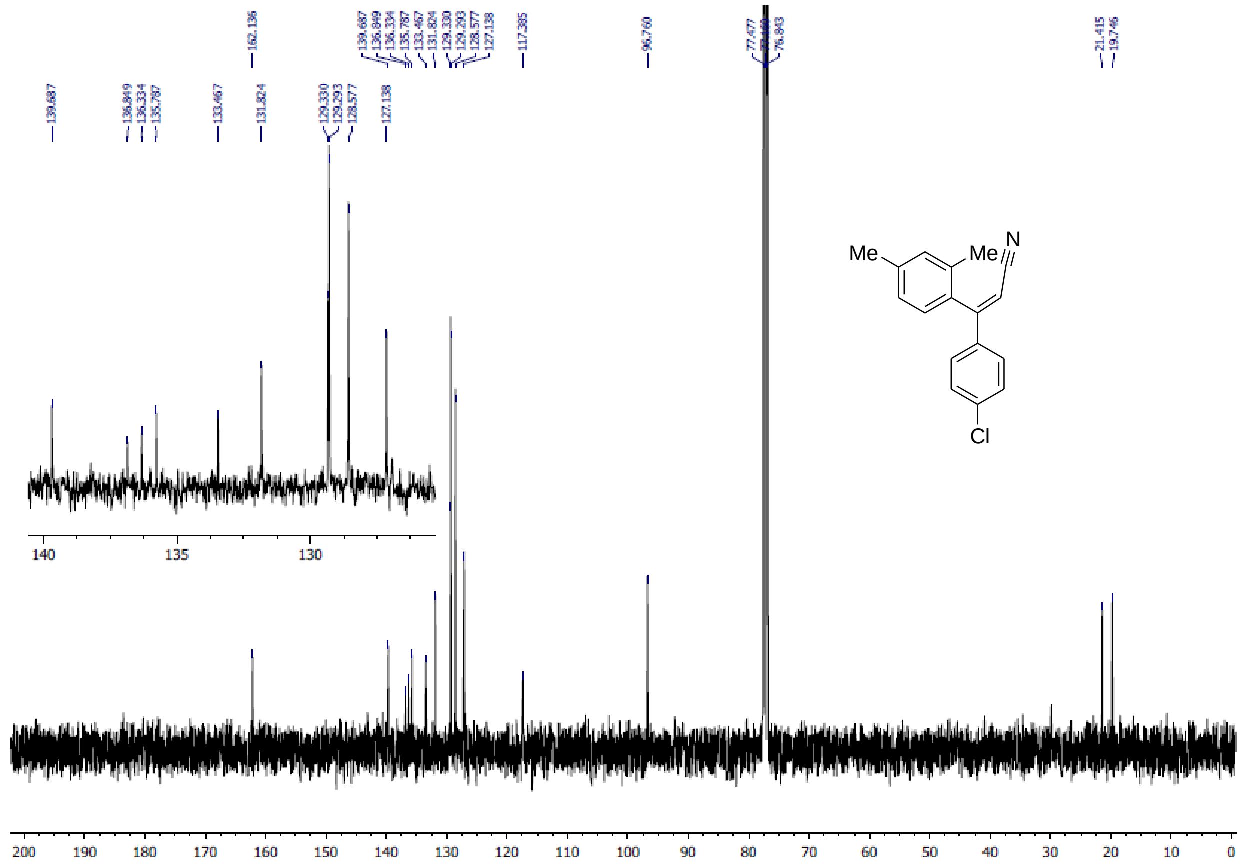

Figure S57. ^{13}C NMR spectrum of compound **2k** ($CDCl_3$, 100 MHz).

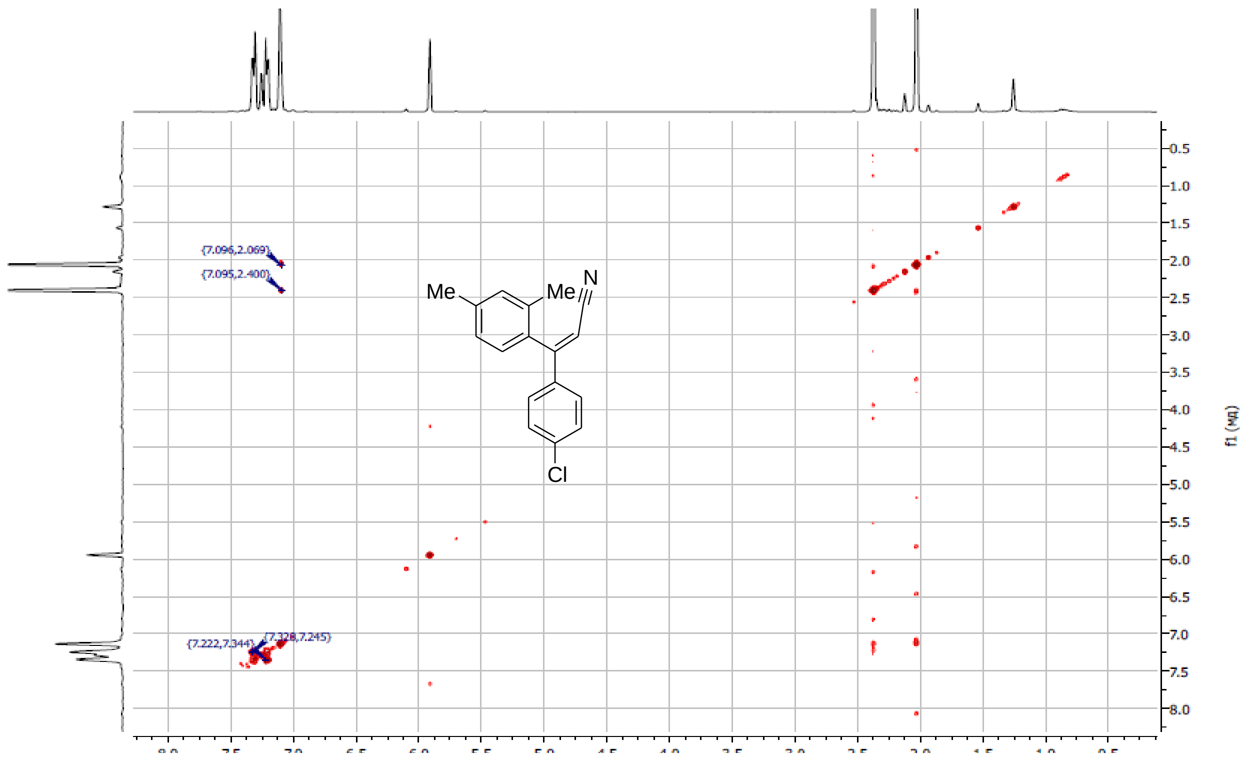

Figure S58. COSY NMR spectrum of compound **2k** ($CDCl_3$, 400 MHz).

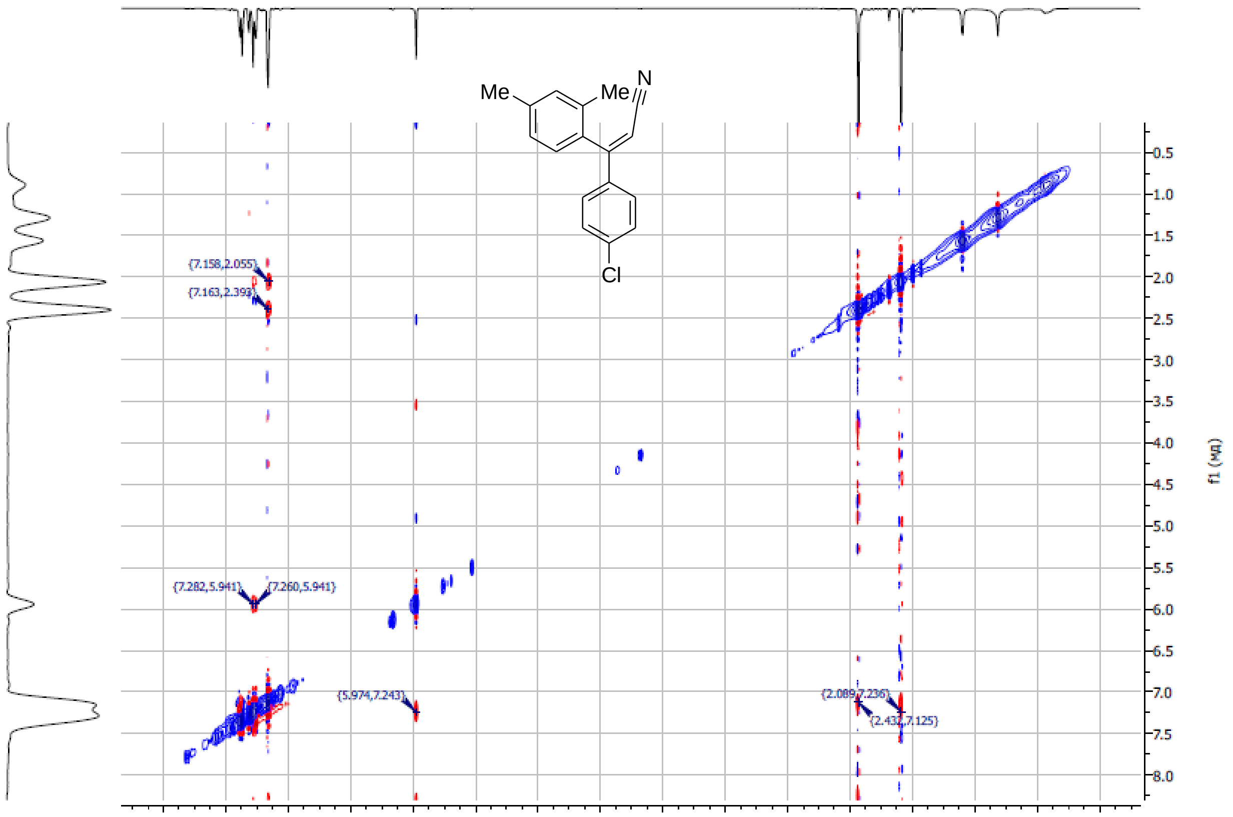

Figure S59. NOESY NMR spectrum of compound **2k** ($CDCl_3$, 400 MHz).

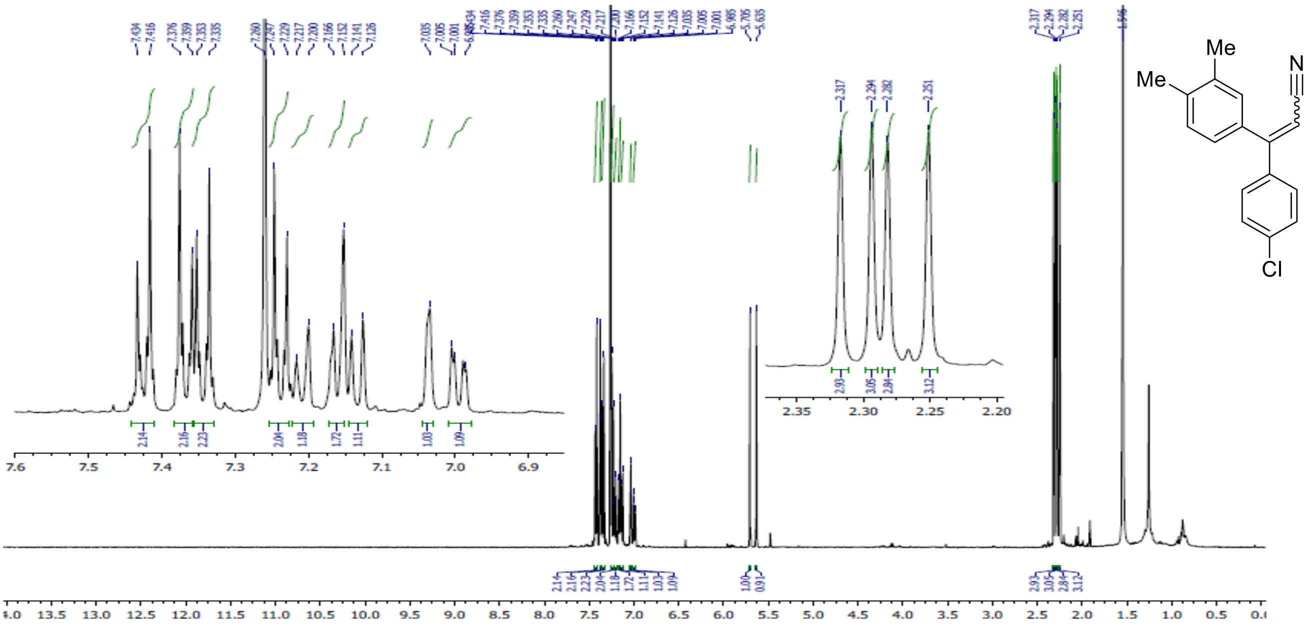

Figure S60. ^1H NMR spectrum of mixture of compounds *E*,*Z*-**2l** ($CDCl_3$, 400 MHz).

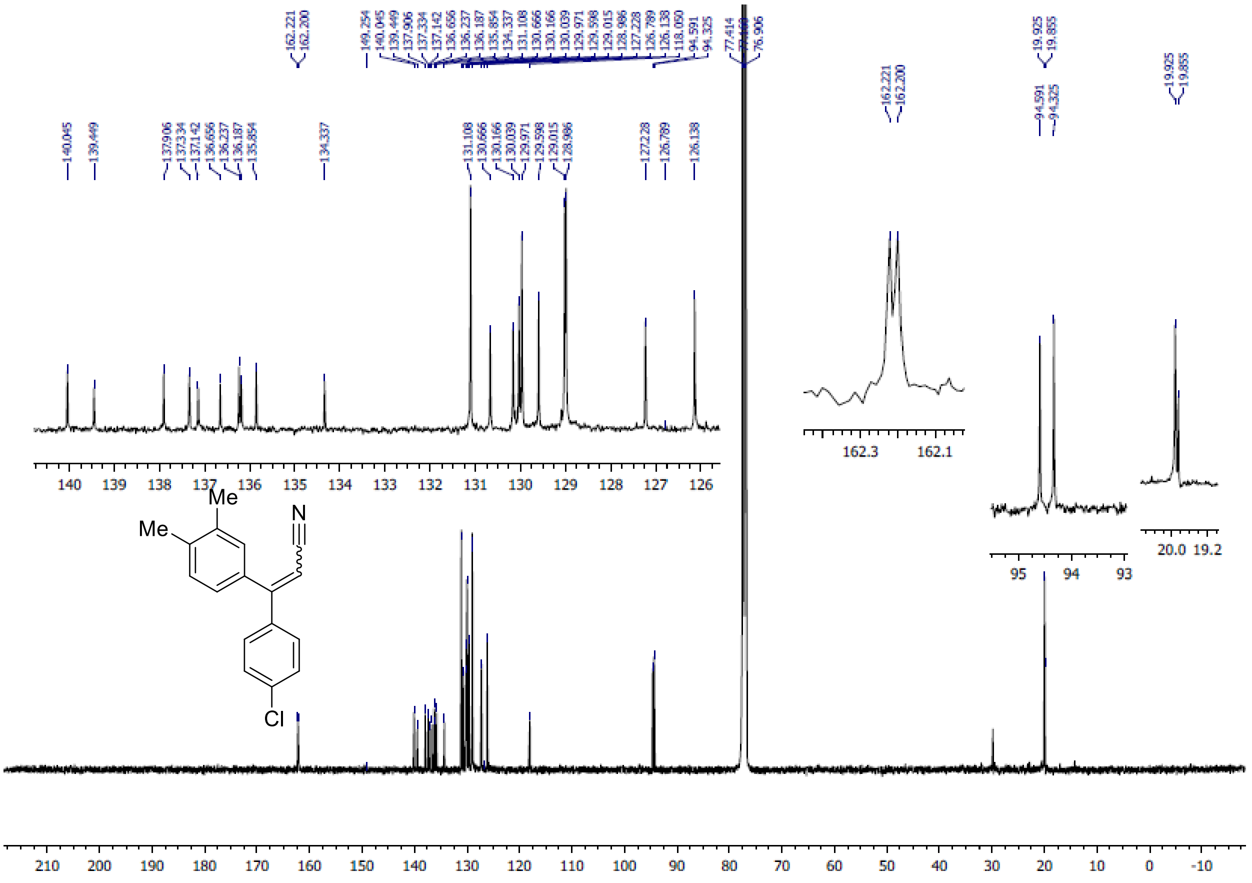

Figure S61. ^{13}C NMR spectrum of mixture of compounds *E*,*Z*-**2l** ($CDCl_3$, 100 MHz).

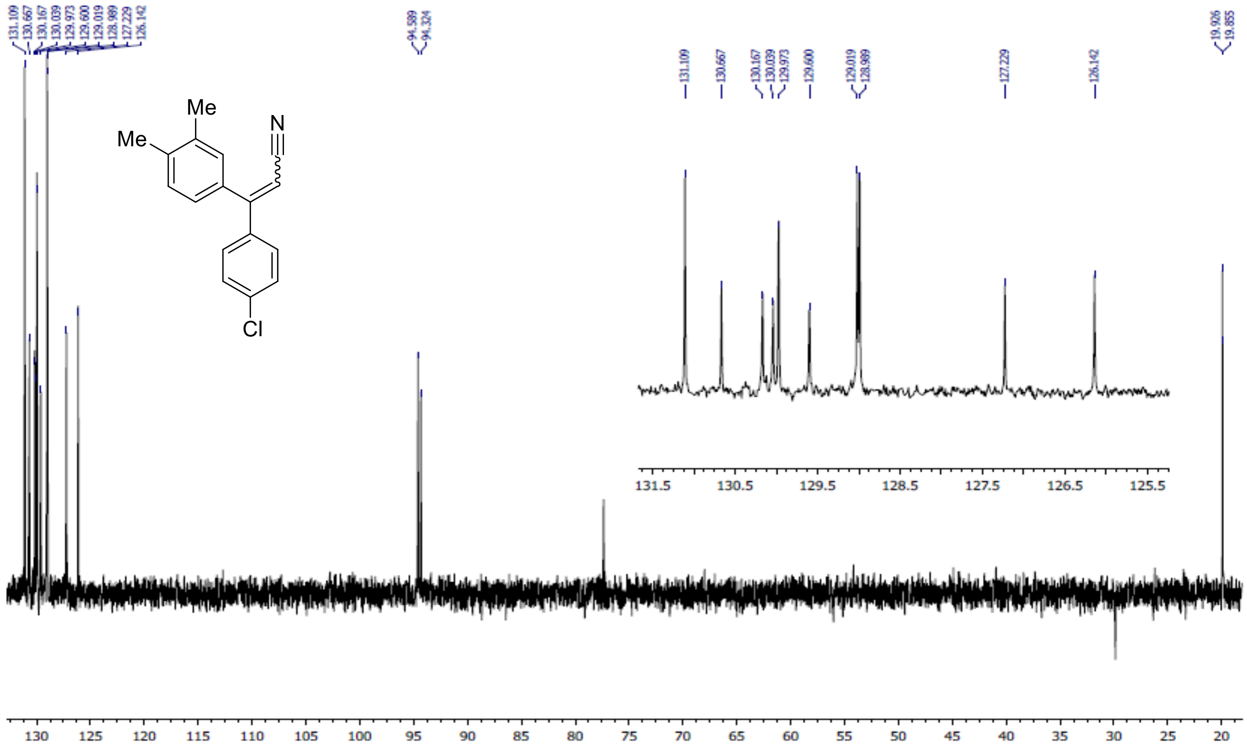

Figure S62. DEPT^{135} NMR spectrum of mixture of compounds *E*,*Z*-**2l** ($CDCl_3$, 400 MHz).

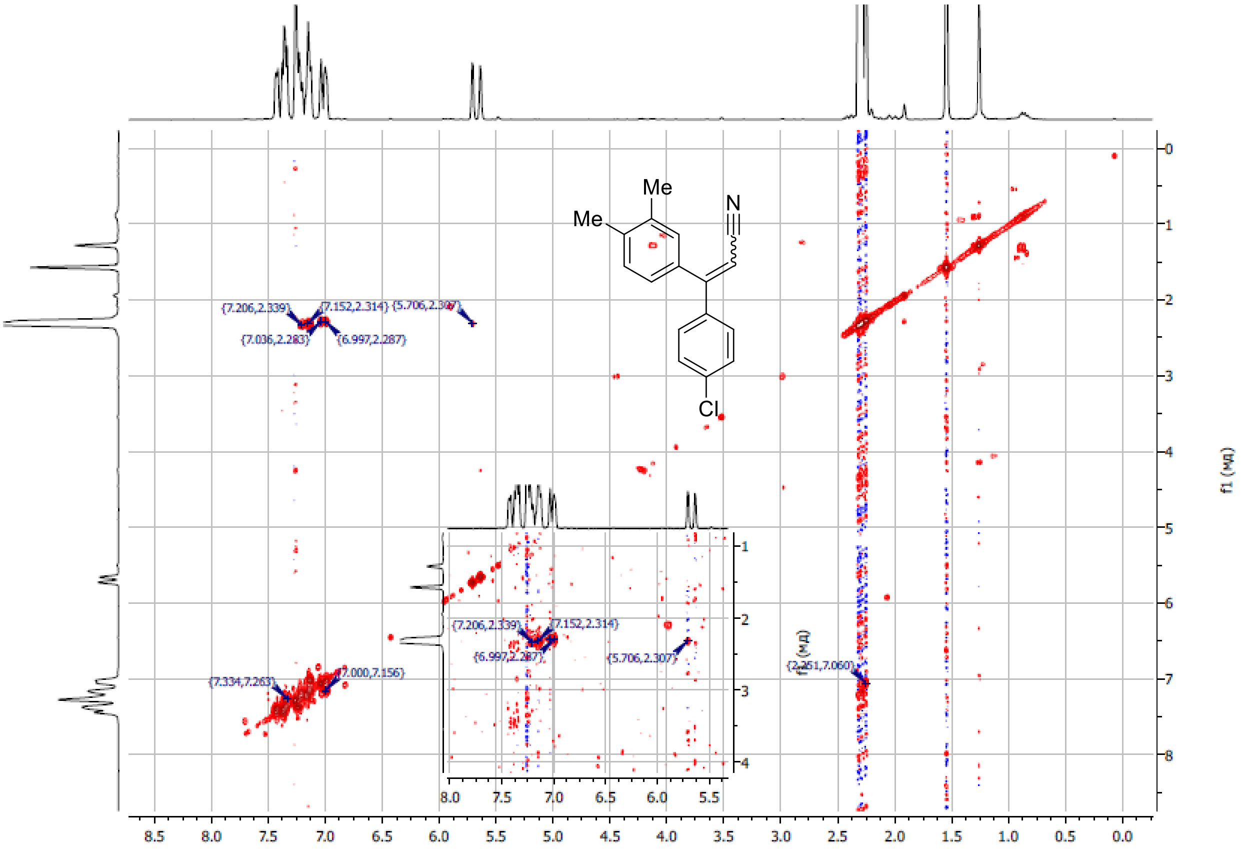

Figure S63. COSY NMR spectrum of mixture of compounds *E*,*Z*-**2l** ($CDCl_3$, 400 MHz).

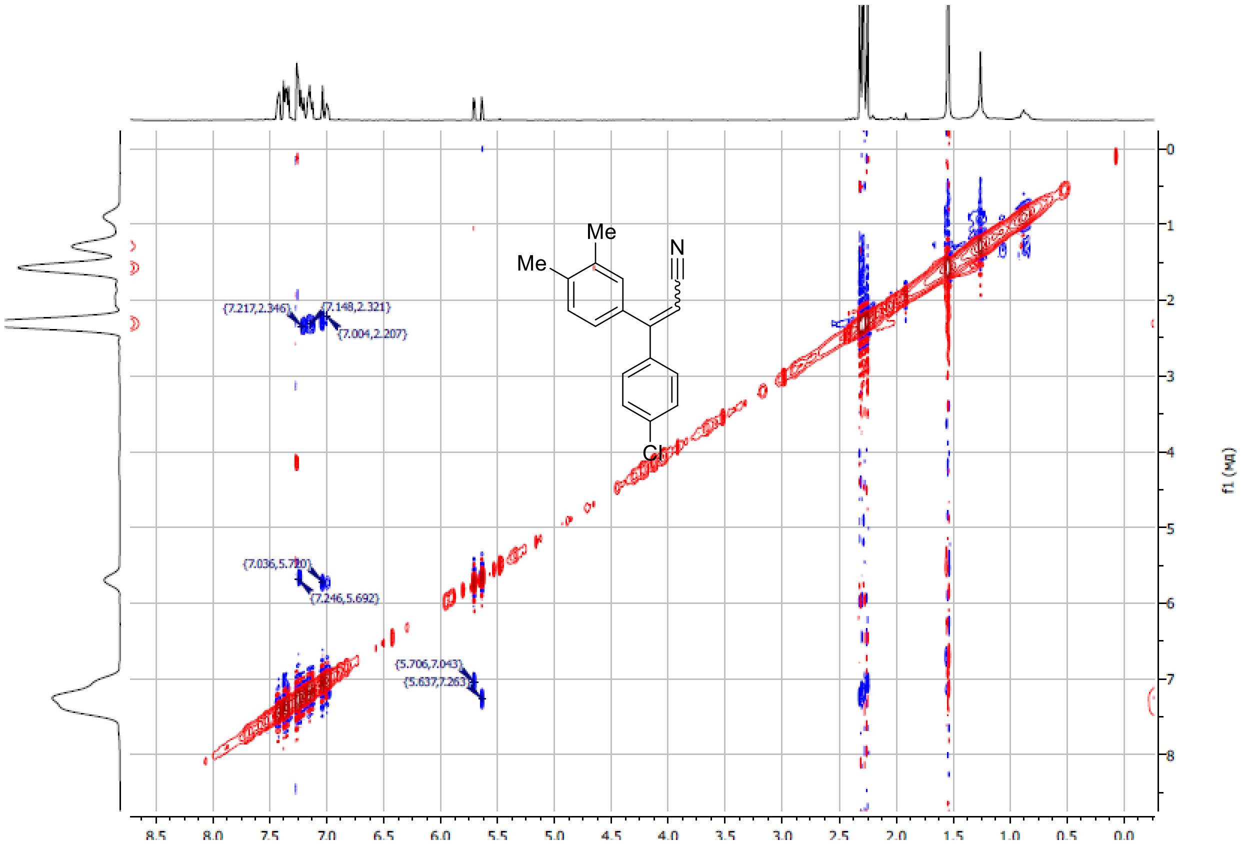

Figure S64. NOESY NMR spectrum of mixture of compounds *E*,*Z*-**2l** ($CDCl_3$, 400 MHz).

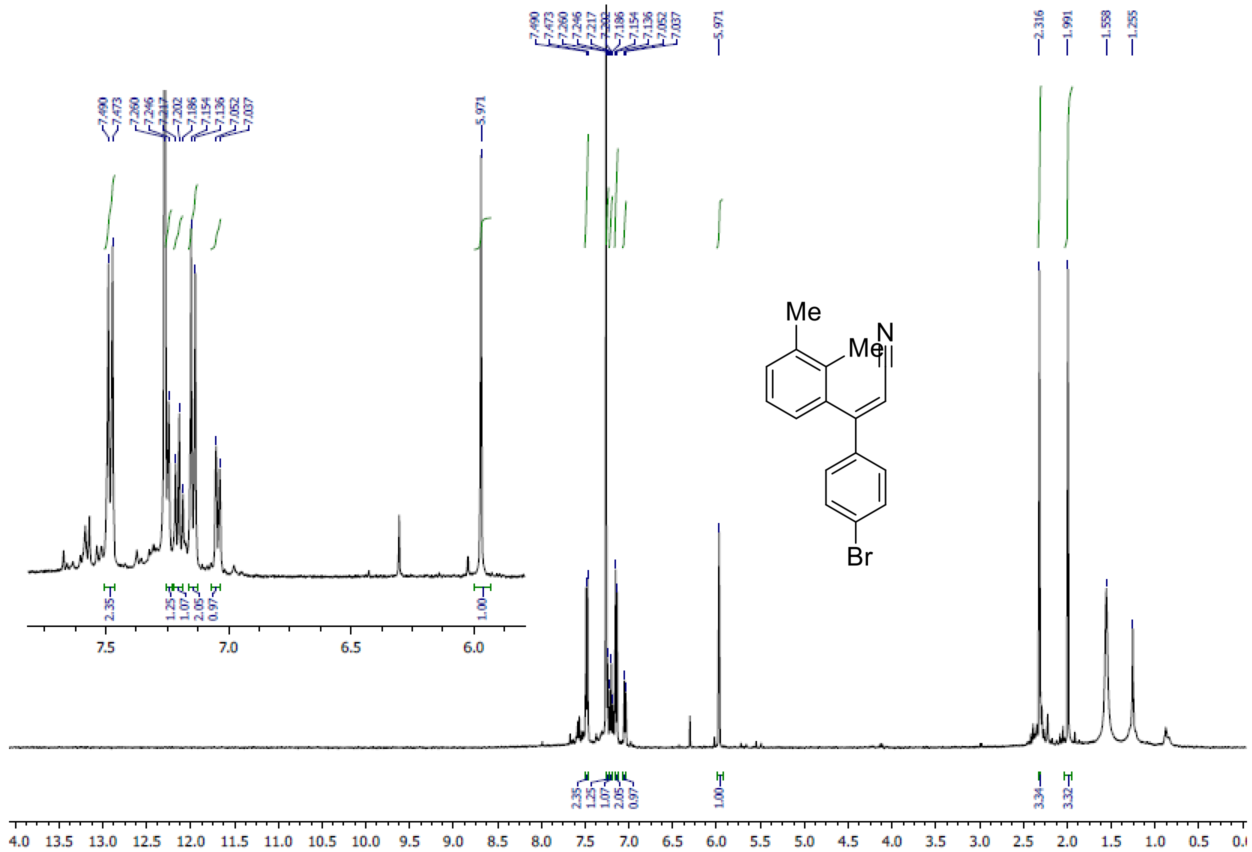

Figure S65. ^{1}H NMR spectrum of compound **2m** ($CDCl_3$, 400 MHz).

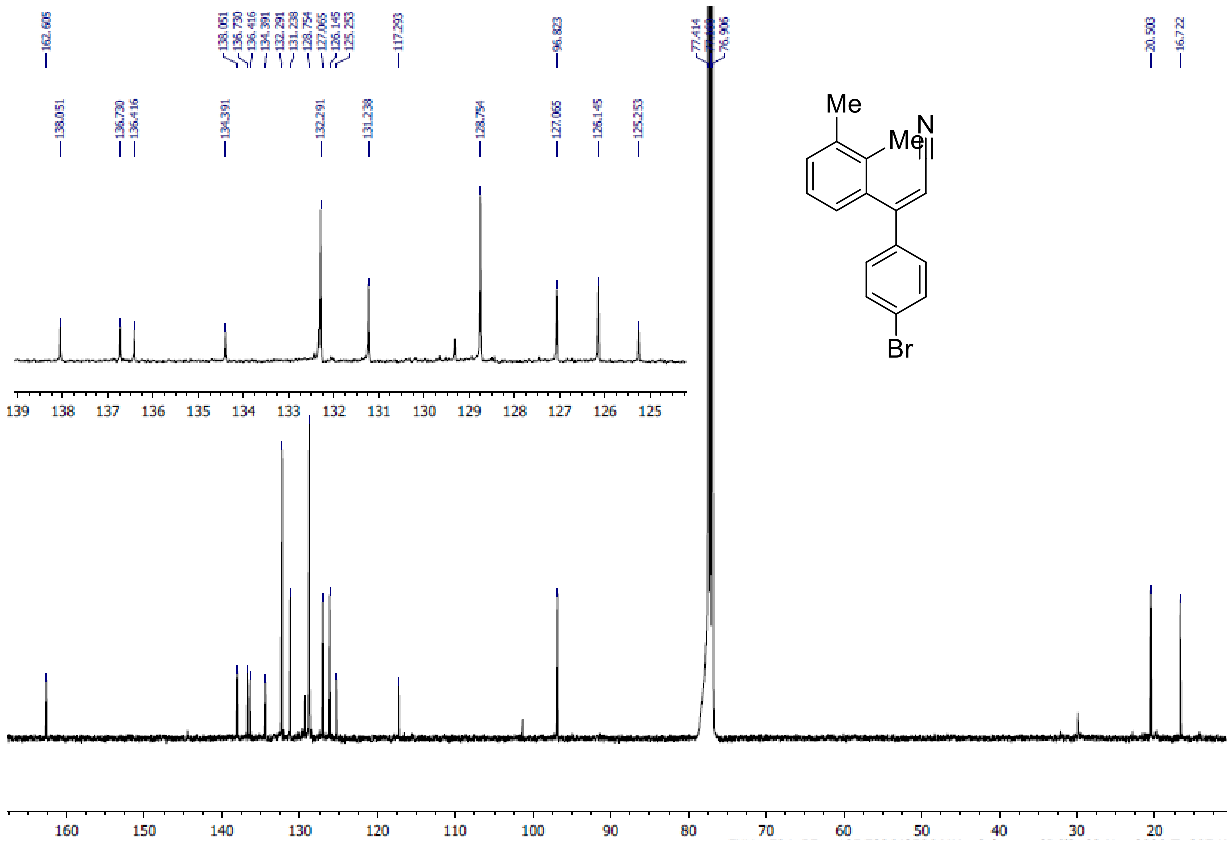

Figure S66. ^{13}C NMR spectrum of compound **2m** ($CDCl_3$, 100 MHz).

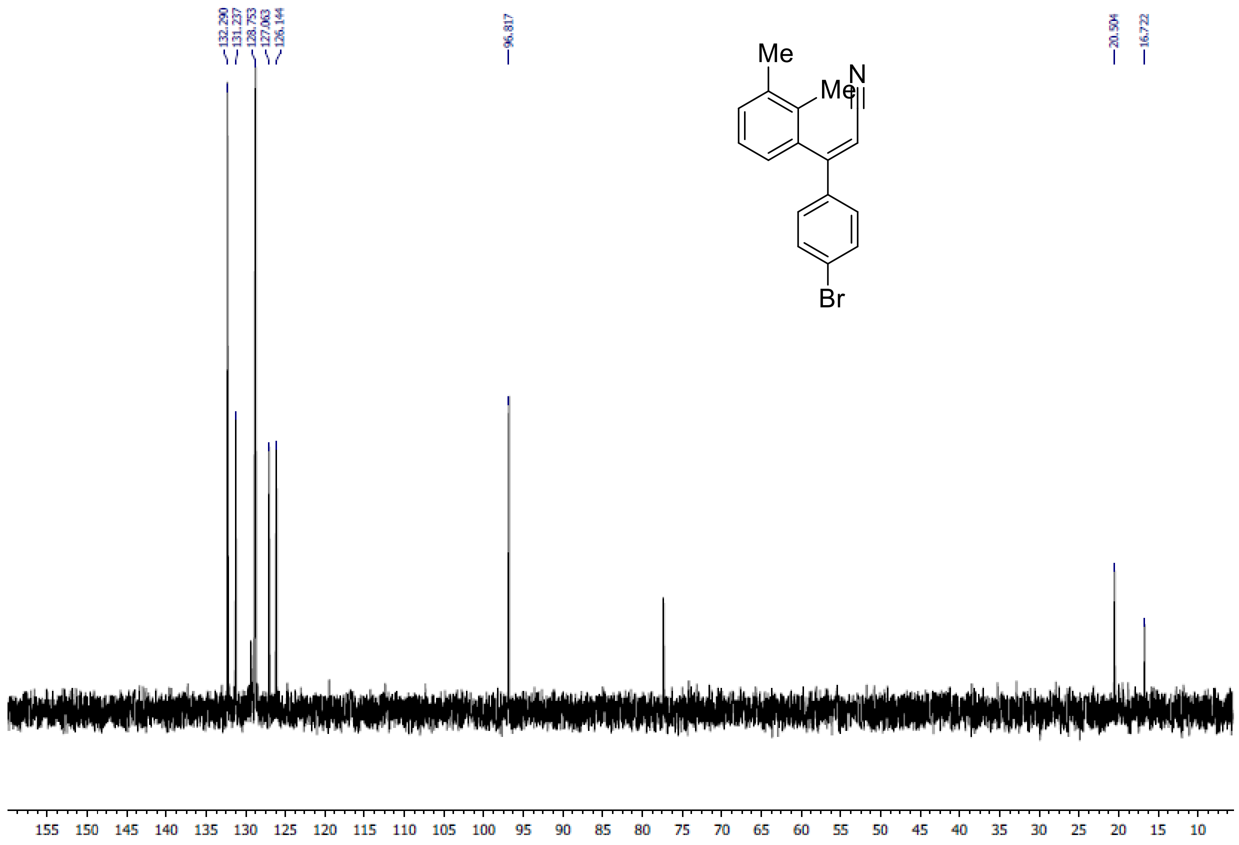

Figure S67. DEPT[135] NMR spectrum of compound **2m** ($CDCl_3$, 400 MHz).

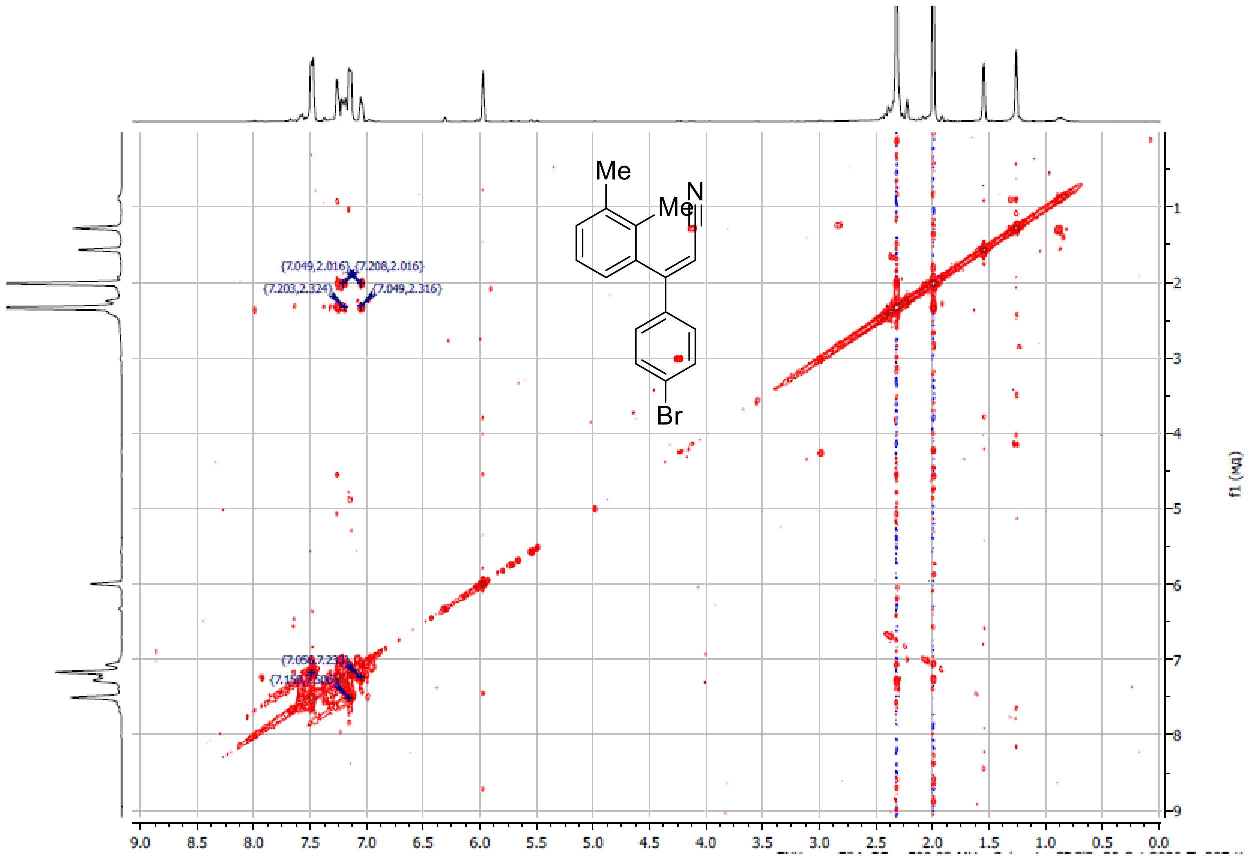

Figure S68. COSY NMR spectrum of compound **2m** ($CDCl_3$, 400 MHz).

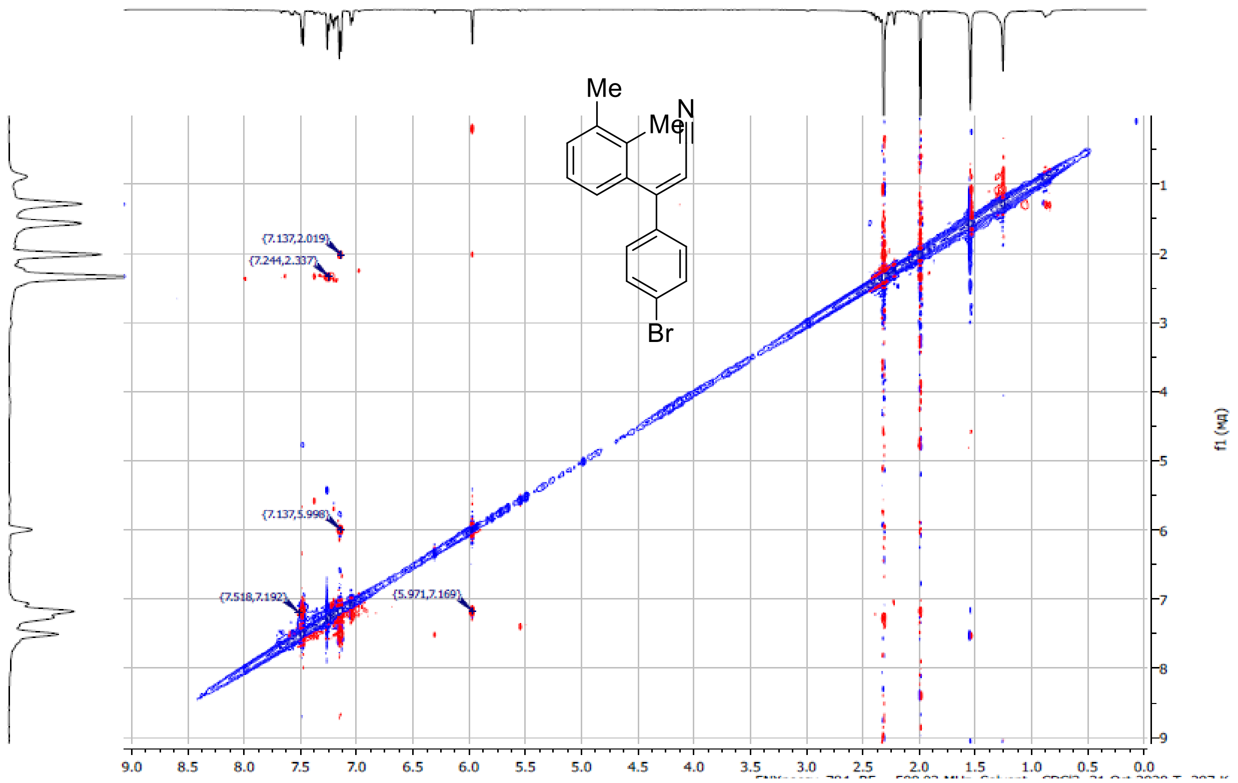

Figure S69. NOESY NMR spectrum of compound **2m** ($CDCl_3$, 400 MHz).

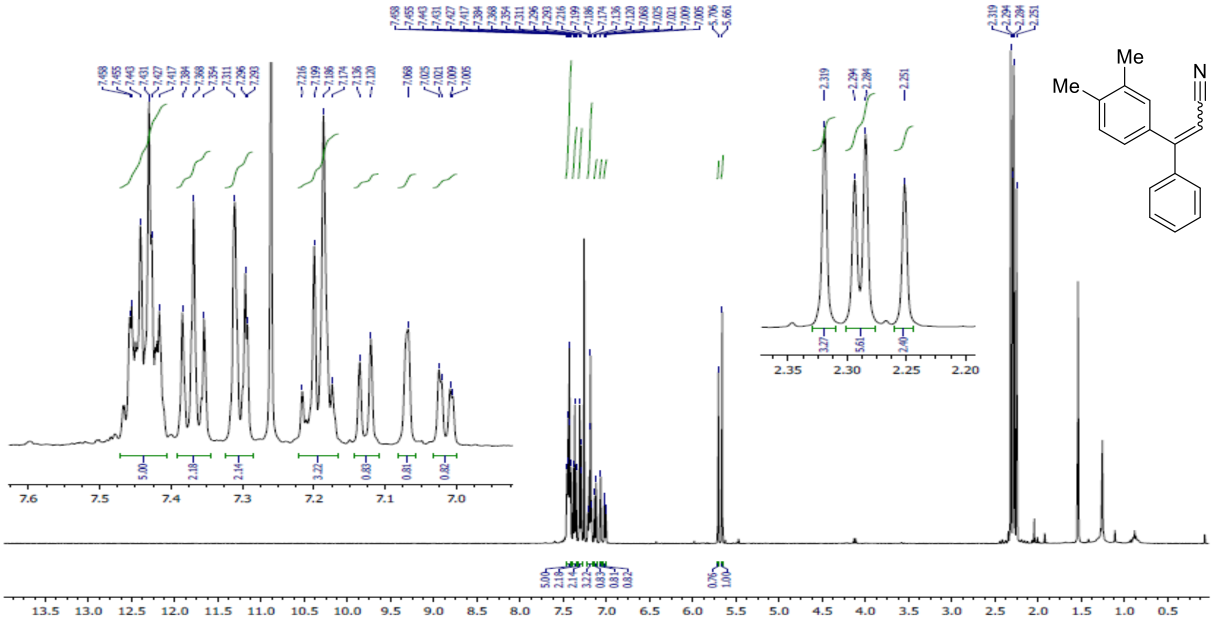

Figure S70. ^1H NMR spectrum of mixture of compounds *E*,*Z*-**2n** ($CDCl_3$, 400 MHz).

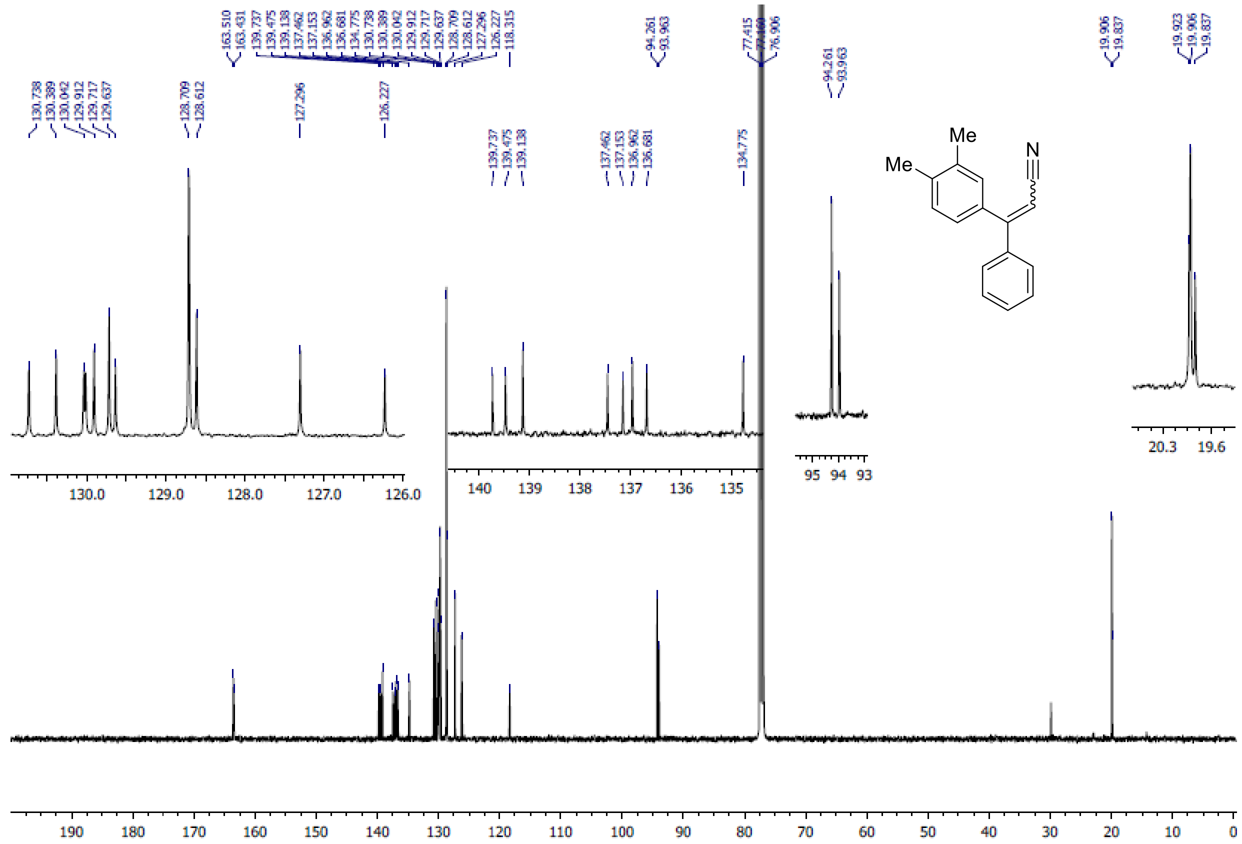

Figure S71. ^{13}C NMR spectrum of mixture of compounds *E*,*Z*-**2n** ($CDCl_3$, 100 MHz).

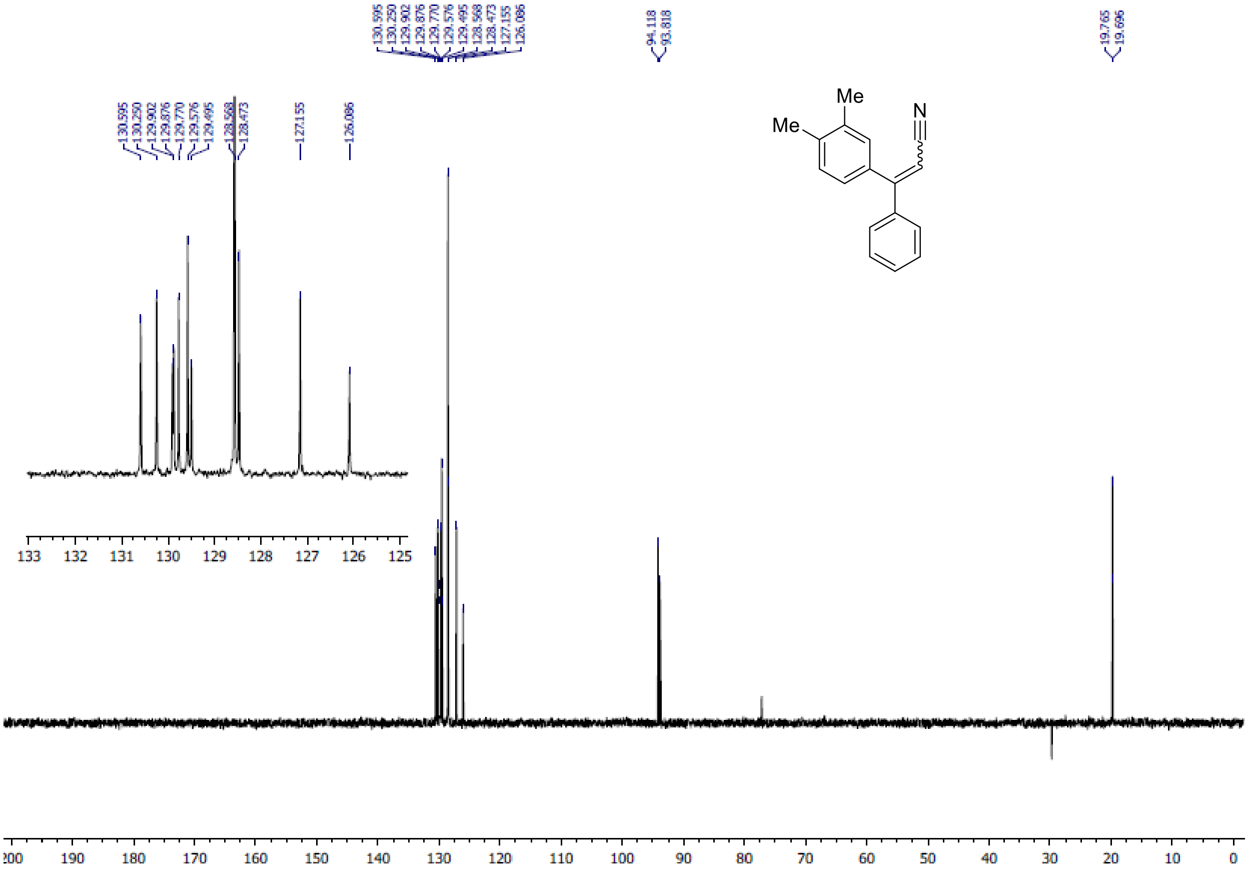

Figure S72. DEPT^{135} NMR spectrum of mixture of compounds *E*,*Z*-**2n** ($CDCl_3$, 400 MHz).

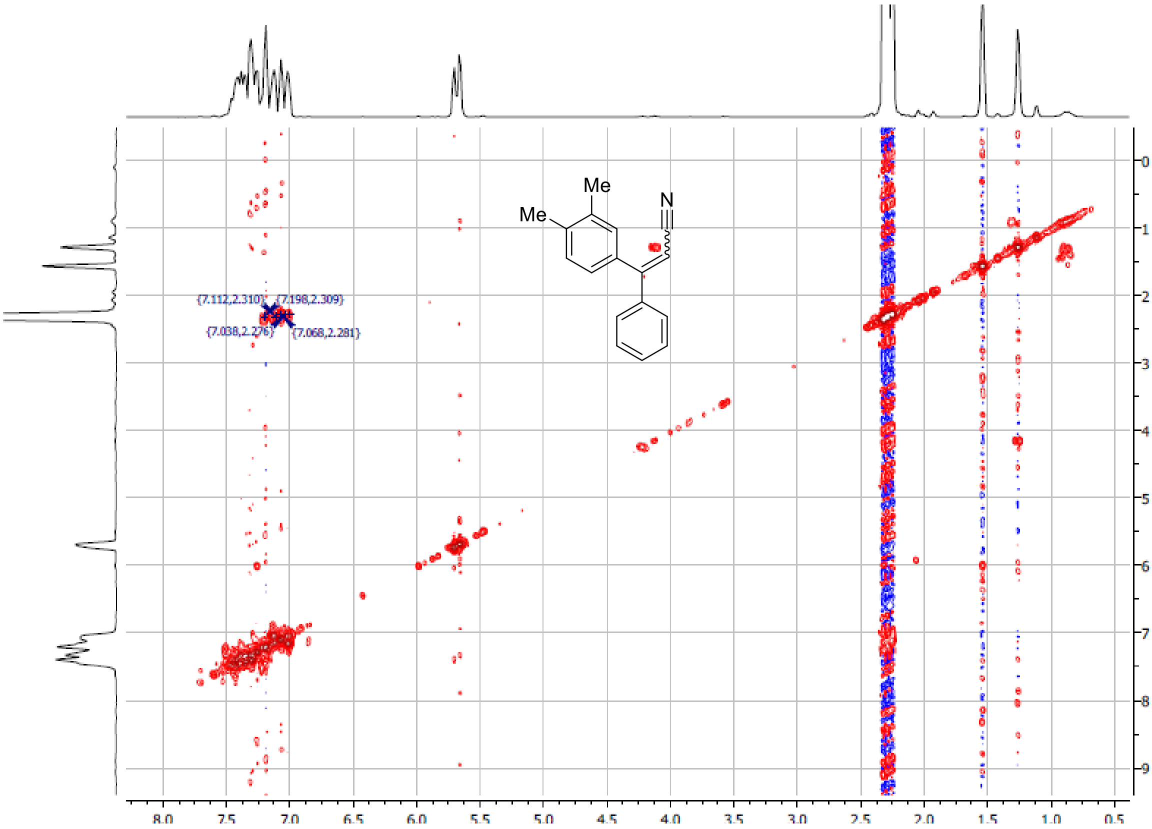

Figure S73. COSY NMR spectrum of mixture of compounds *E*,*Z*-**2n** ($CDCl_3$, 400 MHz).

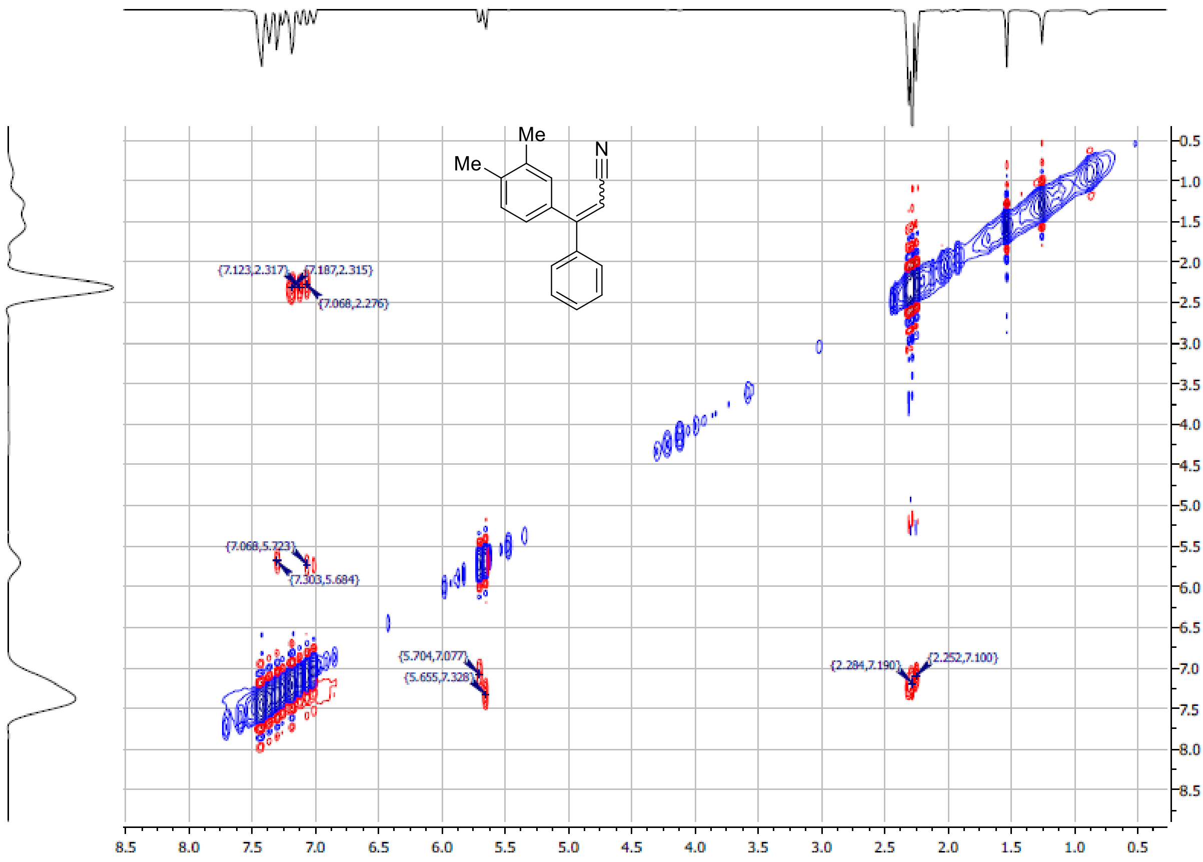

Figure S74. NOESY NMR spectrum of mixture of compounds *E*,*Z*-**2n** ($CDCl_3$, 400 MHz).

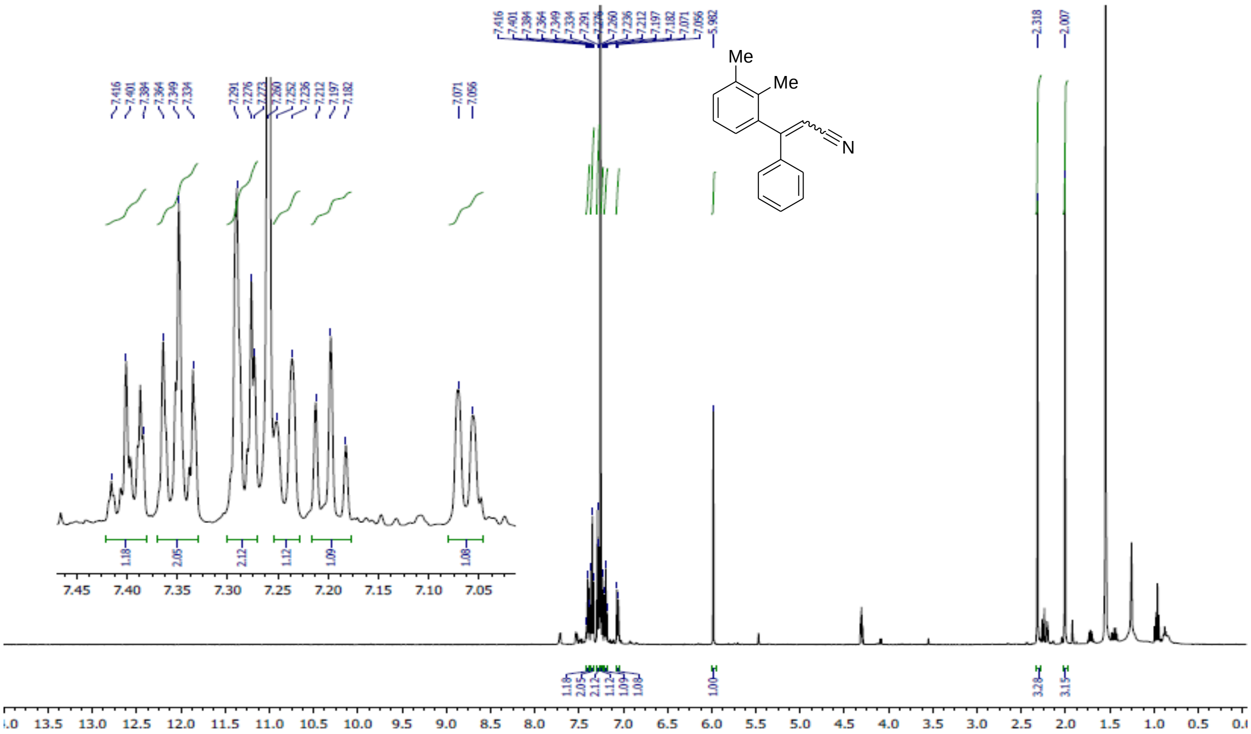

Figure S75. ^{1}H NMR spectrum of compound **2o** ($CDCl_3$, 400 MHz).

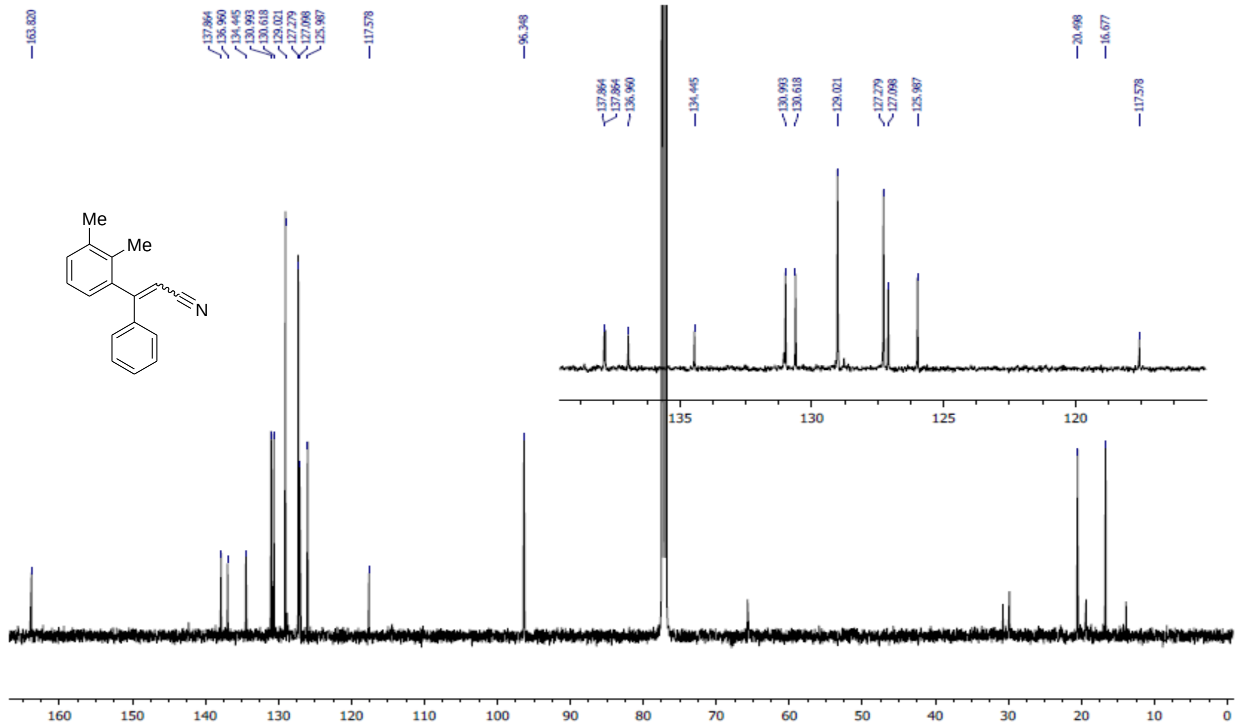

Figure S76. ^{13}C NMR spectrum of compound **2o** ($CDCl_3$, 100 MHz).

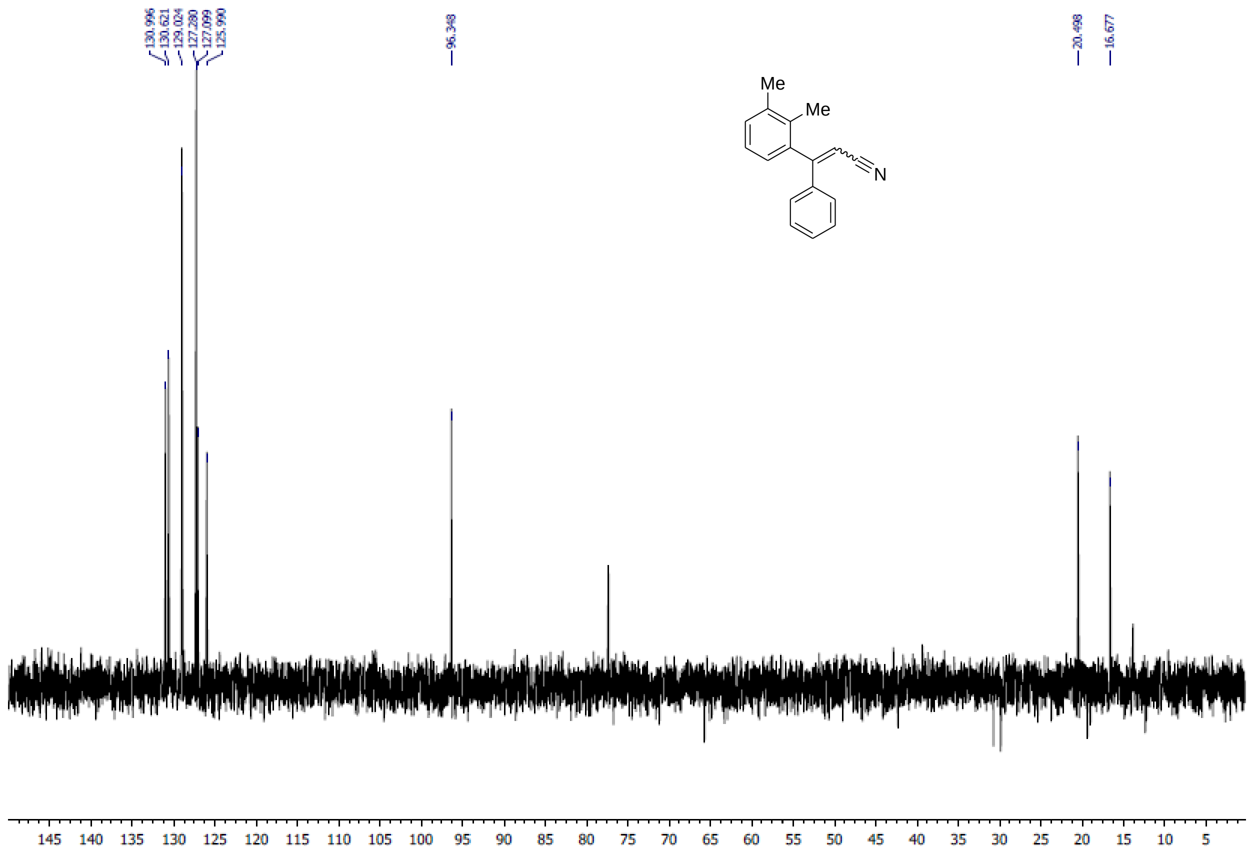

Figure S77. DEPT[135] NMR spectrum of compound **2o** ($CDCl_3$, 400 MHz).

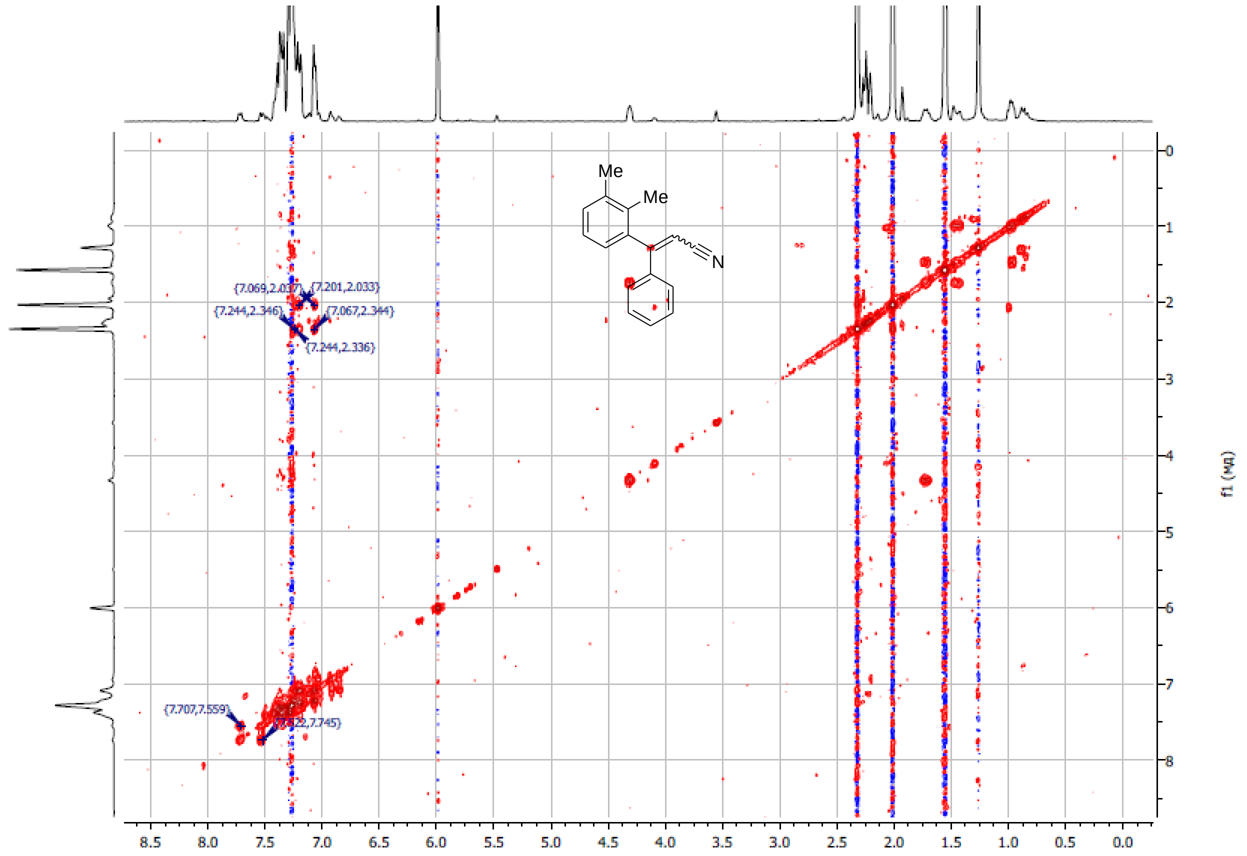

Figure S78. COSY NMR spectrum of compound **2o** ($CDCl_3$, 400 MHz).

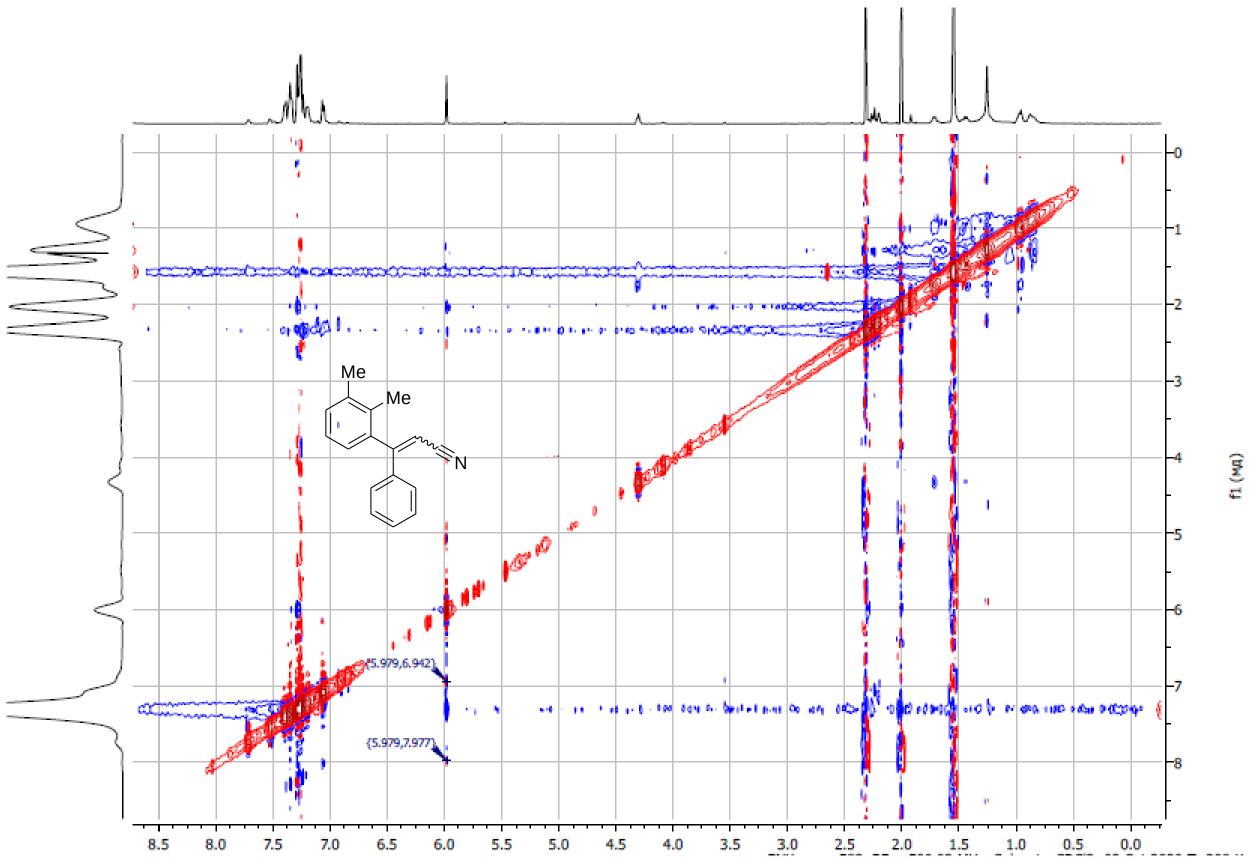

Figure S79. NOESY NMR spectrum of compound **2o** ($CDCl_3$, 400 MHz).

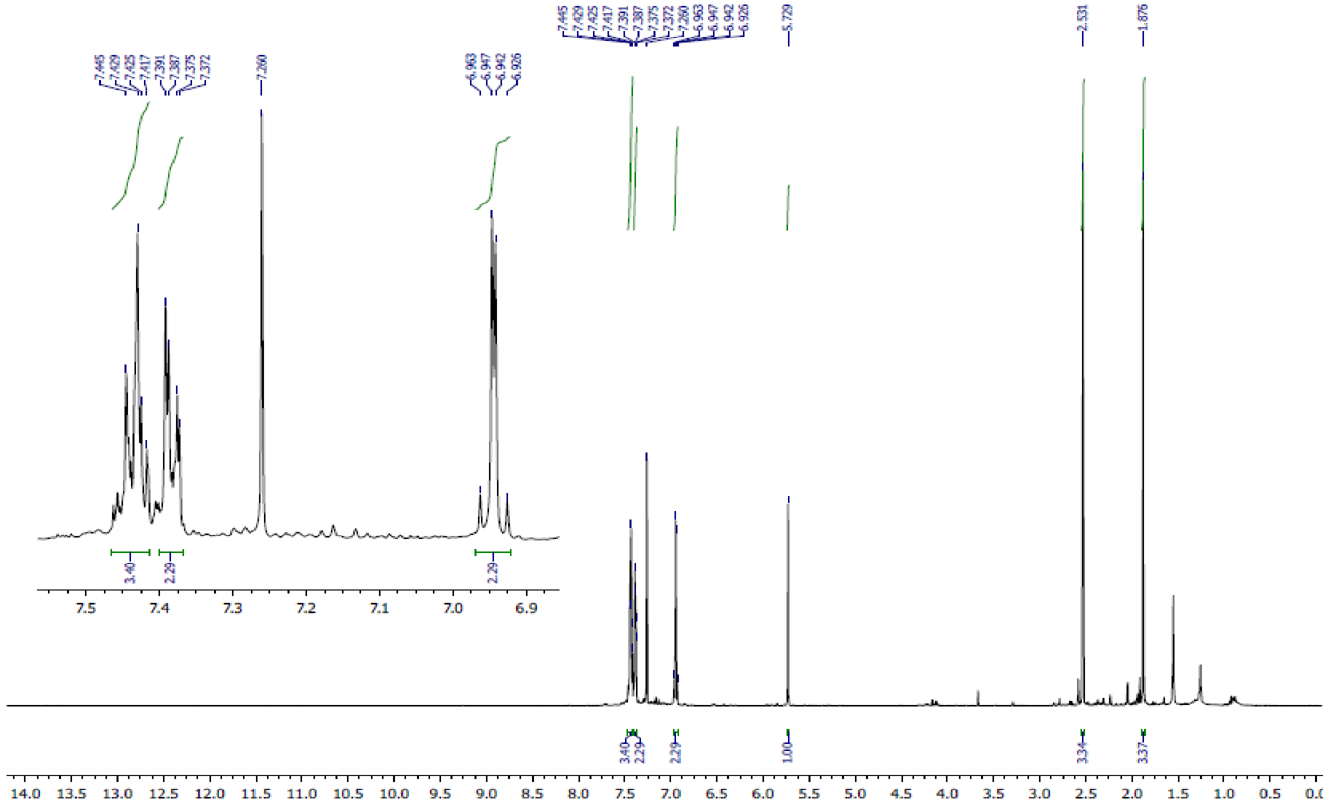

Figure S80. ^{1}H NMR spectrum of compound **3a** ($CDCl_3$, 400 MHz).

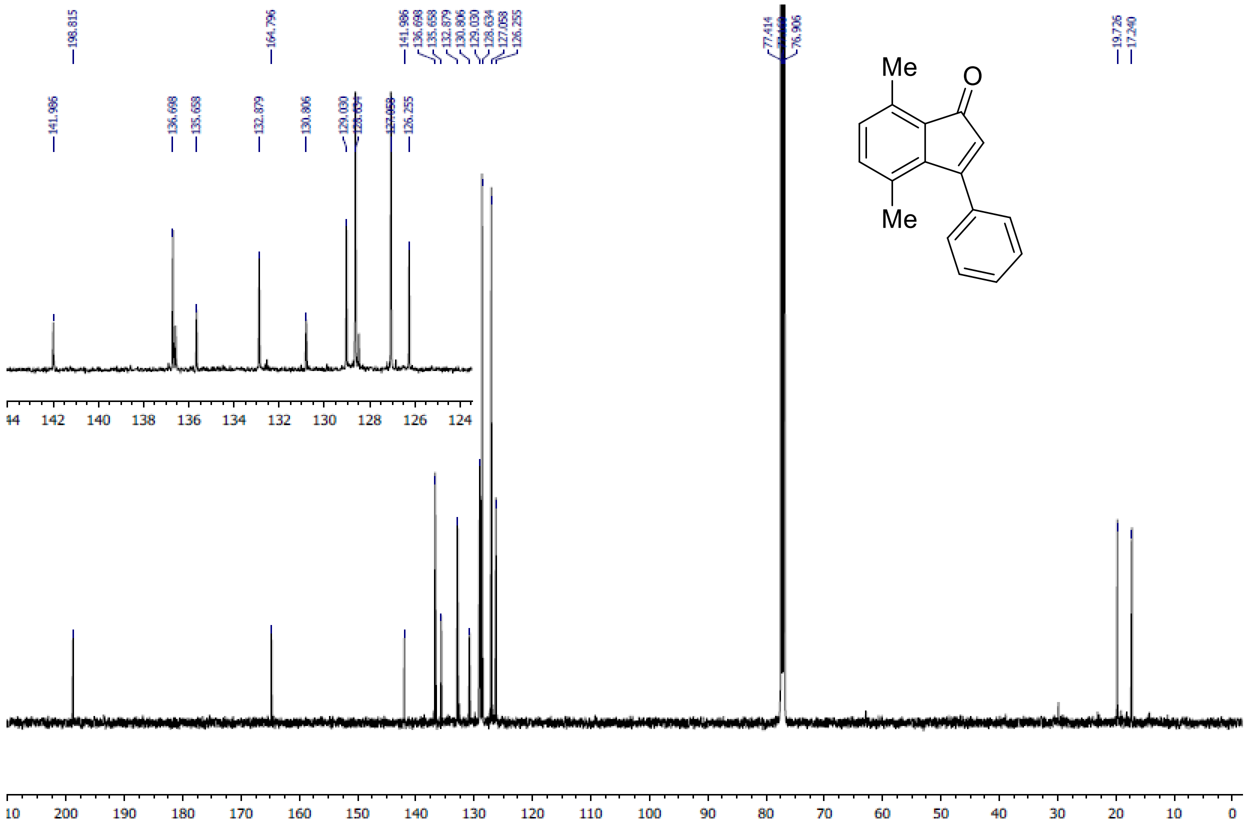

Figure S81. ^{13}C NMR spectrum of compound **3a** ($CDCl_3$, 100 MHz).

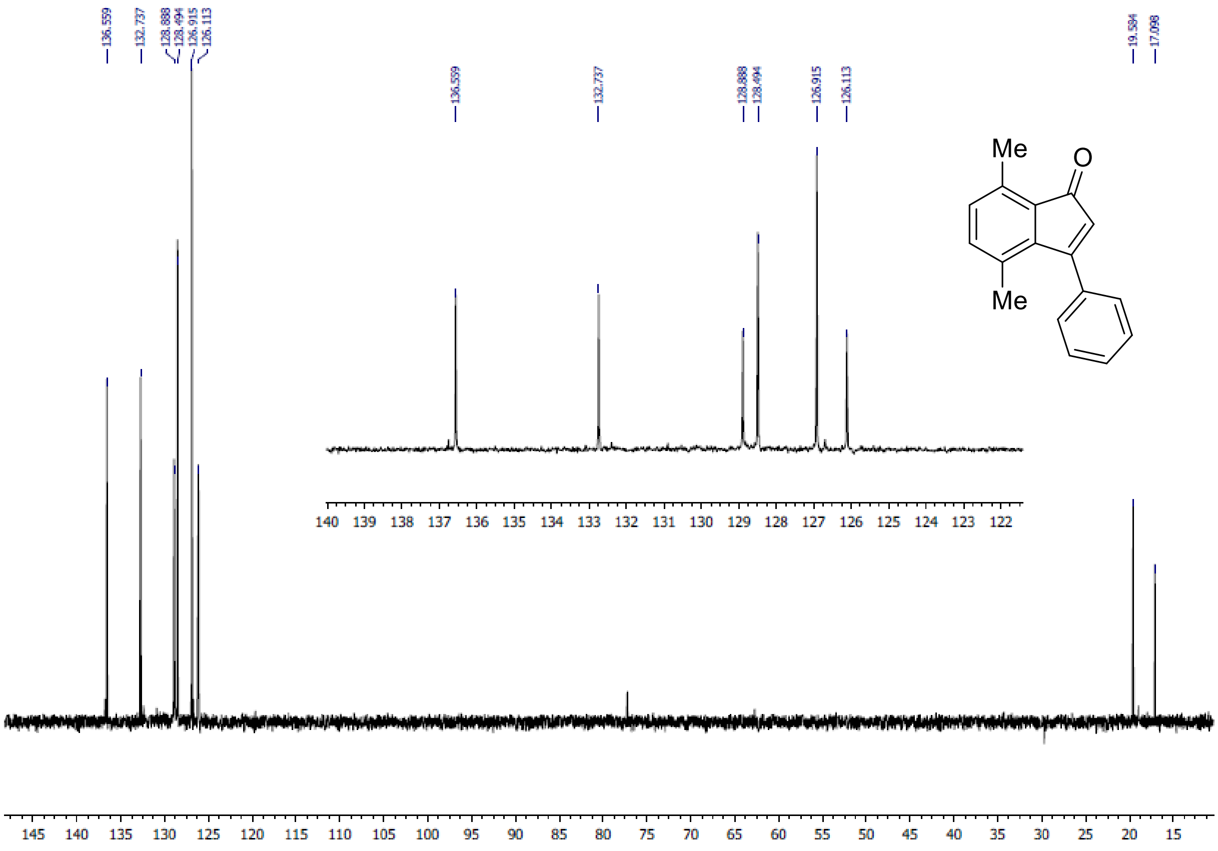

Figure S82. DEPT^{135} NMR spectrum of compound **3a** ($CDCl_3$, 400 MHz).

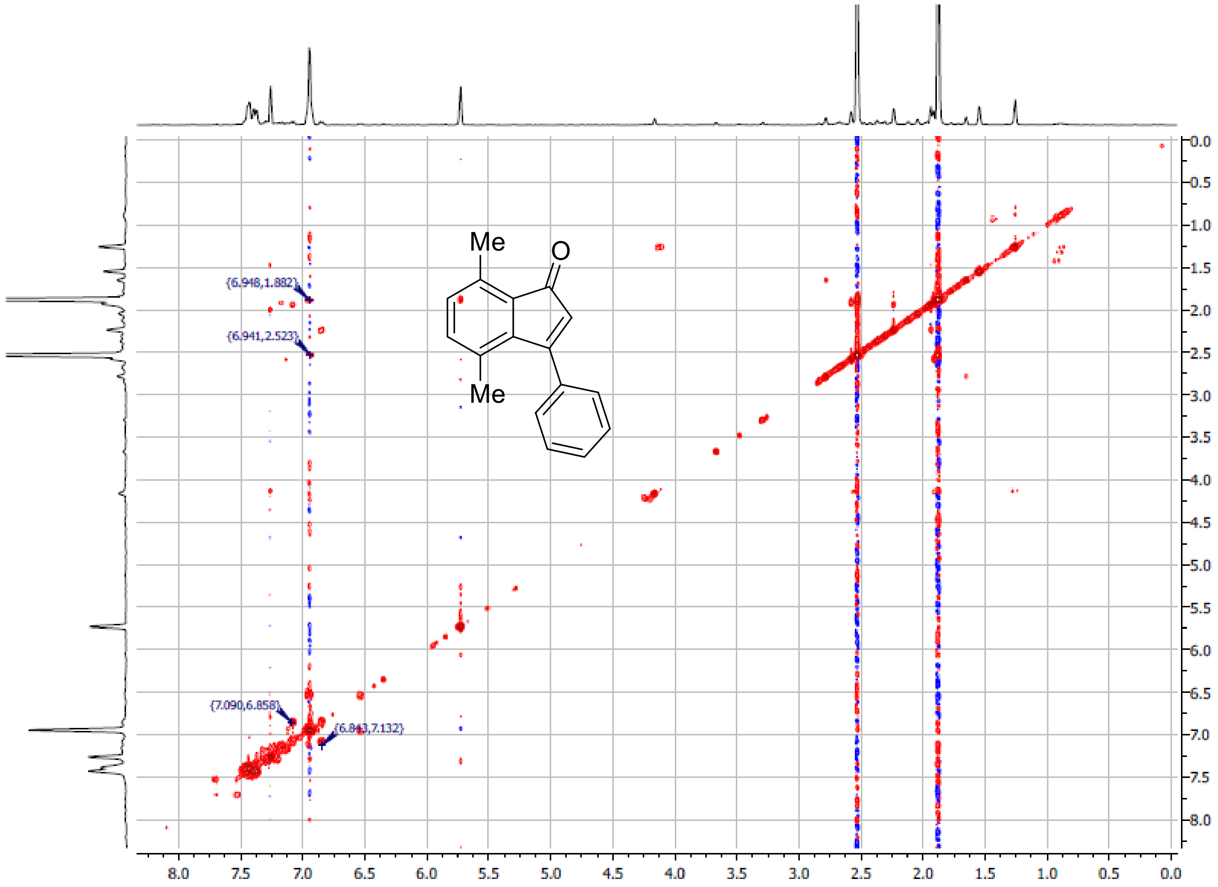

Figure S83. COSY NMR spectrum of compound **3a** ($CDCl_3$, 400 MHz).

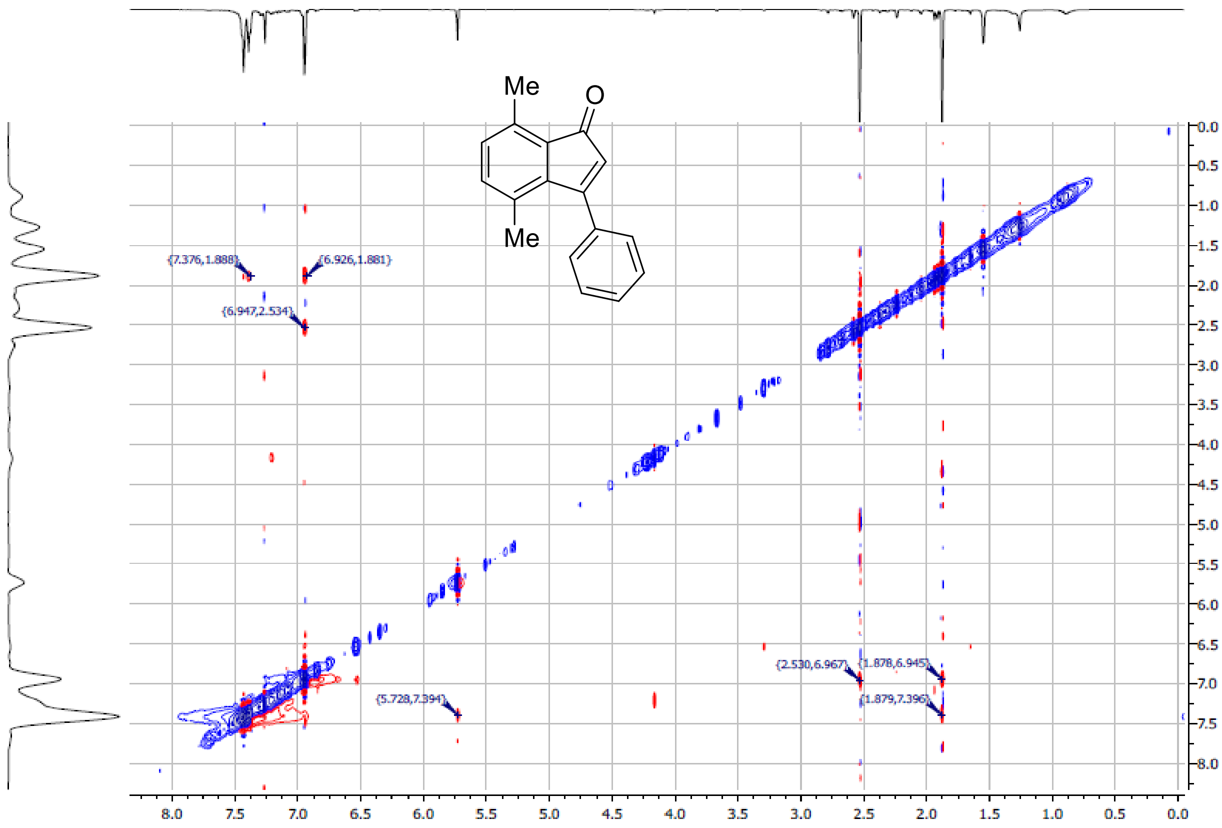

Figure S84. NOESY NMR spectrum of compound **3a** ($CDCl_3$, 400 MHz).

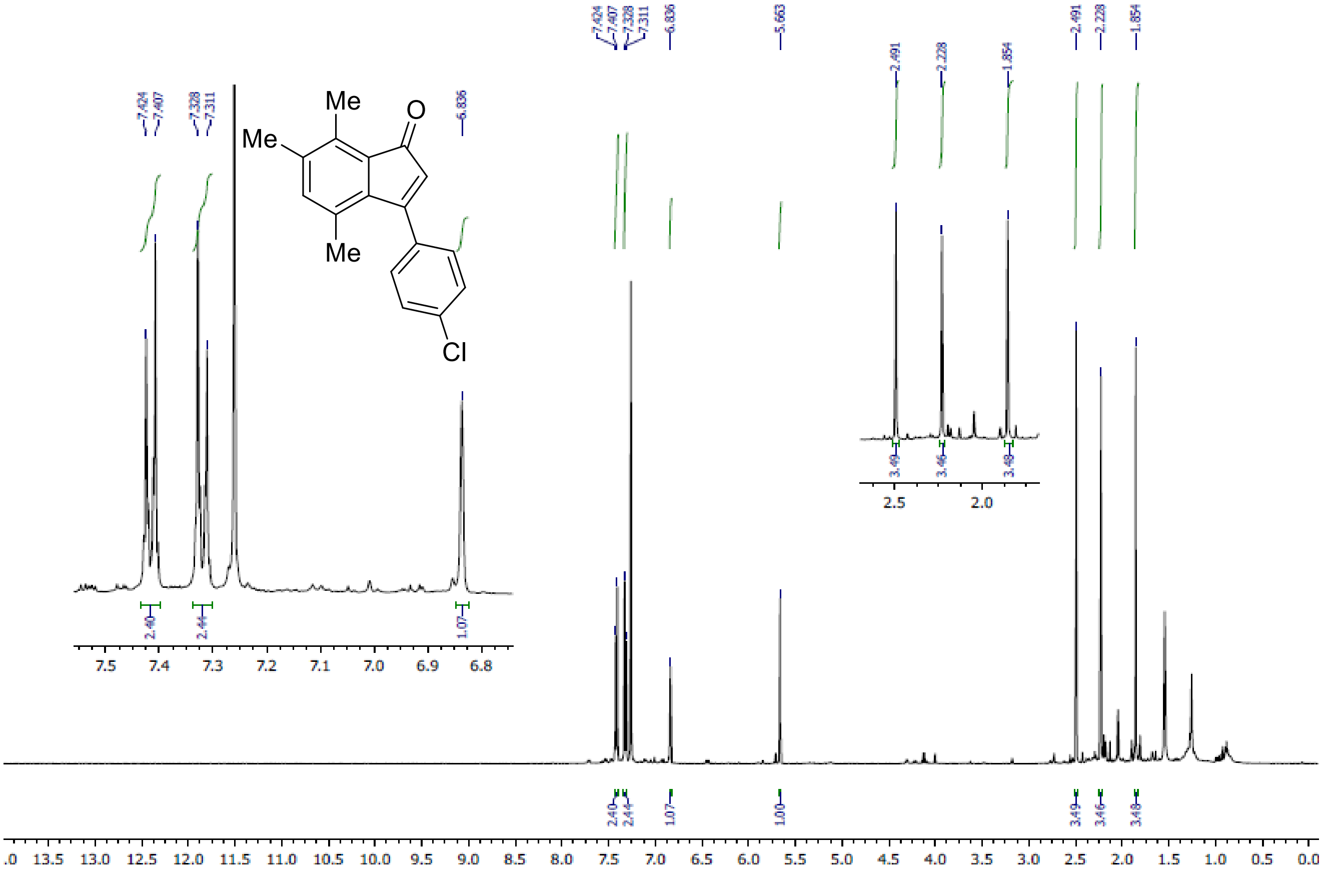

Figure S85. ^{1}H NMR spectrum of compound **3b** ($CDCl_3$, 400 MHz).

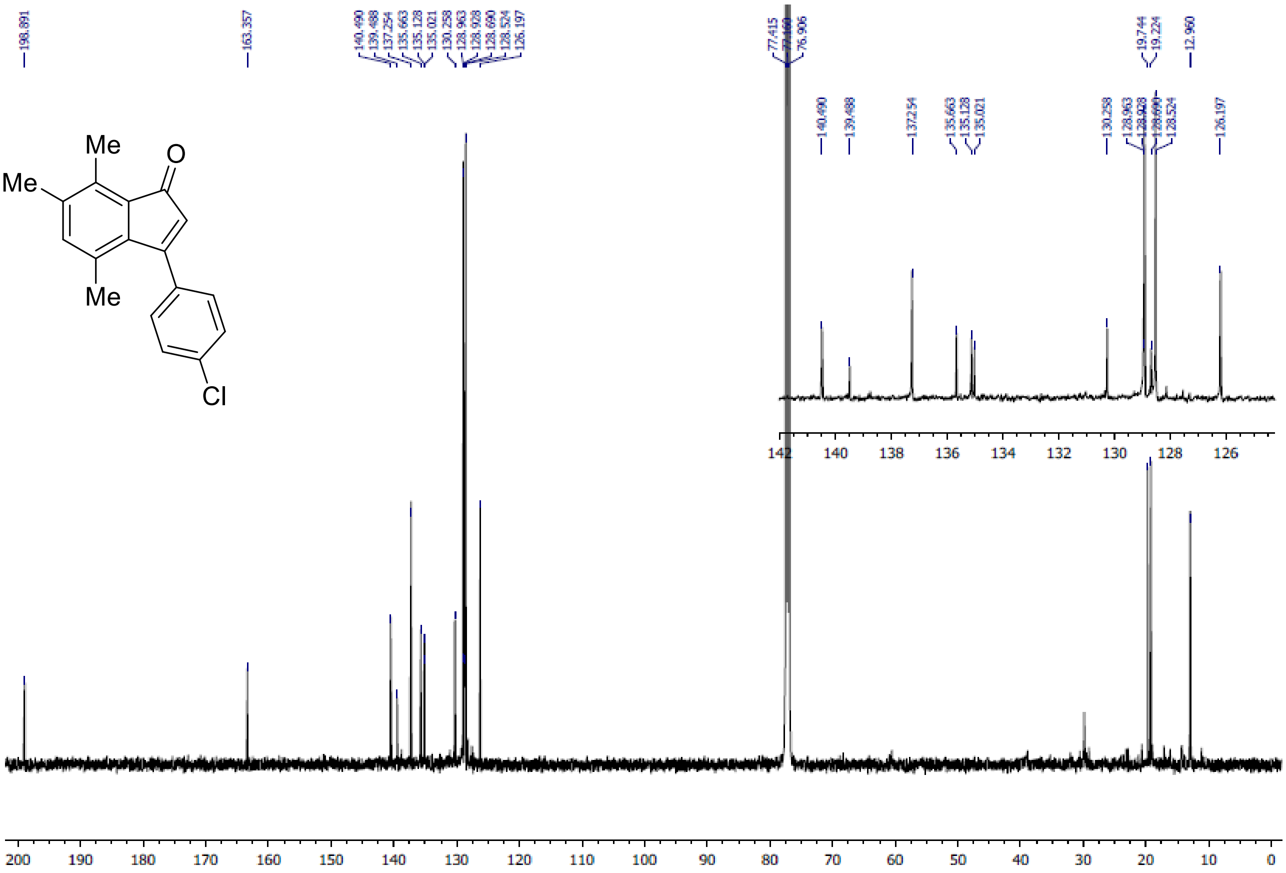

Figure S86. ^{13}C NMR spectrum of compound **3b** ($CDCl_3$, 100 MHz).

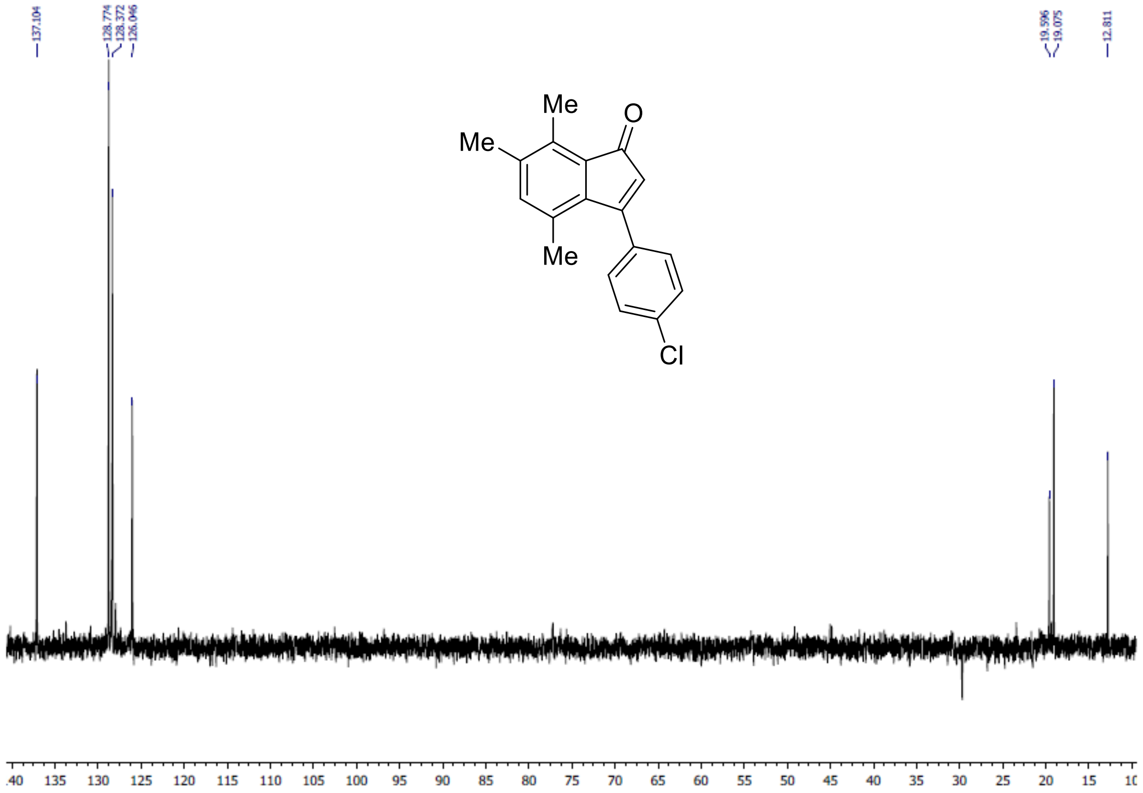

Figure S87. DEPT[135] NMR spectrum of compound **3b** ($CDCl_3$, 400 MHz).

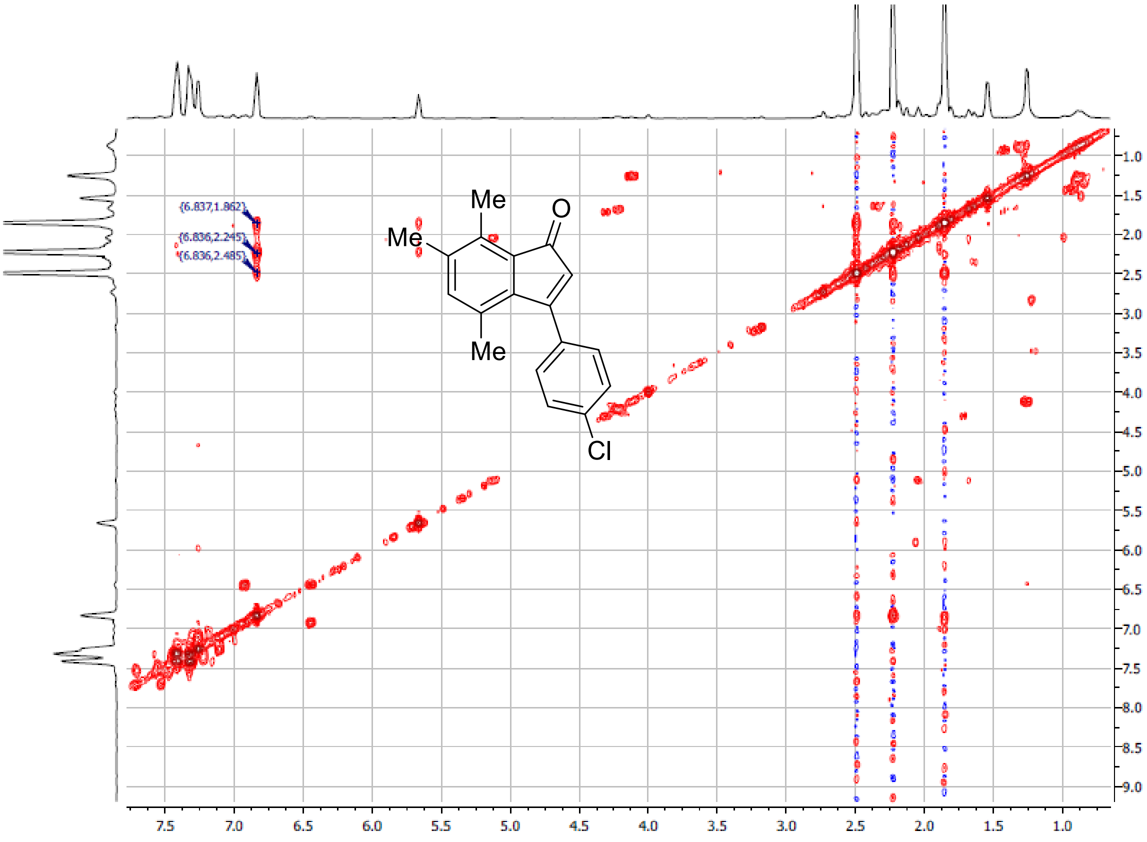

Figure S88. COSY NMR spectrum of compound **3b** ($CDCl_3$, 400 MHz).

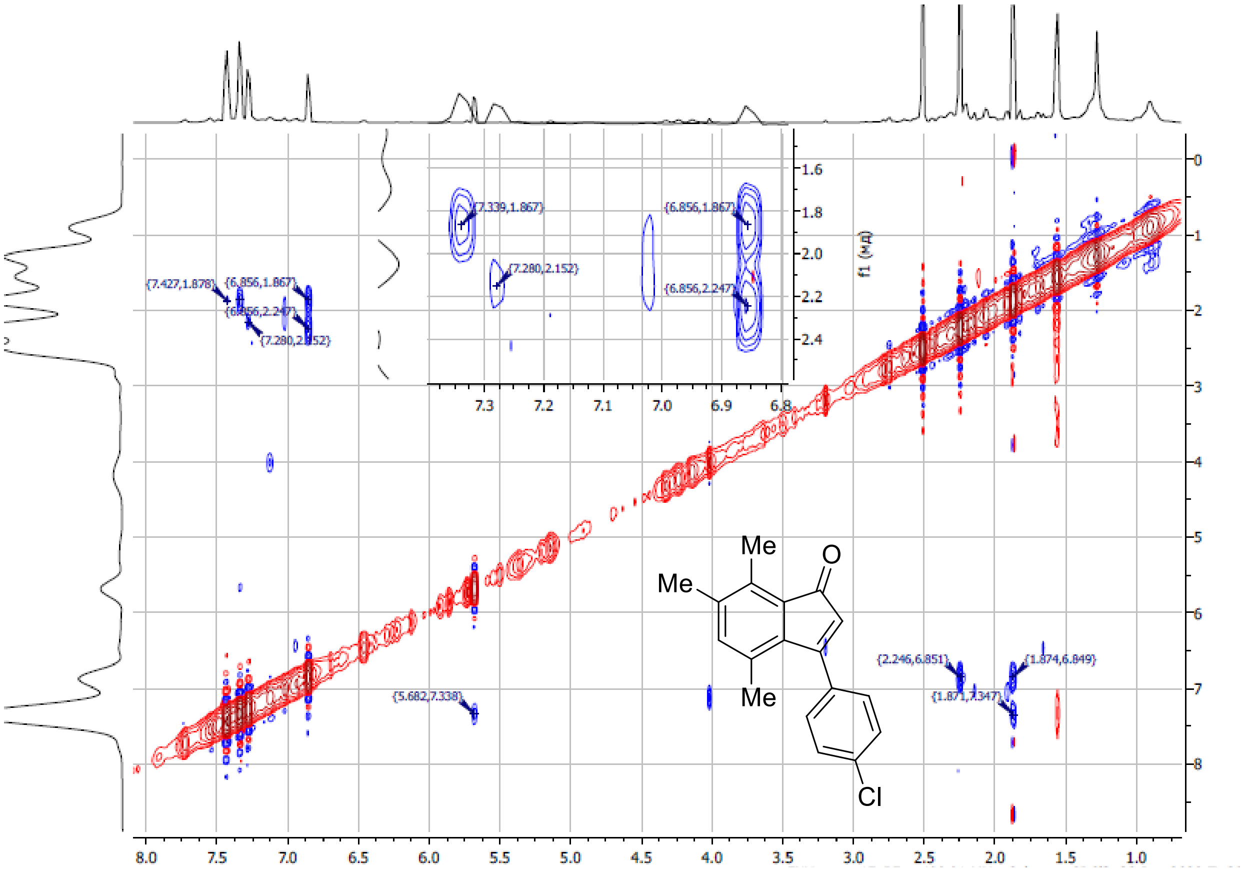

Figure S89. NOESY NMR spectrum of compound **3b** ($CDCl_3$, 400 MHz).
